# Extended Family of Thiophosphoryl-Appended Pd(II) Pincer Complexes with a Deprotonated Amide Core: Synthesis and Biological Evaluation

**DOI:** 10.3390/ijms26104536

**Published:** 2025-05-09

**Authors:** Diana V. Aleksanyan, Svetlana G. Churusova, Aleksandr V. Konovalov, Ekaterina Yu. Rybalkina, Lidia A. Laletina, Yana V. Ryzhmanova, Yulia V. Nelyubina, Svetlana A. Soloveva, Sergey E. Lyubimov, Alexander S. Peregudov, Zinaida S. Klemenkova, Vladimir A. Kozlov

**Affiliations:** 1 A. N. Nesmeyanov Institute of Organoelement Compounds, Russian Academy of Sciences, ul. Vavilova 28, Str. 1, Moscow 119334, Russia; s_churusova@rambler.ru (S.G.C.); prospectsovetov@gmail.com (A.V.K.); unelya@ineos.ac.ru (Y.V.N.); aksenova.sa@phystech.su (S.A.S.); lssp452@mail.ru (S.E.L.); asp@ineos.ac.ru (A.S.P.); zklem@ineos.ac.ru (Z.S.K.); fos@ineos.ac.ru (V.A.K.); 2 N. N. Blokhin National Medical Research Center of Oncology of the Ministry of Health of the Russian Federation, Kashirskoe Shosse 23, Moscow 115478, Russia; kate_rybalkina@mail.ru (E.Y.R.); panlidia@gmail.com (L.A.L.); 3 Skryabin Institute of Biochemistry and Physiology of Microorganisms, Pushchino Scientific Center of Biological Research, Russian Academy of Sciences, pr. Nauki 5, Pushchino 142292, Russia; ryzhmanova@gmail.com; 4 Federal Research Center of Problems of Chemical Physics and Medicinal Chemistry, Russian Academy of Sciences, pr. Akademika Semenova 1, Chernogolovka 142432, Moscow Region, Russia

**Keywords:** palladium, pincer complexes, metal-based drugs, cytotoxicity, antibacterial activity

## Abstract

The development of new, more effective, and selective anticancer agents is one of the most important tasks of modern medicinal chemistry. Recently, we have found that non-classical Pd(II) pincer complexes derived from thiophosphoryl-appended picolinamides exhibit promising cytotoxic properties. In this work, the potential of this class of metal-based derivatives was studied on an extended family of Pd(II) complexes with a deprotonated amide core featuring thiophosphoryl pendant arms, readily obtained by the direct cyclopalladation of new functionalized amide ligands upon interaction with PdCl_2_(NCPh)_2_ under mild conditions. The ligands, in turn, were obtained by conventional amide coupling methods using (aminobenzyl)- and (aminomethyl)diphenylphosphine sulfides as the key precursors and different *N*- and *S*-donor-substituted carboxylic acids. The effect of an acid component and carbon chirality in the ligand framework on the bioactivity of the resulting Pd(II) pincer complexes was elucidated by evaluating their cytotoxicity against different solid and blood cancer cell lines, apoptosis induction ability, and P-glycoprotein (P-gp) affinity, which revealed the high anticancer potential of some of them, and in particular, the potential to overcome drug resistance associated with P-gp overexpression. The representative palladocycle was also shown to possess moderate antibacterial activity.

## 1. Introduction

The increasing demand for chemotherapy prompts continuous research into the development of new potential anticancer agents [1]. One of the most popular groups of compounds at both the drug discovery and preclinical phases includes different platinum complexes, which is due to the key role of cisplatin, carboplatin, and oxaliplatin (and, more recently, several other platinum-based drugs) in modern cancer therapy [2]. However, the use of Pt(II) cytostatic agents is often associated with severe side effects and acquired resistance; therefore, the major challenge is the creation of more effective and selective anticancer drugs.

**Figure 1:**
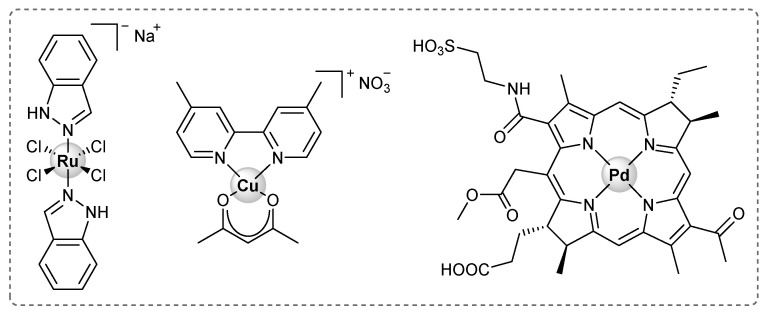
Transition metal complexes with remarkable anticancer properties.

Transition metal complexes have been extensively studied for their anticancer potential, and some non-platinum derivatives have already demonstrated encouraging results (for selected examples, see Figure 1) [3,4]. Particular attention has been given to Ru(II), Ru(III), Au(I), Au(III), Ir(III), Ti(IV), Cu(II), and Pd(II) compounds [4,5,6,7,8,9,10,11,12,13,14,15,16]. The latter compounds, although displaying coordination behavior similar to that of platinum counterparts, undergo much more rapid ligand exchange processes [17], which has compelled researchers to apply special design strategies. One of the recent trends in the development of metal-based cytotoxic agents, especially efficient in the case of Pd(II) derivatives [18,19,20,21,22,23,24,25,26], is the use of highly tunable tridentate pincer-type ligands that can provide high thermodynamic and controlled kinetic stability [27].

**Figure 2:**
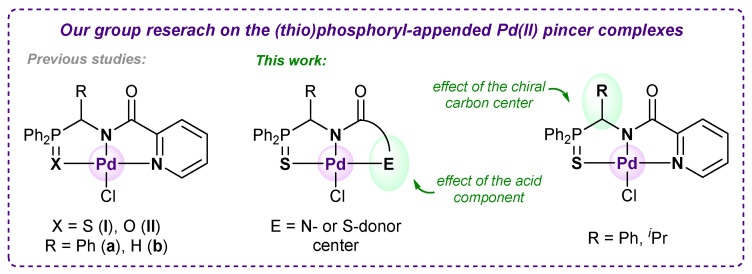
Cytotoxic thiophosphoryl-appended Pd(II) pincer complexes (our group research).

For the last several years, our research group has been engaged in a project on biologically active cyclometalated derivatives [18,21,28,29], which has resulted in the development of a new class of potential chemotherapeutics based on Pd(II) pincer complexes of functionalized amides (see reports [28,29] and the references cited therein). In particular, we have shown that (thio)phosphoryl-appended Pd(II) pincer complexes featuring a deprotonated picolinamide core possess remarkable cytotoxic activity against several human cancer cell lines (Figure 2) [28,29]. To further unleash the potential of this type of palladocycle, in this work, we scrutinized the effect of the most important structural aspects of thiophosphoryl-functionalized carboxamides, including stereochemistry, on the bioactivity of the resulting Pd(II) complexes, with particular emphasis on their ability to overcome drug resistance.

## 2. Results and Discussion

**Scheme 1:**
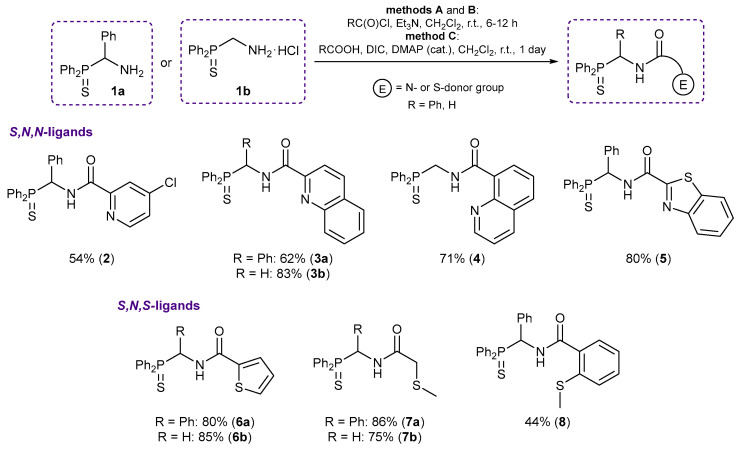
Synthesis of functionalized amides **2**–**8**.

Exploring the anticancer properties of palladocycles based on (thio)phosphoryl-functionalized picolinamides, earlier we focused our attention mainly on the effect of the amine component in the ligand framework on the stability and cytotoxic activity of the resulting complexes [28,29]. However, an acid component can also affect the lability of coordination of amide ligands by Pd(II) ions, thus giving way to further modulation of the biological properties of the target cyclopalladated derivatives. To assess the impact of the nature of an acid component, we expanded the family of non-classical amide-based pincer ligands with new representatives based on (aminobenzyl)diphenylphosphine sulfide **1a** and its analog **1b** bearing an unsubstituted methylene bridge between the thiophosphoryl and amino groups. Using conventional protocols for amide coupling, a series of thiophosphoryl-appended amides with mixed *S*,*N*,*N-*donor sets were obtained from 4-chloropicolinic, isomeric quinoline-2- and quinoline-8-carboxylic, as well as benzo[*d*]thiazole-2-carboxylic acids, which can ostensibly provide hemilabile coordination (compounds **2**–**5**, Scheme 1). The latter was ascertained as a positive factor for the bioactivity of related *N*-metalated Pd(II) pincer complexes featuring *S*-donor amino acid residues (see the references mentioned in [28,29]). The presence of an additional conjugated aromatic ring in the *N*-heterocyclic unit may influence the affinity of the ensuing metal complexes to a potential biological target owing to the possibility of facilitating noncovalent stacking interactions. The introduction of a chlorine atom into the pyridine ring, in turn, can increase lipophilicity and facilitate the implementation of additional nonbonding interactions in the binding site [30]. For comparison, several *S*-donor carboxylic acids featuring either an aliphatic or (hetero)aromatic core were also reacted with the key thiophosphorylated amines to give *S*,*N*,*S*-ligands **6**–**8** (Scheme 1).

**Scheme 2:**
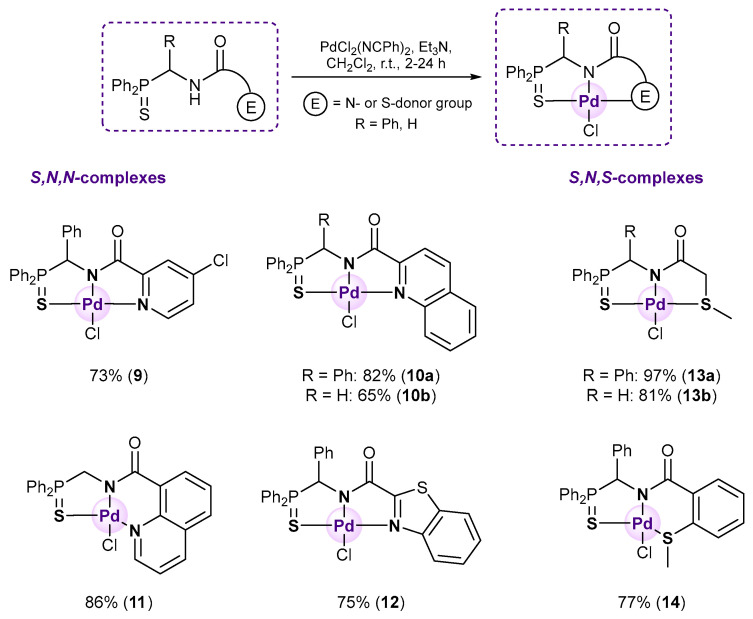
Synthesis of Pd(II) pincer complexes **9**–**14**.

Most of the ligands obtained readily underwent direct cyclopalladation upon interaction with PdCl_2_(NCPh)_2_ in dichloromethane at room temperature. The only exceptions were thiophene-containing derivatives **6a**,**b** that formed a mixture of non-identified coordination complexes with the same Pd(II) precursor under mild conditions, which rapidly decomposed upon short-term heating, resulting in the release of palladium black. This can be explained by the weak donor ability of a sulfur atom incorporated into the heterocyclic unit. Nevertheless, in the other cases, irrespective of the nature and length of the coordination arms, the target pincer complexes were isolated in good to high yields after chromatographic purification (compounds **9**–**14**, Scheme 2).

The identities and structures of both free amides **2**–**8** and their cyclopalladated derivatives were unambiguously confirmed based on the IR and multinuclear NMR spectroscopic data (including different 2D NMR techniques), as well as elemental analyses, which are presented in detail in Section 3 (Materials and Methods) and Figures S1–S31 in the Supporting Information (SI). The realization of metalation of the central secondary amide unit in the resulting complexes was supported by the lack of NH proton singlets in the ^1^H NMR spectra, as well as the absence of amide II and amide A bands in the IR spectra. The coordination of the thiophosphoryl pendant arm was evidenced by a strong downfield shift of the phosphorus resonance (Δ*δ*_P_ = 8.1–17.4 ppm) and the displacement of the P=S bond stretches towards lower frequencies, which reached up to 65 cm^−1^. The coordination of the additional *N*- or *S*-donor unit was indirectly indicated by considerable shifts of the signals of CH/CH_3_ protons or quaternary carbon nuclei located in the close proximity to the heteroatom (for example, Δ*δ*_H_ for the methyl substituent in the thioether group of **13a**,**b** was more than 0.65 ppm, while a coordination shift of the proton signal, corresponding to the CH unit adjacent to the nitrogen atom of the pyridine ring of **9**, composed 0.45 ppm). It is also noteworthy that the complexes obtained are stable in solution, even in a strongly coordinating medium such as DMSO, at least for several days, which was demonstrated by ^31^P NMR monitoring using compounds **9** and **10b** as representative examples (see Figure S32 in the SI).

**Figure 3:**
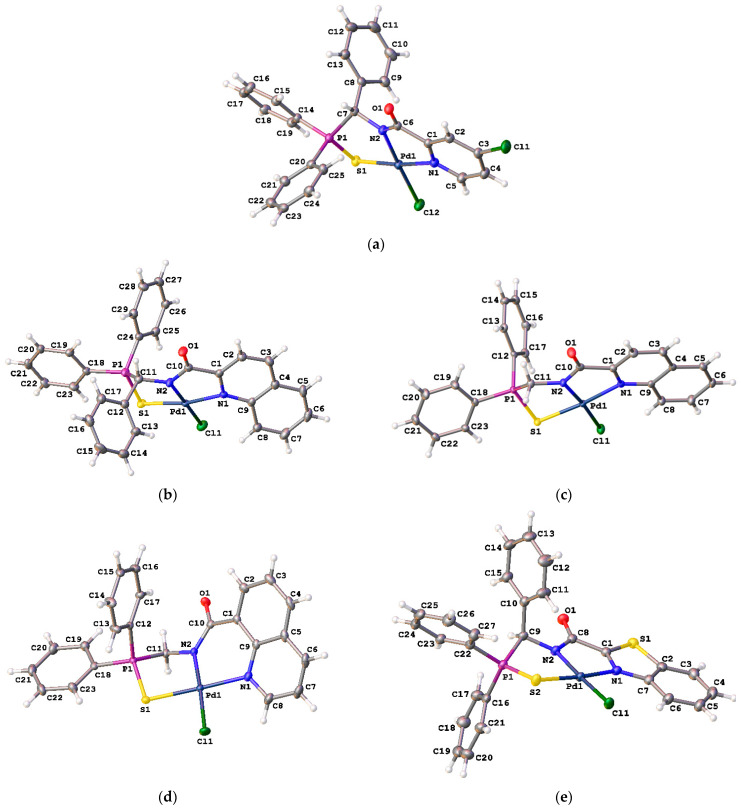

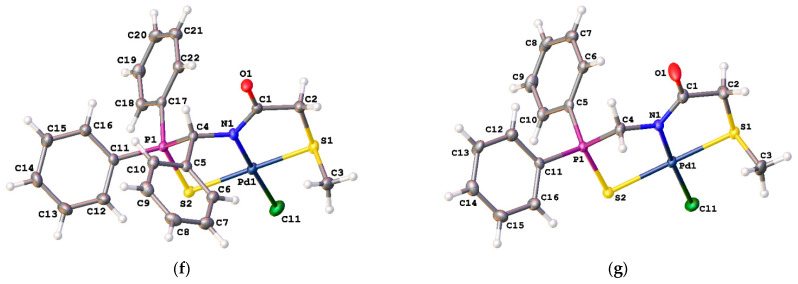
Molecular structures of complexes **9** (**a**), **10a** (**b**), **10b** (**c**), **11** (**d**), **12** (**e**), **13a** (**f**), and **13b** (**g**). Hereinafter, the non-hydrogen atoms are shown as thermal ellipsoids at a 50% probability level. The solvent molecule of dichloromethane for 10b and the disordered lattice molecule of ethanol for **13a** are omitted for clarity.

The structures of almost all the palladocycles (Figure 3) and ligand **8** (Figure S33 in the SI) were corroborated by single-crystal X-ray diffraction analysis. Table S1 in the SI lists the main geometrical parameters of the complexes explored. In all cases, the palladium atom features a slightly distorted square-planar environment formed by four different donor centers: the chloride anion, the nitrogen atom of the deprotonated amide unit, the sulfur atom of the thiophosphoryl group, and the nitrogen or sulfur atom of the second ancillary donor group. This unsymmetrical surrounding leads to considerable distortions from a planar conformation of most of the five-membered metal-containing rings. Nevertheless, the bond lengths and angles involving the metal center are only slightly affected by the sizes of the fused metallocycles and are within the expected ranges for this type of Pd(II) complexes [28,29], for example, Pd–Cl 2.3018(4)–2.3460(5), Pd–S(P) 2.2696(5)–2.3490(9), Pd–N(amide) 1.9926(17)–2.0152(16). The analysis of the supramolecular organization in the crystals revealed, besides the typical intermolecular interactions, C–H···Cl and C–H···S contacts, which in some cases connect the complex molecules into dimers.

The cytotoxic activity of the complexes obtained was screened against several human solid and hematopoietic cancer cell lines using the conventional MTT assay. To evaluate their selectivity for cancerous over non-cancerous cells, analogous tests were performed with pseudonormal human embryonic kidney (HEK293) and breast epithelial (HBL100) cell lines. The half-maximal inhibitory concentrations determined after 48 h of exposure of most of the cell cultures to the compounds under consideration and the corresponding data for cisplatin used as a positive control are summarized in Table 1 and Table 2.

The results obtained on solid cancer cell lineages suggest that *S*,*N*,*S*-pincer complexes **13a**,**b** derived from methylthioacetamides (Table 1, entries 7, 8) and their *S*,*N*,*N*-analogs with quinoline units (entries 3–5) possess only moderate efficiency, in some cases being inferior to cisplatin (entry 12). Methylthiosalicylamide-based analog **14**, although demonstrating pronounced effects on all of the cancer cell lines explored, was highly toxic towards non-cancerous HEK293 cells. This shortcoming was also detected in the case of benzothiazole-containing derivative **12** (Table 1, entry 6). In general, the *S*,*N*,*N*-palladocycles with fused heterocyclic units were less active than their pyridine-based counterparts (cf. entries 3–6 with 1, 2). The values of IC_50_ for the latter were observed, as a rule, in the low micromolar range. It is noteworthy that the introduction of a chlorine substituent into the pyridine ring allowed for increased efficiency against MCF7 breast cancer cells, which usually display low sensitivity to this type of Pd(II) complex [28,29]. More importantly, 4-chloropicolinamide-based palladocycle **9** exerted considerably higher cytotoxic effects than the previously reported unsubstituted derivative **Ia** against glioblastoma (U251) and ovarian adenocarcinoma (Scov3): the values of IC_50_ on the brain and ovarian cancer cells were 9.0 ± 1.0 vs. 34.0 ± 4.0 and 8.0 ± 0.7 vs. 30.0 ± 2.0 μM for complexes **9** and **Ia**, respectively.

The experiments on blood cancer cells revealed the same structure–activity relationships as in the case of the solid cancer cell lines, with methylthioacetamide derivative **13a** being the least active complex from this study (Table 2, entry 4). The highest sensitivity was demonstrated by multiple plasmacytoma (AMO1) and acute lymphoblastic leukemia (H9) cells, for which the values of IC_50_ ranged within a narrow micromolar interval (1.2–4.2 μM). This ensured slightly higher selectivity of the complexes under investigation to cancerous vs. non-cancerous cells.

It should be noted that, as well as in the case of their picolinamide prototypes [28], free thiophosphoryl-appended amides **2**–**8** were not toxic even at a concentration as high as 60 μM, suggesting that the antiproliferative properties of their cyclopalladated derivatives are stipulated mainly by coordination with Pd(II) ions. The starting cyclometalation agent (PdCl_2_(NCPh)_2_) was also inactive at concentrations up to 60 (in the case of solid cancer cells) and 40 (in the case of hematopoietic cancer cells) μM. Furthermore, the complexes obtained demonstrated close levels of activity towards parental and doxorubicin-resistant cell lines. In the case of non-cancerous mammary epithelial cells HBL100 and HBL100/Dox, the difference in the values of IC_50_ did not exceed 2.1, while doxorubicin was more than 80 times less active against the resistant clone [31]. For the doxorubicin-resistant subline of chronic myelogenous leukemia cells (K562/iS9), the efficiency of most of the complexes obtained in this work was even higher than against parental K562 cells, while the efficiency of the organic chemotherapeutic agent decreased by a factor of 20 [31]. This implies a great potential of this type of promising metal-based cytotoxic agents to overcome drug resistance, which will be considered further.

**Scheme 3:**
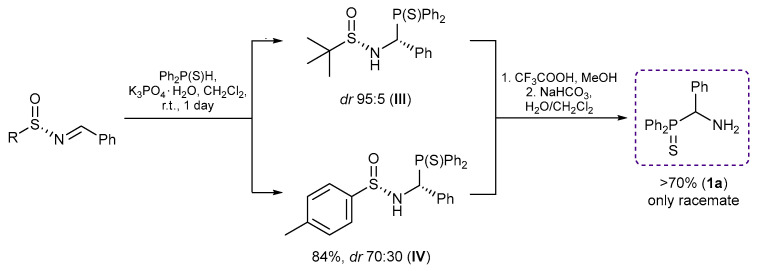
Synthesis of amine 1a from sulfinamide derivatives.

The results on cytotoxicity studies with an extended panel of Pd(II) pincer complexes featuring a thiophosphoryl-appended amide core confirmed our previous observation that the aminobenzyl-based derivatives in general exhibit higher efficiency than their unsubstituted counterparts. In this respect, another important structural feature of this class of potential chemotherapeutic agents that deserves special attention is the presence of a chiral carbon center. Taking into account the high requirements for stereoselectivity in modern drug design, it seemed important to probe the asymmetric synthesis of (aminobenzyl)diphenylphosphine sulfide **1a** that would ensure the production of enantiomerically pure functionalized amide ligands on its basis. For this purpose, a Pudovik-type reaction of diphenylphosphine sulfide with an imine substrate bearing a stereodirecting group, instead of achiral hydrobenzamide, which was used by us earlier, seemed to be a good choice. Recently, Zhang and Gilbertson [32] demonstrated the possibility of the stereoselective nucleophilic addition of Ph_2_P(S)H to optically active *N*-*tert*-butylsulfinyl imines in the presence of potassium phosphate. We reproduced the synthesis of thiophosphoryl-functionalized *tert*-butylsulfinamide derivative **III** and obtained its *p*-tolyl-substituted analog **IV** under similar conditions, albeit with slightly lower selectivity in the latter case, but unfortunately, further attempts to hydrolyze the sulfinyl group in both substrates afforded amine **1a** only in the racemic form (Scheme 3).

**Scheme 4:**
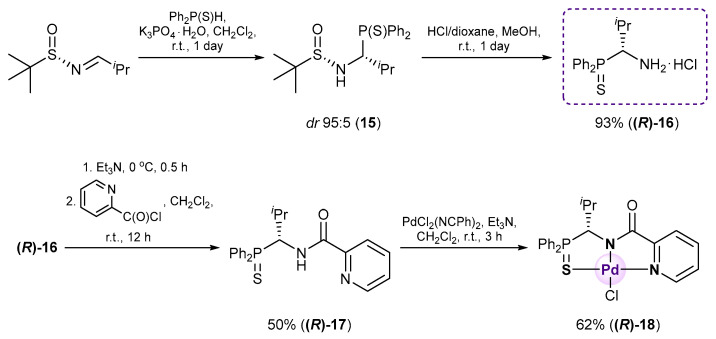
Synthesis of enantiomerically pure isopropyl-substituted derivatives (***R***)-**16**–**18**.

**Figure 4:**
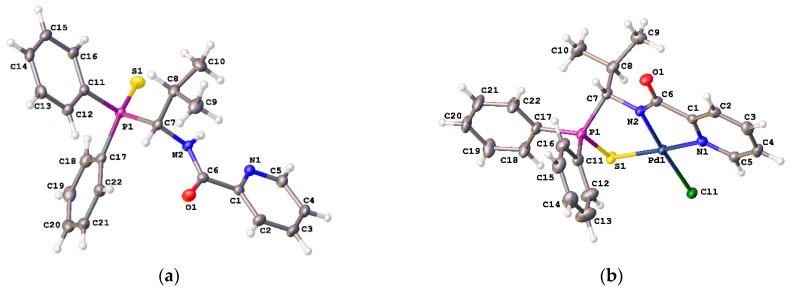
The molecular structures of ligand (***R***)-**17** (**a**) and its cyclopalladated derivative (***R***)-**18** (**b**). The second symmetry-independent molecule of the complex is not shown.

In order to reduce the acidity of the C–H bond in the P(S)-amine bridging unit, we decided to target the synthesis of an isopropyl-substituted analog of compound **1a**. The treatment of sulfinamide **15** with HCl in dioxane yielded an individual stereoisomer of the desired thiophosphorylated amine in the form of a hydrochloride salt (compound (***R***)-**16**) (Scheme 4). Note that in our hands the free thiophosphorylated amine with an isopropyl substituent in the bridging unit underwent partial racemization even under mild conditions (e.g., upon treatment of (***R***)-**16** with Et_3_N in benzene at room temperature for 30 min, the value of *ee* composed 42% (Figure S34 in the SI)). Nevertheless, the acylation of the isopropyl-substituted amine in situ generated from its hydrochloride salt (***R***)-**16** under the action of picolinoyl chloride smoothly afforded amide (***R***)-**17** in an enantiomerically pure form (Scheme 4). The formation of a single stereoisomer was confirmed by chiral HPLC analysis using a Chiralcel OD column (see Figure S35 in the SI), and its absolute configuration was deduced from XRD measurements (Figure 4a). The direct cyclopalladation of ligand (***R***)-**17** was readily accomplished under conditions analogous to the synthesis of palladocycles **9**–**14**, furnishing target Pd(II) pincer complex (***R***)-**18** in a moderate yield. Figure 4b depicts the molecular structure of the resulting palladocycle. Table S1 in the SI lists its main geometrical parameters. Figures S36–S57 in the SI present the NMR spectra (including different 2D spectra) of the isopropyl-substituted ligand and its complex with full assignment. In addition, using the racemic sample of amine hydrochloride **16**, the corresponding amide (**17**) and its cyclopalladated derivative (**18**) were obtained in the racemic forms; the structures of compounds **16** and **18** were also supported by X-ray crystallography (Figure S58 in the SI).

**Figure 5:**
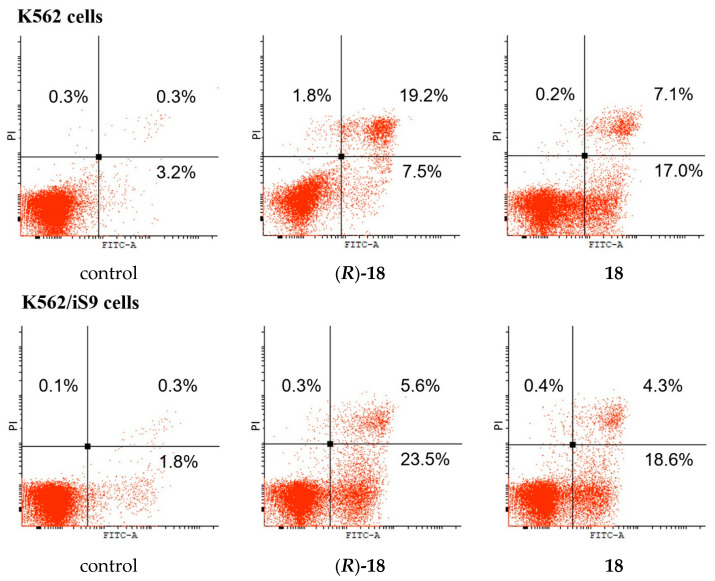
The percentages of necrotic (upper left), early apoptotic (lower right), and late apoptotic (upper right) K562 and K562/iS9 cells in the control experiments and after exposure to complexes (***R***)-**18** and **18** for 20 h.

To our delight, in general the biological profile of complex (***R***)-**18** appeared to be close to that of the previously reported phenyl-substituted counterpart (compare entries 10 and 1 in Table 1 and entries 6 and 1 in Table 2), with a lower activity on HCT116 and PC3 cells but remarkably higher selectivity towards K562 cells over non-cancerous HEK293 cells. This certainly justifies the structural modification performed in pursuit of the asymmetric synthesis of the target molecules: palladocycle (***R***)-**18** exhibited a high level of cytotoxicity against both the solid and blood cancer cell lines explored, ranking as one of the most promising candidates among the thiophosphoryl-appended Pd(II) pincer complexes. Interestingly, the enantiomerically pure compound exerted slightly better effects on malignant cells than the racemic sample **18** (entries 10 vs. 11 in Table 1, entries 6 vs. 7 in Table 2). Of note are the comparable levels of activity of both palladocycles against breast epithelial (HBL100) and chronic myelogenous leukemia (K562) cells and their doxorubicin-resistant clones (HBL100/Dox and K562/iS9, respectively), which were already observed for other thiophosphoryl-containing amide-based Pd(II) pincer complexes. These results were further confirmed by flow cytometry analysis of the apoptosis-inducing ability in parental (K562) and resistant (K562/iS9) sublines performed for both enantiomerically pure and racemic palladocycles 18 (for the corresponding double staining Annexin V-FITC/PI images, see Figure 5). The total percentage of early (lower right quadrant) and late (upper right quadrant) apoptotic cells for (***R***)-**18** on K562/iS9 subline was even higher than in the parental cells. These findings prompted us to gain further insight into the bioactivity of the Pd(II) pincer complexes of thiophosphoryl-functionalized amides from the viewpoint of their potential to overcome drug resistance.

**Figure 6:**
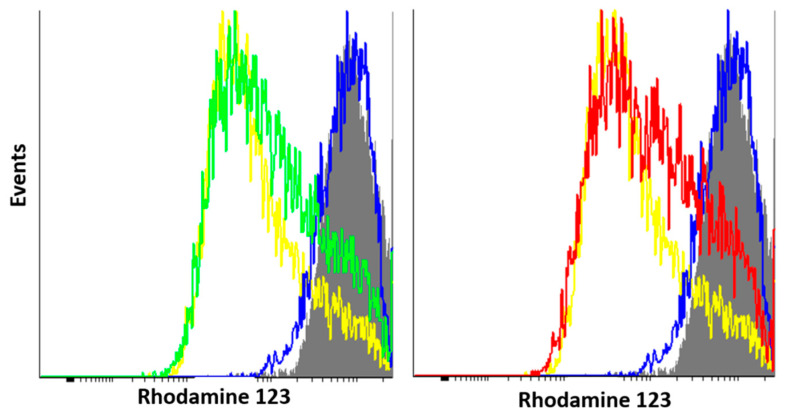
Rh123 efflux from K562/iS9 cells: after staining with the dye (grey solid background), efflux in the fresh medium (yellow) and in the media with elacridar (blue) or complexes (***R***)-**18** (red) and **18** (green).

The long-term use of cytotoxic agents can lead to the development of drug resistance in tumor cells. In most cases, such resistance occurs due to the activation of P-glycoprotein (P-gp). This is an ATP-dependent transmembrane protein that actively removes a large variety of compounds from cells, including many drugs. The active efflux hampers the achievement of toxic concentrations by drugs. Cells in which P-gp is overexpressed can acquire resistance simultaneously to a number of cytotoxic agents. This phenomenon, called multiple drug resistance, represents a serious challenge in chemotherapy. One way to solve this problem is to develop drugs that do not serve as substrates for this protein. Therefore, it seemed interesting to evaluate the efficiency of the binding of the thiophosphoryl-appended Pd(II) pincer complexes under consideration with P-gp. For this purpose, compounds (***R***)-**18** and **18** were chosen as the representative examples. We assessed the efflux of the fluorescent dye Rhodamine 123 (Rh123) from cells with a high P-gp content—K562/iS9 cells [31]. Figure 6 shows the diagrams of Rh123 efflux during cell incubation in the medium with elacridar, used as a positive control, and complexes (***R***)-**18** and **18**.

As can be seen, elacridar, known as an effective P-gp inhibitor, almost completely blocks the efflux of Rh123, and the cell fluorescence reduces only slightly. The yellow diagram corresponds to the efflux in the pure medium; under these conditions, rhodamine is actively removed from the cells, and their fluorescence is significantly reduced. When incubated in the media with palladocycles **18**, the fluorescence approached the values observed in the fresh medium. This indicates that these compounds do not interact with P-gp and slightly modulate its activity. In addition, they also do not affect the cytotoxicity of doxorubicin against K562/iS9 and HBL-100/Dox when being incubated with this drug at subtoxic concentrations (2–5 μM) (Figure S59 in the SI). Hence, the compounds explored do not serve as substrates for P-gp. In general, Pd(II) pincer complexes of thiophosphoryl-functionalized amide ligands hold great promise for the development of potential anticancer agents that would be able to surmount drug resistance associated with P-gp overexpression.

Last but not least, complex **18** tested in the racemic form exhibited moderate antibacterial activity against *Micrococcus luteus* at a concentration of 2.50 mM and retained some efficiency against both *Micrococcus luteus* and *Bacillus subtillis* even at a lower concentration of 0.25 mM in the agar well diffusion experiments (Table 3). As well as in the case of cytotoxicity studies, free amide **17** did not exert any inhibitory effect on microorganisms, suggesting that the complex activity is stipulated by the presence of coordinated transition metal ions. This opens up new prospects for further bioactivity studies of Pd(II) pincer complexes based on thiophosphoryl-functionalized amides.

## 3. Materials and Methods

### 3.1. General Remarks

Unless specifically stated, all manipulations were carried out in the normal atmosphere without taking precautions to exclude air and moisture. Dichloromethane was distilled from P_2_O_5_. Triethylamine was distilled over sodium. The key thiophosphorylated amine derivatives were obtained according to the earlier developed methods by the addition of Ph_2_P(S)H to hydrobenzamide, followed by acid hydrolysis (**1a**) or the sequential transformations of (hydroxymethyl)diphenylphosphine sulfide (**1b**) [28]. 4-Chloropicolinoyl chloride was synthesized from picolinic acid and SOCl_2_ in the presence of NaBr [33] and was used directly in the next step without purification. Quinoline-2- and quinoline-8-carbonyl chlorides were obtained from the corresponding acids and SOCl_2_, and were also used directly in the amide couplings [34]. Picolinoyl chloride was generated in situ through the reaction of picolinic acid with SOCl_2_ in the presence of Et_3_N [35]. (*S*)-*N*-[(*R*)-1-(Diphenylthiophosphoryl)-2-methylpropryl]-2-methylpropane-2-sulfinamide **15** was obtained by the nucleophilic addition of Ph_2_P(S)H to (*S*)-*N*-*tert*-butylsulfinyl imine in the presence of potassium phosphate [32]. All other chemicals and solvents were used as purchased.

The NMR spectra were recorded on Bruker Avance 300, Avance 400, and Avance 500 spectrometers (Bruker AXS GmbH, Karlsruhe, Germany), and the chemical shifts (*δ*) were referenced internally by the residual (^1^H) or deuterated (^13^C) solvent signals relative to tetramethylsilane or externally to H_3_PO_4_ (^31^P). In all cases, the ^13^C{^1^H} NMR spectra were registered using the *J*MODECHO mode; the signals for the *C* nuclei bearing odd and even numbers of protons had opposite polarities. The NMR peak assignments for ligands **8** and **17** and complexes **9**, **14**, and **18** were based on the analysis of the ^1^H–^1^H-COSY and ^1^H–^13^C HSQC or HMQC, and ^1^H–^13^C HMBC spectra. The assignments for the other compounds obtained in this study were based on the resulting or previously reported data [28]. For the NMR spectra of the representative ligands and their cyclopalladated derivatives, see Figures S1–S31 and S36–S57 in the SI.

The IR spectra were recorded on a Nicolet Magna-IR750 FT spectrometer (Nicolet, Madison, WI, USA) (resolution 2 cm^−1^, 128 scans). The assignment of absorption bands in the IR spectra was made according to Ref. [36].

Column chromatography was carried out using Macherey-Nagel silica gel 60 (MN Kieselgel 60, 70–230 mesh) (Macherey-Nagel, Dueren, Germany). The values of enantiomeric excess were determined by HPLC (an Agilent 1100 chromatograph (Agilent, Santa Clara, CA, USA), Chiralcel OD, 250 mm × 4.6 mm column).

The melting points were determined with an MPA 120 EZ-Melt automated melting point apparatus (Stanford Research Systems, Sunnyvale, CA, USA).

### 3.2. Syntheses

#### 3.2.1. Synthesis of Functionalized Amides **2**–**8** 

**Method A**. A solution of the corresponding acid chloride (1 equiv.) in dichloromethane (5–15 mL) was slowly added dropwise to a stirred solution of [amino(phenyl)methyl]diphenylphosphine sulfide **1a** (1 equiv.) or (aminomethyl)diphenylphosphine sulfide hydrochloride **1b** (1 equiv.) and triethylamine (1–2 (in the case of **1a**) or 2–3 (in the case of **1b**) equiv.) in CH_2_Cl_2_ (15–20 mL) at 5–10 °C. The reaction mixture was stirred at room temperature for 6 h (for ligands **3a** and **6b**) or 12 h (for the other cases) and was then sequentially washed with water, an aqueous solution of NaHCO_3_, and again with water. The organic layer was separated, dried over anhydrous Na_2_SO_4_, and evaporated to dryness. In the case of ligands **2**, **3a,** and **8**, the residue obtained was purified by column chromatography on silica gel (gradient elution with petroleum ether–CH_2_Cl_2_ from 7:1 to 1:3 (**2**), elution with neat CH_2_Cl_2_ (**3a**), or gradient elution with a petroleum ether–CH_2_Cl_2_ mixture from 2:1 to 1:3 and then with neat CH_2_Cl_2_ (**8**)) to yield the target products as light crystalline solids. In the other cases, the resulting residue was recrystallized from EtOAc (**3b**, **6a**) or EtOAc–hexane (1:1 (**6b**), 1:4 (**7a**), 1:2 (**7b**)) to provide the target ligands as light crystals.

**Method B**. A solution of (aminomethyl)diphenylphosphine sulfide hydrochloride **1b** (1 equiv.) and triethylamine (~2 equiv.) in CH_2_Cl_2_ (20 mL) was slowly added dropwise to a solution of the corresponding acid chloride (1 equiv.) in CH_2_Cl_2_ (20 mL) at 5–10 °C. The reaction mixture was stirred at room temperature for 10 h and then sequentially washed with water, an aqueous solution of NaHCO_3_, and again with water. The organic layer was separated, dried over anhydrous Na_2_SO_4_, and evaporated to dryness. The residue obtained was recrystallized from EtOAc to give the target ligand as a white crystalline solid.

**Method C**. A solution of [amino(phenyl)methyl]diphenylphosphine sulfide **1a** (1 equiv.) in CH_2_Cl_2_ (10 mL) was slowly added dropwise to a stirred suspension of benzo[*d*]thiazole-2-carboxylic acid (1 equiv.) and 4-dimethylaminopyridine (DMAP) (~0.1 equiv.) in CH_2_Cl_2_ (20 mL) at –5 to 0 °C under an argon atmosphere. The reaction mixture was stirred upon cooling for 20 min, and then a solution of *N*,*N′*-diisopropylcarbodiimide (1.3 equiv.) in CH_2_Cl_2_ (5 mL) was slowly added dropwise at −5 to 0 °C. The resulting mixture was stirred at room temperature for 30 min and left overnight. The precipitate obtained was filtered off, and the filtrate was evaporated to dryness. The resulting residue was purified by column chromatography on silica gel (eluent: CH_2_Cl_2_) to give the target ligand as a light yellow crystalline solid.

**4-Chloro-*N*-[(diphenylthiophosphoryl)(phenyl)methyl]picolinamide 2**


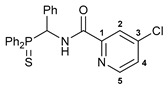


The compound was obtained by **method A** from amine **1a** (0.31 g, 0.96 mmol), 4-chloropicolinoyl chloride (0.17 g, 0.97 mmol), and Et_3_N (0.15 g, 1.48 mmol). Yield: 0.24 g (54%). Mp: 120–122 °C. ^31^P{^1^H} NMR (161.98 MHz): *δ* 51.81 ppm. ^1^H NMR (400.13 MHz, CDCl_3_): *δ* 6.45 (dd, 1H, CH, ^2^*J*_HP_ = 8.9 Hz, ^3^*J*_HH_ = 10.2 Hz), 7.12–7.23 (m, 5H, H_Ar_), 7.27–7.32 (m, 2H, H_Ar_), 7.40–7.44 (m, 2H, H_Ar_), 7.47–7.54 (m, 5H, H_Ar_), 8.05–8.10 (m, 3H, H_Ar_), 8.52 (d, 1H, H(C5), ^3^*J*_HH_ = 5.2 Hz), 9.47–9.50 (m, 1H, NH) ppm. ^13^C{^1^H} NMR (100.61 MHz, CDCl_3_): *δ* 51.72 (d, CH_2_, ^1^*J*_CP_ = 60.0 Hz), 122.93 and 126.64 (both s, C2 and C4), 127.79 (d, *m*-C in Ph, ^4^*J*_CP_ = 2.0 Hz), 128.18 (d, *p*-C in Ph, ^5^*J*_CP_ = 2.7 Hz), 128.19 (d, *m*-C in P(S)Ph, ^3^*J*_CP_ = 12.4 Hz), 128.63 (d, *o*-C in Ph, ^3^*J*_CP_ = 4.6 Hz), 128.89 (d, *m*-C in P(S)Ph, ^3^*J*_CP_ = 12.1 Hz), 129.88 (d, *ipso*-C in P(S)Ph, ^1^*J*_CP_ = 81.2 Hz), 130.60 (d, *ipso-*C in P(S)Ph, ^1^*J*_CP_ = 78.8 Hz), 131.62 (d, *o*-C in P(S)Ph, ^2^*J*_CP_ = 9.7 Hz), 131.87 (d, *p*-C in P(S)Ph, ^4^*J*_CP_ = 2.7 Hz), 132.03 (d, *o*-C in P(S)Ph, ^2^*J*_CP_ = 9.8 Hz), 132.06 (d, *p*-C in P(S)Ph, ^4^*J*_CP_ = 3.2 Hz), 134.08 (s, *ipso*-C in Ph), 145.68 (s, C3), 149.50 (s, C5), 150.47 (s, C1), 162.42 (d, C=O, ^3^*J*_CP_ = 7.2 Hz) ppm. IR (KBr, *ν*/cm^−1^): 476(w), 497(w), 534(m), 605(w) and 624(w) (both νP=S), 691(m), 698(m), 722(m), 756(w), 779(w), 801(w), 840(w), 907(vw), 998(vw), 1031(vw), 1103(m), 1179(w), 1234(w), 1292(w), 1308(w), 1350(w), 1395(w), 1437(m), 1462(w), 1495(m), 1510(br, s) (C(O)NH), 1555(w), 1579(w), 1670(s) (*ν*C=O), 2849(w), 2919(w), 3059(w), 3341(br, w) (νNH). Anal. Calcd for C_25_H_20_ClN_2_OPS: C, 64.86; H, 4.35; N, 6.05. Found: C, 64.37; H, 4.70; N, 5.82%.

***N*-[(Diphenylthiophosphoryl)(phenyl)methyl]quinoline-2-carboxamide 3a**


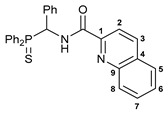


The compound was obtained by **method A** from amine **1a** (0.34 g, 1.05 mmol), quinoline-2-carbonyl chloride derived from quinaldic acid (0.18 g, 1.04 mmol), and an excess of SOCl_2_, and Et_3_N (0.21 g, 2.08 mmol). Yield: 0.31 g (62%). Mp: 238–240 °C. ^31^P{^1^H} NMR (161.98 MHz): *δ* 51.55 ppm. ^1^H NMR (400.13 MHz, CDCl_3_): *δ* 6.50–6.55 (m, 1H, CH), 7.14–7.22 (m, 3H, H_Ar_), 7.27–7.34 (m, 4H, H_Ar_), 7.42–7.51 (m, 4H, H_Ar_), 7.55–7.65 (m, 3H, H_Ar_), 7.78–7.82 (m, 1H, H_Ar_), 7.86 (d, 1H, H(C5) or H(C8), ^3^*J*_HH_ = 8.2 Hz), 8.07–8.13 (m, 2H, *o*-H in P(S)Ph), 8.19 (d, 1H, H(C2) or H(C3), ^3^*J*_HH_ = 8.5 Hz), 8.26 (d, 1H, HC(8) or H(C5), ^3^*J*_HH_ = 7.9 Hz), 8.27 (d, 1H, H(C3) or H(C2), ^3^*J*_HH_ = 8.5 Hz), 9.79 (dd, 1H, NH, ^3^*J*_HP_ = 5.4 Hz, ^3^*J*_HH_ = 10.1 Hz) ppm. ^13^C{^1^H} NMR (100.61 MHz, CDCl_3_): *δ* 51.98 (d, CH, ^1^*J*_CP_ = 59.8 Hz), 118.72 (s, C2), 127.55 (s, C5 or C6), 127.77 (d, *m*-C in Ph, ^4^*J*_CP_ = 2.0 Hz), 128.11 (d, *p*-C in Ph, ^5^*J*_CP_ = 3.0 Hz), 128.17 (s, C6 or C5), 128.21 (d, *m*-C in P(S)Ph, ^3^*J*_CP_ = 12.5 Hz), 128.71 (d, *o*-C in Ph, ^3^*J*_CP_ = 4.6 Hz), 128.85 (d, *m*-C in P(S)Ph, ^3^*J*_CP_ = 11.9 Hz), 129.44 (s, C4), 130.10 (s, C7 or C8), 130.17 (d, *ipso*-C in P(S)Ph, ^1^*J*_CP_ = 81.1 Hz), 130.43 (s, C8 and C7), 131.62 (d, *ipso*-C in P(S)Ph, ^1^*J*_CP_ = 83.7 Hz), 131.71 (d, *o*-C in P(S)Ph, ^2^*J*_CP_ = 9.5 Hz), 131.82 (d, *p*-C in P(S)Ph, ^4^*J*_CP_ = 2.8 Hz), 131.97 (d, *p*-C in P(S)Ph, ^4^*J*_CP_ = 2.6 Hz), 132.09 (d, *o*-C in P(S)Ph, ^2^*J*_CP_ = 9.5 Hz), 134.39 (s, *ipso*-C in Ph), 137.36 (s, C3), 146.58 and 148.79 (both s, C1 and C9), 163.80 (d, C=O, ^3^*J*_CP_ = 7.1 Hz) ppm. IR (KBr, *ν*/cm^−1^): 481(w), 492(w), 522(m), 527(m), 562(w), 608(m), and 632(w) (both *ν*P=S), 692(m), 699(m), 714(m), 725(m), 753(m), 773(m), 839(w), 917(vw), 998(vw), 1029(w), 1103(m), 1210(vw), 1343(w), 1380(w), 1428(m), 1438(m), 1495(br, s), 1525(br, m) (C(O)NH), 1566(w), 1673(s) (*ν*C=O), 2853(vw), 2925(vw), 2953(vw), 3057(w). Anal. Calcd for C_29_H_23_N_2_OPS: C, 72.79; H, 4.84; N, 5.85. Found: C, 73.37; H, 5.02; N, 5.83%.

***N*-[(Diphenylthiophosphoryl)methyl]quinoline-2-carboxamide 3b**


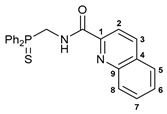


The compound was obtained by **method A** from amine hydrochloride **1b** (0.85 g, 3.00 mmol), quinoline-2-carbonyl chloride derived from quinaldic acid (0.52 g, 3.00 mmol), and an excess of SOCl_2_, and Et_3_N (1.3 mL, 9.33 mmol). Yield: 1.00 g (83%). Mp: 154–156 °C. ^31^P{^1^H} NMR (161.98 MHz, CDCl_3_): *δ* 41.80 ppm. ^1^H NMR (400.13 MHz, CDCl_3_): *δ* 4.62 (t, 2H, CH_2_, ^2^*J*_HP_ = 6.2 Hz), 7.47–7.56 (m, 6H, *m*-H and *p*-H in P(S)Ph_2_), 7.61–7.65 and 7.75–7.80 (both m, 1H + 1H, H(C6) and H(C7)), 7.87 (d, 1H, H(5) or H(C8), ^3^*J*_HH_ = 8.1 Hz), 7.94 (ddd, 4H, *o*-H in P(S)Ph_2_, ^3^*J*_HP_ = 13.0 Hz, ^3^*J*_HH_ = 7.5 Hz, ^4^*J*_HH_ = 1.6 Hz), 8.14 (d, 1H, H(C8) or H(C5), ^3^*J*_HH_ = 8.5 Hz), 8.20 and 8.29 (both d, 1H + 1H, H(C2) and H(C3), ^3^*J*_HH_ = 8.4 Hz), 9.02 (br. s, 1H, NH) ppm. ^13^C{^1^H} NMR (100.61 MHz, CDCl_3_): *δ* 41.84 (d, CH_2_, ^1^*J*_CP_ = 63.2 Hz), 118.74 (s, C2), 127.62 and 128.19 (both s, C5 and C6), 128.89 (d, *m*-C in P(S)Ph_2_, ^3^*J*_CP_ = 12.5 Hz), 129.41 (s, C4), 130.12 and 130.21 (both s, C7 and C8), 130.64 (d, *ipso*-C in P(S)Ph_2_, ^1^*J*_CP_ = 80.0 Hz), 131.49 (d, *o*-C in P(S)Ph_2_, ^2^*J*_CP_ = 10.3 Hz), 132.13 (d, *p*-C in P(S)Ph_2_, ^2^*J*_CP_ = 2.8 Hz), 137.49 (s, C3), 146.49 and 148.73 (both s, C1 and C9), 164.29 (d, C=O, ^3^*J*_CP_ = 4.7 Hz) ppm. IR (KBr, *ν*/cm^−1^): 481(w), 504(w), 521(w), 611(m) and 620(w) (both *ν*P=S), 692(m), 709(m), 740(m), 774(m), 795(vw), 845(w), 862(w), 924(w), 999(vw), 1106(m), 1171(w), 1212(w), 1309(vw), 1378(w), 1428(m), 1437(m), 1500(s), 1522(br, s) (C(O)NH), 1566(w), 1592(w), 1618(w), 1678(br, s) (*ν*C=O), 2853(vw), 2923(w), 2962(vw), 3055(w). Anal. Calcd for C_23_H_19_N_2_OPS: C, 68.64; H, 4.76; N, 6.96. Found: C, 68.49; H, 4.74; N, 6.91%.

***N*-[(Diphenylthiophosphoryl)methyl]quinoline-8-carboxamide 4**


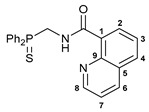


The compound was obtained by **method B** from amine hydrochloride **1b** (0.57 g, 2.01 mmol), quinoline-8-carbonyl chloride derived from quinoline-8-carboxylic acid (0.35 g, 2.02 mmol), and an excess of SOCl_2_, and Et_3_N (0.6 mL, 4.30 mmol). Yield: 0.60 g (71%). Mp: 199–201 °C. ^31^P{^1^H} NMR (161.98 MHz, CDCl_3_): *δ* 41.65 ppm. ^1^H NMR (400.13 MHz, CDCl_3_): *δ* 4.80 (dd, 2H, CH_2_, ^2^*J*_HP_ = ^3^*J*_HH_ = 5.9 Hz), 7.45–7.54 (m, 7H, H_Ar_), 7.64–7.68 (m, 1H, H_Ar_), 7.94–7.99 (m, 5H, H_Ar_), 8.26 (d, 1H, H_Ar_, ^3^*J*_HH_ = 8.2 Hz), 8.79 (d, 1H, H_Ar_, ^3^*J*_HH_ = 7.3 Hz), 8.86 (dd, 1H, H(C8), ^3^*J*_HH_ = 4.0 Hz, ^4^*J*_HH_ = 1.6 Hz), 12.34 (br. s, 1H, NH) ppm. ^13^C{^1^H} NMR (100.61 MHz, CDCl_3_): *δ* 43.08 (d, CH_2_, ^1^*J*_CP_ = 64.1 Hz), 120.89 (s, C7), 126.22 (s, C3), 127.69 (d, C1, ^4^*J*_CP_ = 4.3 Hz), 128.21 (s, C5), 128.59 (d, *m*-C in P(S)Ph_2_, ^3^*J*_CP_ = 11.7 Hz), 131.03 (d, *ipso*-C in P(S)Ph_2_, ^1^*J*_CP_ = 88.0 Hz), 131.42 (d, *o*-C in P(S)Ph_2_, ^2^*J*_CP_ = 10.3 Hz), 131.72 (d, *p*-C in P(S)Ph_2_, ^4^*J*_CP_ = 2.6 Hz), 132.10 and 133.71 (both s, C2 and C4), 137.45 (s, C6), 145.23 (s, C9), 149.33 (s, C8), 165.73 (d, C=O, ^3^*J*_CP_ = 5.9 Hz) ppm. IR (KBr, *ν*/cm^−1^): 484(w), 509(w), 519(m), 610(m), 620(w), and 648(m) (three *ν*P=S), 690(m), 709(m), 733(m), 757(m), 794(m), 839(vw), 919(w), 998(vw), 1014(vw), 1028(vw), 1050(vw), 1103(m), 1158(w), 1194(w), 1205(w), 1255(w), 1287(w), 1388(w), 1420(w), 1437(m), 1463(w), 1490(w), 1499(m), 1535(br, m) (C(O)NH), 1575(m), 1592(m), 1609(w), 1652(s) (*ν*C=O), 2889(w), 2965(w), 3058(w), 3164(br, w) (*ν*NH). Anal. Calcd for C_23_H_19_N_2_OPS·H_2_O: C, 65.70; H, 5.03; N, 6.66. Found: C, 65.63; H, 4.67; N, 6.66%.

***N*-[(Diphenylthiophosphoryl)(phenyl)methyl]benzo[*d*]thiazole-2-carboxamide 5**


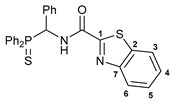


The compound was obtained by **method C** from amine **1a** (0.52 g, 1.61 mmol), benzo[*d*]thiazole-2-carboxylic acid (0.29 g, 1.62 mmol), DMAP (16 mg, 0.13 mmol), and *N*,*N′*-diisopropylcarbodiimide 0.27 g (2.14 mmol). Yield: 0.62 g (80%). Mp: 220–225 °C. ^31^P{^1^H} NMR (161.98 MHz, CDCl_3_): *δ* 51.65 ppm. ^1^H NMR (400.13 MHz, CDCl_3_): *δ* 6.43–6.48 (m, 1H, CH), 7.15–7.23 (m, 3H, H_Ar_), 7.26–7.33 (m, 4H, H_Ar_), 7.42–7.59 (m, 8H, H_Ar_), 7.94 (d, 1H, H(C3) or H(C6), ^3^*J*_HH_ = 8.0 Hz), 8.08–8.13 (m, 2H, *o*-H in P(S)Ph), 8.17 (d, 1H, H(C6) or H(C3), ^3^*J*_HH_ = 8.3 Hz), 8.95 (dd, 1H, NH, ^2^*J*_HP_ = 5.4 Hz, ^3^*J*_HH_ = 9.8 Hz) ppm. ^13^C{^1^H} NMR (100.61 MHz, CDCl_3_): *δ* 51.84 (d, CH, ^1^*J*_CP_ = 59.7 Hz), 122.24 and 124.92 (both s, C3 and C6), 126.91 and 126.96 (both s, C4 and C5), 127.91 (d, *m*-C in Ph, ^4^*J*_CP_ = 1.9 Hz), 128.27 (d, *m*-C in P(S)Ph, ^3^*J*_CP_ = 12.5 Hz), 128.36 (d, *p*-C in Ph, ^5^*J*_CP_ = 3.3 Hz), 128.69 (d, *o*-C in Ph, ^3^*J*_CP_ = 4.4 Hz), 129.03 (d, *m*-C in P(S)Ph, ^3^*J*_CP_ = 11.7 Hz), 129.62 (d, *ipso*-C in P(S)Ph, ^1^*J*_CP_ = 81.7 Hz), 130.35 (d, *ipso*-C in P(S)Ph, ^1^*J*_CP_ = 79.3 Hz), 131.64 (d, *o*-C in P(S)Ph, ^2^*J*_CP_ = 9.5 Hz), 132.01 (d, *p*-C in P(S)Ph, ^4^*J*_CP_ = 3.1 Hz), 132.07 (d, *o*-C in P(S)Ph, ^2^*J*_CP_ = 9.5 Hz), 132.23 (d, *p*-C in P(S)Ph, ^4^*J*_CP_ = 2.8 Hz), 133.79 (d, *ipso*-C in Ph), 137.11 (s, C2), 152.89 (s, C7), 159.24 (d, C=O, ^3^*J*_CP_ = 7.7 Hz), 162.11 (s, C1) ppm. IR (KBr, *ν*/cm^−1^): 482(w), 522(m), 538(w), 582(vw), 607(m) and 626(w) (both *ν*P=S), 691(m), 698(m), 717(m), 728(m), 751(m), 767(w), 831(vw), 892(w), 918(vw), 998(vw), 1029(vw), 1103(m), 1142(w), 1249(vw), 1290(w), 1318(w), 1351(w), 1437(m), 1455(w), 1513(br, s) (C(O)NH), 1556(w), 1585(vw), 1600(vw), 1675(m) (*ν*C=O), 2946(vw), 3058(w), 3295(br, w) (*ν*NH). Anal. Calcd for C_27_H_21_N_2_OPS_2_: C, 66.92; H, 4.37; N, 5.78. Found: C, 67.29; H, 4.61; N, 5.33%.

***N*-[(Diphenylthiophosphoryl)(phenyl)methyl]thiophene-2-carboxamide 6a**


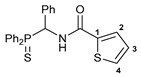


The compound was obtained by **method A** from amine **1a** (0.31 g, 0.96 mmol), thiophene-2-carbonyl chloride (0.14 g, 0.96 mmol), and Et_3_N (0.11 g, 1.09 mmol). Yield: 0.33 g (80%). Mp: 206–208 °C. ^31^P{^1^H} NMR (161.98 MHz, CDCl_3_): *δ* 52.64 ppm. ^1^H NMR (400.13 MHz, CDCl_3_): *δ* 6.35–6.40 (m, 1H, CH), 7.08 (dd, 1H, H(C3), ^3^*J*_HH_ = 4.8 Hz, ^3^*J*_HH_ = 3.8 Hz), 7.11–7.22 (m, 5H, H_Ar_), 7.25–7.30 (m, 2H, H_Ar_), 7.39–7.58 (m, 8H, H_Ar_), 7.69–7.73 (br. m, 1H, NH), 8.03–8.08 (m, 2H, *o*-H in P(S)Ph) ppm. ^13^C{^1^H} NMR (100.61 MHz, CDCl_3_): *δ* 51.15 (d, CH_2_, ^1^*J*_CP_ = 58.8 Hz), 127.74 (s, C2 or C3), 127.86 (d, *m*-C in Ph, ^4^*J*_CP_ = 1.9 Hz), 128.18 (d, *p*-C in Ph, ^5^*J*_CP_ = 2.8 Hz), 128.19 (d, *m*-C in P(S)Ph, ^3^*J*_CP_ = 12.5 Hz), 128.46 (d, *o*-C in Ph, ^3^*J*_CP_ = 4.6 Hz), 128.58 (s, C3 or C2), 129.06 (d, *m*-C in P(S)Ph, ^3^*J*_CP_ = 11.7 Hz), 129.38 (d, *ipso*-C in P(S)Ph, ^1^*J*_CP_ = 82.4 Hz), 130.64 (d, *ipso*-C in P(S)Ph, ^1^*J*_CP_ = 79.2 Hz), 130.84 (s, C4), 131.49 (d, *o*-C in P(S)Ph, ^2^*J*_CP_ = 9.5 Hz), 131.97 (d, *p*-C in P(S)Ph, ^4^*J*_CP_ = 3.1 Hz), 132.06 (d, *o*-C in P(S)Ph, ^2^*J*_CP_ = 10.3 Hz), 132.20 (d, *p*-C in P(S)Ph, ^4^*J*_CP_ = 2.9 Hz), 134.27 (s, *ipso*-C in Ph), 137.97 (s, C1), 160.77 (d, C=O, ^3^*J*_CP_ = 8.0 Hz) ppm. IR (KBr, *ν*/cm^−1^): 482(w), 525(m), 551(vw), 604(w) and 625(w) (both *ν*P=S), 696(m), 722(s), 739(w), 744(w), 752(w), 856(w), 1099(m), 1261(vw), 1291(w), 1307(w), 1355(w), 1418(w), 1437(m), 1500(s), 1504(s), 1527(br, s) (C(O)NH), 1654(s) (*ν*C=O), 2951(vw), 3056(w), 3112(vw), 3334(br, w) (*ν*NH). Anal. Calcd for C_24_H_20_NOPS_2_: C, 66.49; H, 4.65; N, 3.23. Found: C, 66.41; H, 4.57; N, 3.31%.

***N*-[(Diphenylthiophosphoryl)methyl]thiophene-2-carboxamide 6b**


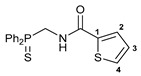


The compound was obtained by **method A** from amine hydrochloride **1b** (0.29 g, 1.02 mmol), thiophene-2-carbonyl chloride (0.15 g, 1.02 mmol), and Et_3_N (0.22 g, 2.17 mmol). Yield: 0.31 g (85%). Mp: 147–149 °C. ^31^P{^1^H} NMR (161.98 MHz, CDCl_3_): *δ* 42.41 ppm. ^1^H NMR (400.13 MHz, CDCl_3_): *δ* 4.47 (dd, 2H, CH_2_, ^2^*J*_HP_ = ^3^*J*_HH_ = 5.8 Hz), 6.93 (br. s, 1H, NH), 7.07–7.09 (m, 1H, H(C3)), 7.48–7.58 (m, 8H, H_Ar_), 7.86–7.91 (m, 4H, *o*-H in P(S)Ph_2_) ppm. ^13^C{^1^H} NMR (100.61 MHz, CDCl_3_): *δ* 40.87 (d, CH_2_, ^1^*J*_CP_ = 62.8 Hz), 127.76 and 128.53 (both s, C2 and C3), 128.98 (d, *m*-C in P(S)Ph_2_, ^3^*J*_CP_ = 11.7 Hz), 130.33 (d, *ipso*-C in P(S)Ph_2_, ^1^*J*_CP_ = 80.9 Hz), 130.73 (s, C4), 131.30 (d, *o*-C in P(S)Ph_2_, ^2^*J*_CP_ = 10.3 Hz), 132.29 (d, *p*-C in P(S)Ph_2_, ^4^*J*_CP_ = 3.0 Hz), 137.88 (s, C1), 161.49 (d, C=O, ^3^*J*_CP_ = 5.7 Hz) ppm. IR (KBr, *ν*/cm^−1^): 494(w), 519(w), 574(w), 606(m) and 618(vw) (both *ν*P=S), 692(m), 704(m), 712(m), 725(m), 738(m), 750(w), 767(w), 858(w), 901(m), 998(vw), 1107(m), 1237(w), 1267(m), 1300(m), 1352(w), 1395(w), 1416(w), 1435(m), 1505(s), 1528(br, s) (C(O)NH), 1650 (*ν*C=O), 2921(vw), 2976(w), 3059(vw), 3104(vw), 3351(br, w) (*ν*NH). Anal. Calcd for C_18_H_16_NOPS_2_: C, 60.49; H, 4.51; N, 3.92. Found: C, 61.04; H, 4.41; N, 3.99%.

***N*-[(Diphenylthiophosphoryl)(phenyl)methyl]-2-(methylthio)acetamide 7a**


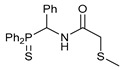


The compound was obtained by **method A** from amine **1a** (0.34 g, 1.05 mmol), 2-(methylthio)acetyl chloride (0.13 g, 1.04 mmol), and Et_3_N (0.11 g, 1.09 mmol). Yield: 0.37 g (86%). Mp: 195–197 °C. ^31^P{^1^H} NMR (161.98 MHz, CDCl_3_): *δ* 52.30 ppm. ^1^H NMR (400.13 MHz, CDCl_3_): *δ* 1.79 (s, 3H, Me), 3.09 and 3.17 (ABq, 2H, CH_2_S, *J*_AB_ = 16.5 Hz), 6.28–6.33 (m, 1H, CH), 7.15–7.29 (m, 7H, H_Ar_), 7.38–7.49 (m, 3H, H_Ar_), 7.54–7.62 (m, 3H, H_Ar_), 8.09–8.14 (m, 2H, *o*-H in P(S)Ph), 8.41–8.44 (m, 1H, NH) ppm. IR (KBr, *ν*/cm^−1^): 481(w), 518(m), 534(m), 548(w), 592(w), 625(w) (*ν*P=S), 693(m), 719(m), 738(w), 804(w), 1030(vw), 1106(br, m), 1133(w), 1179(vw), 1291(vw), 1313(w), 1437(m), 1455(w), 1495(m), 1503(br, s) (C(O)NH), 1669(s) (*ν*C=O), 2914(vw), 2946(vw), 3058(w), 3331(w) (*ν*NH). Anal. Calcd for C_22_H_22_NOPS_2_: C, 64.21; H, 5.39; N, 3.40. Found: C, 64.18; H, 5.36; N, 3.41%.

***N*-[(Diphenylthiophosphoryl)methyl]-2-(methylthio)acetamide 7b**


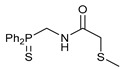


The compound was obtained by **method A** from amine hydrochloride **1b** (0.34 g, 1.20 mmol), 2-(methylthio)acetyl chloride (0.15 g, 1.20 mmol), and Et_3_N (0.25 g, 2.47 mmol). Yield: 0.30 g (75%). Mp: 82–84 °C. ^31^P{^1^H} NMR (161.98 MHz, CDCl_3_): *δ* 42.08 ppm. ^1^H NMR (400.13 MHz, CDCl_3_): *δ* 1.89 (s, 3H, Me), 3.14 (s, 2H, CH_2_S), 4.38 (dd, 2H, CH_2_P, ^2^*J*_HP_ = ^3^*J*_HH_ = 5.8 Hz), 7.48–7.58 (m, 6H, *m*-H and *p*-H in P(S)Ph_2_), 7.65 (br. s, 1H, NH), 7.89 (ddd, *o*-H in P(S)Ph_2_, ^3^*J*_HP_ = 13.0 Hz, ^3^*J*_HH_ = 7.6 Hz, ^4^*J*_HH_ = 1.2 Hz) ppm. IR (KBr, *ν*/cm^−1^): 487(w), 509(w), 566(w), 597(m), 642(w) (*ν*P=S), 691(m), 704(m), 713(m), 744(m), 776(w), 824(m), 962(w), 1000(w), 1107(m), 1170(vw), 1237(w), 1258(w), 1386(m), 1437(m), 1479(w), 1516(br, s) (C(O)NH), 1660(s) (*ν*C=O), 2912(w), 2960(vw), 3051(vw), 3329(m) (*ν*NH). Anal. Calcd for C_16_H_18_NOPS_2_: C, 57.29; H, 5.41; N, 4.18. Found: C, 57.41; H, 5.37; N, 4.23%.

***N*-[(Diphenylthiophosphoryl)(phenyl)methyl]-2-(methylthio)benzamide 8**


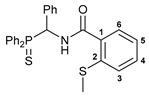


The compound was obtained by **method A** from a hydrochloride salt of amine **1a** (0.25 g, 0.69 mmol), 2-(methylthio)benzoyl chloride (0.13 g, 0.70 mmol), and Et_3_N (0.18 g, 1.78 mmol). Yield: 0.15 g (44%). Mp: 140–145 °C. ^31^P{^1^H} NMR (202.45 MHz, CDCl_3_): *δ* 52.32 ppm. ^1^H NMR (500.13 MHz, CDCl_3_): *δ* 2.33 (s, 3H, Me), 6.55 (dd, 1H, CH, ^2^*J*_HP_ = ^3^*J*_HH_ = 9.3 Hz), 7.14–7.17 (m, 3H, H(C5) + *m*-H in Ph), 7.20–7.23 (m, 3H, *o*-H and *p*-H in Ph), 7.26–7.31 (m, 3H, *m*-H in P(S)Ph + H(C4)), 7.36–7.43 (m, 3H, H(C3) + H(C6) + *p-*H in P(S)Ph), 7.46–7.50 (m, 2H, *o*-H in P(S)Ph), 7.54–7.60 (m, 3H, *m*-H and *p*-H in P(S)Ph), 7.95–7.98 (m, 1H, NH), 8.13–8.18 (m, 2H, *o*-H in P(S)Ph) ppm. ^13^C{^1^H} NMR (125.76 MHz, CDCl_3_): *δ* 16.97 (s, Me), 51.90 (d, CH, ^1^*J*_CP_*=* 59.2 Hz), 125.27 (s, C5), 127.69 (s, C4), 127.73 (d, *m*-C in Ph, ^4^*J*_CP_ = 2.2 Hz), 128.11 (d, *p-*C in Ph, ^5^*J*_CP_ = 3.0 Hz), 128.18 (d, *m-*C in P(S)Ph, ^3^*J*_CP_ = 12.7 Hz), 128.48 (s, C6), 128.75 (d, *o*-C in Ph, ^3^*J*_CP_ = 4.7 Hz), 128.98 (d, *m*-C in P(S)Ph, ^3^*J*_CP_ = 12.0 Hz), 129.82 (d, *ipso*-C in P(S)Ph, ^1^*J*_CP_ = 80.9 Hz), 130.51 (d, *ipso*-C in P(S)Ph, ^1^*J*_CP_ = 79.3 Hz), 131.09 (s, C3), 131.82 (d, *o*-C in P(S)Ph, ^2^*J*_CP_ = 9.8 Hz), 131.88 (d, *p*-C in P(S)Ph, ^4^*J*_CP_ = 3.1 Hz), 131.97 (d, *o*-C in P(S)Ph, ^2^*J*_CP_ = 10.2 Hz), 132.11 (d, *p*-C in P(S)Ph, ^4^*J*_CP_ = 2.9 Hz), 133.96 (s, C1), 133.98 (d, *ipso*-C in Ph, ^2^*J*_CP_ = 1.2 Hz), 137.83 (s, C6), 166.96 (d, C=O, ^3^*J*_CP_ = 7.2 Hz) ppm. IR (KBr, *ν*/cm^−1^): 479(w), 527(s), 605(w) and 628(m) (both *ν*P=S), 652(w), 695(s), 714(m), 747(m), 787(w), 894(vw), 999(vw), 1028(vw), 1102(m), 1149(vw), 1185(vw), 1247(w), 1286(w), 1309(m), 1345(w), 1386(w), 1436(s), 1461(m), 1493(br, s) (C(O)NH), 1586(m), 1656(br, s) (*ν*C=O), 2852(vw), 2922(w), 3056(w), 3319(br, w) (*ν*NH). Anal. Calc. for C_27_H_24_NOPS_2_·0.15CH_2_Cl_2_: C, 67.05; H, 5.04; N, 2.88. Found: C, 67.09; H, 5.17; N, 2.94%.

#### 3.2.2. Synthesis of Pd(II) Pincer Complexes **9**–**14** 

**General procedure**. A solution of PdCl_2_(NCPh)_2_ (61 mg, 0.159 mmol) in CH_2_Cl_2_ (4 mL) was slowly added dropwise to a solution of the corresponding ligand (0.159 mmol) and Et_3_N (23 μL, 0.165 mmol) in CH_2_Cl_2_ (6 mL). The reaction mixture was left under ambient conditions for 2 h (in the case of ligand **7a**) or 1 day (in the other cases). After the mentioned period of time, the resulting mixture was purified by column chromatography on silica gel (eluent: CH_2_Cl_2_–MeOH (50:1 (**9**), 100:1 (**14**), 200:1 (**12**)), CH_2_Cl_2_–EtOH (50:1 (**10a**), 20:1 (**10b**)), CHCl_3_–EtOH (20:1 (**11**), 50:1 (**13a**,**b**)) to provide the target complexes as beige (**9**), yellow (**11**, **12**, **13a**, **14**), or orange (**10a**,**b**, **13b**) crystalline solids.

**[κ^3^-*S*,*N*,*N*-(L)Pd(II)Cl] complex 9**


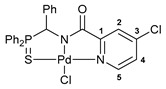


Yield: 70 mg (73%). ^31^P{^1^H} NMR (161.98 MHz, CDCl_3_): *δ* 67.75 ppm. ^1^H NMR (500.13 MHz, CDCl_3_): *δ* 6.48 (d, 1H, CH, ^2^*J*_HP_ = 2.7 Hz), 7.13 (dd, 2H, *o*-H in P(S)Ph, ^3^*J*_HP_ = 12.8 Hz, ^3^*J*_HH_ = 7.8 Hz), 7.17–7.20 (m, 2H, *m*-H in Ph), 7.23–7.28 (m, 3H, *m*-H in P(S)Ph + *p*-H in Ph), 7.48–7.51 (m, 2H, H(C4) + *p*-H in P(S)Ph), 7.58–7.60 (m, 2H, *o*-H in Ph), 7.66–7.70 (m, 2H, *m*-H in P(S)Ph), 7.72–7.76 (m, 1H, *p*-H in P(S)Ph), 7.81 (d, 1H, H(C2), ^4^*J*_HH_ = 2.4 Hz), 8.14 (dd, 2H, *o*-H in P(S)Ph, ^3^*J*_HP_ = 12.8 Hz, ^3^*J*_HH_ = 7.5 Hz), 8.97 (d, 1H, H(C5), ^3^*J*_HH_ = 5.9 Hz) ppm. ^13^C{^1^H} NMR (125.76 MHz, CDCl_3_–(CD_3_)_2_SO): *δ* 65.06 (d, CH, ^1^*J*_CP_ = 71.6 Hz), 124.81 (d, *ipso*-C in P(S)Ph, ^1^*J*_CP_ = 75.1 Hz), 126.00 (s, C2), 126.20 (d, *ipso*-C in P(S)Ph, ^1^*J*_CP_ = 78.1 Hz), 127.33 (s, C4), 128.12 (d, *o*-C in Ph, ^3^*J*_CP_ = 5.3 Hz), 128.15 (d, *m*-C in Ph, ^4^*J*_CP_ = 3.1 Hz), 128.65 (d, *m*-C in P(S)Ph, ^3^*J*_CP_ = 12.5 Hz), 128.84 (d, *p*-C in Ph, ^5^*J*_CP_ = 3.6 Hz), 129.85 (d, *m*-C in P(S)Ph, ^3^*J*_CP_ = 12.3 Hz), 132.50 (d, *o*-C in P(S)Ph, ^2^*J*_CP_ = 9.4 Hz), 132.75 (d, *o*-C in P(S)Ph, ^2^*J*_CP_ = 9.9 Hz), 133.58 (d, *ipso*-C in Ph, ^2^*J*_CP_ = 1.2 Hz), 133.62 (d, *p*-C in P(S)Ph, ^4^*J*_CP_ = 2.6 Hz), 133.64 (d, *p*-C in P(S)Ph, ^4^*J*_CP_ = 2.2 Hz), 148.15 (s, C5), 148.28 (s, C3), 154.78 (s, C1), 169.76 (d, C=O, ^3^*J*_CP_ = 14.6 Hz) ppm. IR (KBr, *ν*/cm^−1^): 488(w), 500(w), 525(w), 536(m), 575(m) and 583(w) (both *ν*P=S), 618(vw), 692(m), 701(m), 710(m), 719(m), 732(w), 753(w), 778(w), 806(w), 841(w), 886(vw), 998(w), 1029(w), 1059(br, w), 1104(m), 1112(m), 1192(w), 1251(vw), 1340(m), 1355(m), 1421(w), 1437(m), 1454(w), 1492(w), 1507(w), 1553(w), 1594(s), 1618(s) (*ν*C=O), 2892(vw), 3023(vw), 3058(vw). Anal. Calcd for C_25_H_19_Cl_2_N_2_OPPdS: C, 49.73; H, 3.17; N, 4.64. Found: C, 49.54; H, 3.21; N, 4.61%.

**[κ^3^-*S*,*N*,*N*-(L)Pd(II)Cl] complex 10a**


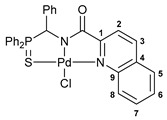


Yield: 81 mg (82%). ^31^P{^1^H} NMR (161.98 MHz, CDCl_3_): *δ* 66.26 ppm. ^1^H NMR (400.13 MHz, CDCl_3_): *δ* 6.73 (d, 1H, CH, ^2^*J*_HP_ = 3.2 Hz), 7.13–7.31 (m, 7H, H_Ar_), 7.49–7.53 (m, 1H, H_Ar_), 7.65–7.75 (m, 6H, H_Ar_), 7.84–7.90 (m, 2H, H_Ar_), 8.02 (d, 1H, H_Ar_, ^3^*J*_HH_ = 8.2 Hz), 8.19–8.24 (m, 2H, *o*-H in P(S)Ph), 8.41 (d, 1H, H_Ar_, ^3^*J*_HH_ = 8.2 Hz), 9.86 (d, 1H, H_Ar_, ^3^*J*_HH_ = 8.9 Hz) ppm. ^13^C{^1^H} NMR (100.61 MHz, CDCl_3_–(CD_3_)_2_SO): *δ* 64.41 (d, CH, ^1^*J*_CP_ = 68.5 Hz), 121.74 (s, C2), 123.97 (d, *ipso*-C in P(S)Ph, ^1^*J*_CP_ = 73.9 Hz), 126.09 (d, *ipso*-C in P(S)Ph, ^1^*J*_CP_ = 78.3 Hz), 128.01 (d, *m*-C in Ph, ^4^*J*_CP_ = 2.7 Hz), 128.26, 128.31, and 128.32 (three s, C5–C8), 128.70 (d, *p*-C in Ph, ^5^*J*_CP_ = 3.1 Hz), 128.85 (d, *m*-C in P(S)Ph, ^3^*J*_CP_ = 11.7 Hz), 128.89 (d, *o*-C in Ph, ^3^*J*_CP_ = 3.9 Hz), 129.85 (d, *m*-C in P(S)Ph, ^3^*J*_CP_ = 12.4 Hz), 131.22 (s, C4), 131.40 (s, C5–C8), 132.67 (d, *o*-C in P(S)Ph, ^2^*J*_CP_ = 9.8 Hz), 132.87 (d, *o*-C in P(S)Ph, ^2^*J*_CP_ = 10.0 Hz), 133.84 (d, *p*-C in P(S)Ph, ^4^*J*_CP_ = 2.6 Hz), 133.95 (d, *p*-C in P(S)Ph, ^4^*J*_CP_ = 2.8 Hz), 134.23 (s, *ipso*-C in Ph), 141.30 (s, C3), 146.74 and 155.46 (both s, C1 and C9), 171.30 (d, C=O, ^3^*J*_CP_ = 13.1 Hz) ppm. IR (KBr, *ν*/cm^−1^): 495(w), 526(m), 579(m) (*ν*P=S), 606(w), 689(m), 696(m), 708(m), 750(w), 764(m), 848(w), 929(vw), 998(w), 1028(w), 1104(m), 1155(w), 1187(w), 1214(vw), 1342(w), 1375(m), 1385(m), 1437(m), 1461(w), 1491(w), 1514(w), 1560(w), 1618(vs) (*ν*C=O), 2916(w), 3057(w). Anal. Calcd for C_29_H_22_ClN_2_OPPdS: C, 56.23; H, 3.58; N, 4.52. Found: C, 56.31; H, 3.65; N, 4.56%.

**[κ^3^-*S*,*N*,*N*-(L)Pd(II)Cl] complex 10b**


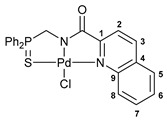


Yield: 56 mg (65%). ^31^P{^1^H} NMR (161.98 MHz, CDCl_3_): *δ* 59.24 ppm. ^1^H NMR (400.13 MHz, CDCl_3_): *δ* 4.90 (d, 2H, CH_2_P, ^2^*J*_HP_ = 4.7 Hz), 7.56–7.69 (m, 7H, H_Ar_), 7.80–7.92 (m, 6H, H_Ar_), 8.01 (d, 1H, H_Ar_, ^3^*J*_HH_ = 8.3 Hz), 8.40 (d, 1H, H_Ar_, ^3^*J*_HH_ = 8.3 Hz), 9.72 (d, 1H, H_Ar_, ^3^*J*_HH_ = 9.1 Hz) ppm. ^13^C{^1^H} NMR (100.61 MHz, CDCl_3_–(CD_3_)_2_SO): *δ* 54.04 (d, CH_2_P, ^1^*J*_CP_ = 74.2 Hz), 121.35 (s, C2), 124.34 (d, *ipso*-C in P(S)Ph_2_, ^1^*J*_CP_ = 77.8 Hz), 127.76, 128.57, and 128.86 (three s, C5–C8), 129.56 (d, *m*-C in P(S)Ph_2_, ^3^*J*_CP_ = 12.7 Hz), 130.82 (s, C4), 131.23 (s, C5–C8), 131.99 (d, *o*-C in P(S)Ph_2_, ^2^*J*_CP_ = 10.6 Hz), 133.86 (d, *p*-C in P(S)Ph_2_, ^4^*J*_CP_ = 2.9 Hz), 140.67 (s, C3), 146.68 and 155.31 (both s, C1 and 9) ppm (the signal of C=O carbon nucleus was not observed). IR (KBr, *ν*/cm^−1^): 478(w), 506(m), 528(w), 557(w), 584(m) (*ν*P=S), 688(m), 702(m), 717(m), 747(w), 764(m), 848(w), 929(w), 998(w), 1028(vw), 1107(m), 1150(m), 1188(vw), 1209(vw), 1393(br, m), 1437(m), 1463(m), 1485(w), 1514(m), 1560(w), 1597(m), 1628(vs) (*ν*C=O), 2874(vw), 2924(vw), 3053(w). Anal. Calcd for C_23_H_18_ClN_2_OPPdS: C, 50.85; H, 3.34; N, 5.16. Found: C, 50.81; H, 3.51; N, 5.27%.

**[κ^3^-*S*,*N*,*N*-(L)Pd(II)Cl] complex 11**


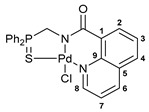


Yield: 74 mg (86%). ^31^P{^1^H} NMR (161.98 MHz, CDCl_3_): *δ* 53.01 ppm. ^1^H NMR (400.13 MHz, CDCl_3_): *δ* 5.44 (d, 2H, CH_2_, ^2^*J*_HP_ = 4.4 Hz), 7.47 (dd, 1H, H(C7), ^3^*J*_HH_ = 8.0 Hz, ^3^*J*_HH_ = 5.4 Hz), 7.57–7.67 (m, 7H, H_Ar_), 7.91 (d, 1H, H_Ar_, ^3^*J*_HH_ = 8.0 Hz), 7.97 (dd, 4H, *o*-H in P(S)Ph_2_, ^3^*J*_HP_ = 13.3 Hz, ^3^*J*_HH_ = 7.3 Hz), 8.37 (d, 1H, H_Ar_, ^3^*J*_HH_ = 8.1 Hz), 8.84 (d, 1H, H_Ar_, ^3^*J*_HH_ = 7.4 Hz), 9.67 (d, 1H, H(C8), ^3^*J*_HH_ = 5.4 Hz) ppm. ^13^C{^1^H} NMR (100.61 MHz, CDCl_3_): *δ* 61.54 (d, CH_2_, ^1^*J*_CP_ = 69.0 Hz), 121.19 (s, C7), 124.24 (d, *ipso*-C in P(S)Ph_2_, ^1^*J*_CP_ = 77.7 Hz), 127.36 (s, C3), 128.89 (s, C5), 129.39 (d, *m*-C in P(S)Ph_2_, ^3^*J*_CP_ = 12.5 Hz), 131.29 (s, C2, C4, or C6), 131.50 (s, C1), 132.63 (d, *o*-C in P(S)Ph_2_, ^2^*J*_CP_ = 10.9 Hz), 133.54 (d, *p*-C in P(S)Ph_2_, ^4^*J*_CP_ = 2.7 Hz), 136.73 (s, C2, C4, or C6), 140.41 (s, C2, C4, or C6), 143.40 (s, C9), 155.69 (s, C8), 163.72 (d, C=O, ^3^*J*_CP_ = 6.6 Hz) ppm. IR (KBr, *ν*/cm^−1^): 492(w), 520(m), 583(m) (*ν*P=S), 694(m), 718(m), 746(w), 756(w), 784(m), 838(w), 933(w), 998(vw), 1111(m), 1179(m), 1308(w), 1374(m), 1395(w), 1438(m), 1506(w), 1560(s), 1581(m), 1614(m) (*ν*C=O), 2953(w), 3052(vw). Anal. Calcd for C_23_H_18_ClN_2_OPPdS: C, 50.85; H, 3.34; N, 5.16. Found: C, 50.97; H, 3.34; N, 5.14%.

**[κ^3^-*S*,*N*,*N*-(L)Pd(II)Cl] complex 12**


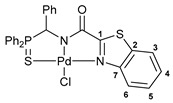


Yield: 75 mg (75%). ^31^P{^1^H} NMR (161.98 MHz, CDCl_3_): *δ* 67.28 ppm. ^1^H NMR (400.13 MHz, CDCl_3_): *δ* 6.48 (d, 1H, CH, ^2^*J*_HP_ = 2.4 Hz), 7.13 (dd, 2H, *o*-H in P(S)Ph, ^3^*J*_HP_ = 12.8 Hz, ^3^*J*_HH_ = 7.4 Hz), 7.19–7.23 (m, 2H, H_Ar_), 7.25–7.30 (m, 3H, H_Ar_), 7.49–7.56 (m, 2H, H_Ar_), 7.60–7.78 (m, 6H, H_Ar_), 7.91 (d, 1H, H(C3) or H(C6), ^3^*J*_HH_ = 8.0 Hz), 8.16–8.21 (m, 2H, *o*-H in P(S)Ph), 9.36 (d, 1H, H(C6) or H(C3), ^3^*J*_HH_ = 8.3 Hz) ppm. ^13^C{^1^H} NMR (100.61 MHz, CDCl_3_): *δ* 65.32 (d, CH, ^1^*J*_CP_ = 70.5 Hz), 122.19 (s, C3 or C6), 124.17 (d, *ipso*-C in P(S)Ph, ^1^*J*_CP_ = 74.8 Hz), 124.64 (s, C6 or C3), 125.70 (d, *ipso*-C in P(S)Ph, ^1^*J*_CP_ = 78.3 Hz), 127.46 (s, C4 or C5), 128.17–128.22 (m, overlapping signals of *o*-C and *m*-C in Ph and C5 or C4), 128.72 (d, *m*-C in P(S)Ph, ^3^*J*_CP_ = 12.4 Hz), 128.97 (d, *p*-C in Ph, ^5^*J*_CP_ = 3.5 Hz), 129.97 (d, *m*-C in P(S)Ph, ^3^*J*_CP_ = 12.4 Hz), 132.46 (s, *ipso*-C in Ph or C2), 132.58 (d, *o*-C in P(S)Ph, ^2^*J*_CP_ = 9.5 Hz), 132.85 (d, *o*-C in P(S)Ph, ^2^*J*_CP_ = 10.0 Hz), 133.42 (s, C2 or *ipso*-C in Ph), 133.79 (d, *p*-C in P(S)Ph_2_, ^4^*J*_CP_ = 2.8 Hz), 150.26 (s, C7), 166.52 (d, C=O, ^3^*J*_CP_ = 15.1 Hz), 170.83 (s, C1) ppm. IR (KBr, *ν*/cm^−1^): 486(w), 523(m), 581(w) (*ν*P=S), 687(m), 708(m), 747(m), 757(m), 913(vw), 923(vw), 998(vw), 1106(m), 1157(w), 1187(w), 1251(vw), 1318(w), 1373(w), 1437(m), 1457(m), 1480(m), 1506(w), 1559(w), 1630(vs) (*ν*C=O), 2906(vw), 3060(w). Anal. Calcd for C_27_H_20_ClN_2_OPPdS_2_: C, 51.85; H, 3.22; N, 4.48. Found: C, 51.55; H, 3.29; N, 4.42%.

**[κ^3^-*S*,*N*,*S*-(L)Pd(II)Cl] complex 13a**


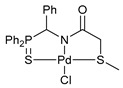


The compound was obtained as a mixture of diastereomers arising due to the configuration stabilization of the thioether sulfur atom as a result of coordination in addition to the existing chiral carbon center. Yield: 85 mg (97%). ^31^P{^1^H} NMR (161.98 MHz, CDCl_3_): *δ* 64.82 (minor isomers (m) 39%), 67.28 (major isomers (M) 61%) ppm. ^1^H NMR (400.13 MHz, CDCl_3_): *δ* 2.45 (s, 3H, Me (m)), 2.64 (s, 3H, Me (M)), 3.18 (d, 1H, CH_2_S (M), ^2^*J*_HH_ = 16.8 Hz), 3.32 (d, 1H, CH_2_S (m), ^2^*J*_HH_ = 16.2 Hz), 3.68 (d, 1H, CH_2_S (M), ^2^*J*_HH_ = 16.8 Hz), 3.70 (d, 1H, CH_2_S (m), ^2^*J*_HH_ = 16.2 Hz), 6.52 (d, 1H, CH (m), ^2^*J*_HP_ = 3.2 Hz), 6.56 (d, 1H, CH (M), ^2^*J*_HP_ = 3.1 Hz), 7.08–7.29 (m, 7H, H_Ar_ (M) + 7H, H_Ar_ (m)), 7.45–7.50 (m, 1H, H_Ar_ (M) + 1H, H_Ar_ (m)), 7.55–7.57 (m, 2H, H_Ar_ (m)), 7.66–7.80 (m, 5H, H_Ar_ (M) + 3H, H_Ar_ (m)), 8.07 (dd, 2H, *o*-H in P(S)Ph (M), ^3^*J*_HP_ = 12.6 Hz, ^3^*J*_HH_ = 7.7 Hz), 8.15 (dd, 2H, *o*-H in P(S)Ph (m), ^3^*J*_HP_ = 12.4 Hz, ^3^*J*_HH_ = 7.5 Hz) ppm. IR (KBr, *ν*/cm^−1^): 474(w), 520(m), 584(m) (*ν*P=S), 687(m), 708(m), 749(m), 804(w), 851(vw), 999(w), 1027(vw), 1108(m), 1182(w), 1315(w), 1340(w), 1360(m), 1420(w), 1437(m), 1452(w), 1490(w), 1594(vs), 1602(vs) (*ν*C=O), 2906(w), 3056(w). Anal. Calcd for C_22_H_21_ClNOPPdS_2_: C, 47.84; H, 3.83; N, 2.54. Found: C, 47.71; H, 3.92; N, 2.64%.

**[κ^3^-*S*,*N*,*S*-(L)Pd(II)Cl] complex 13b**


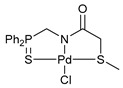


Yield: 61 mg (81%). ^31^P{^1^H} NMR (161.98 MHz, CDCl_3_): *δ* 59.26 ppm. ^1^H NMR (400.13 MHz, CDCl_3_): *δ* 2.56 (s, 3H, Me), 3.27 (d, 1H, CH_2_S, ^2^*J*_HH_ = 16.2 Hz), 3.70 (d, 1H, CH_2_S, ^2^*J*_HH_ = 16.2 Hz), 4.56 (dd, 1H, CH_2_P, ^2^*J*_HP_ = 4.2 Hz, ^2^*J*_HH_ = 14.8 Hz), 4.86 (dd, 1H, CH_2_P, ^2^*J*_HP_ = 3.5 Hz, ^2^*J*_HH_ = 14.8 Hz), 7.55–7.61 (m, 4H, *m*-H in P(S)Ph_2_), 7.66–7.70 (m, 2H, *p*-H in P(S)Ph_2_), 7.77–7.86 (m, 4H, *o*-H in P(S)Ph_2_) ppm. ^13^C{^1^H} NMR (100.61 MHz, CDCl_3_): *δ* 23.66 (s, Me), 41.66 (s, CH_2_S), 54.94 (d, CH_2_P, ^1^*J*_CP_ = 76.5 Hz), 125.11 (d, *ipso*-C in P(S)Ph, ^1^*J*_CP_ = 79.2 Hz), 125.40 (d, *ipso*-C in P(S)Ph, ^1^*J*_CP_ = 78.6 Hz), 129.58 (d, *m*-C in P(S)Ph, ^3^*J*_CP_ = 12.5 Hz), 129.61 (d, *m*-C in P(S)Ph, ^3^*J*_CP_ = 12.5 Hz), 132.03 (d, *o*-C in P(S)Ph, ^2^*J*_CP_ = 10.6 Hz), 132.05 (d, *o*-C in P(S)Ph, ^2^*J*_CP_ = 10.3 Hz), 133.72 (d, *p*-C in P(S)Ph, ^4^*J*_CP_ = 2.8 Hz), 133.75 (d, *p*-C in P(S)Ph, ^4^*J*_CP_ = 2.8 Hz) ppm (the signal of C=O carbon nucleus was not observed). IR (KBr, *ν*/cm^−1^): 448(w), 513(m), 584(m) (*ν*P=S), 687(m), 703(m), 718(m), 751(m), 799(w), 845(w), 998(w), 1110(m), 1176(w), 1312(w), 1364(m), 1437(m), 1484(w), 1601(s) (*ν*C=O), 2884(vw), 2921(w), 3055(vw). Anal. Calcd for C_16_H_17_ClNOPPdS_2_: C, 40.35; H, 3.60; N, 2.94. Found: C, 40.41; H, 3.65; N, 3.02%.

**[κ^3^-*S*,*N*,*S*-(L)Pd(II)Cl] complex 14**


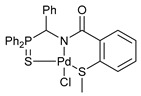


The compound was obtained as a mixture of diastereomers arising due to the configuration stabilization of the thioether sulfur atom as a result of coordination in addition to the existing chiral carbon center. Yield: 75 mg (77%). ^31^P{^1^H} NMR (202.45 MHz, CDCl_3_, 258 K): *δ* 59.28 (major isomers (M) 68%), 60.46 (minor isomers (m) 32%) ppm. For the ^1^H and ^13^C{^1^H} NMR spectra, see the SI. IR (KBr, *ν*/cm^−1^): 485(w), 514(w), 530(m), 579(w) and 589(w) (both *ν*P=S), 689(m), 708(m), 746(m), 786(w), 968(w), 998(w), 1027(w), 1105(m), 1145(w), 1187(w), 1338(br, m), 1414(w), 1437(m), 1492(w), 1547(s), 1580(s) (*ν*C=O), 2908(vw), 3025(w), 3056(w). Anal. Calcd for C_27_H_23_ClNOPPdS_2_: C, 52.78; H, 3.77; N, 2.28. Found: C, 52.77; H, 3.91; N, 2.41%.

#### 3.2.3. Synthesis of Optically Active Isopropyl-Substituted Derivatives **16**–**18** 

**(*R*)-(1-Amino-2-methylpropyl)diphenylphosphine sulfide hydrochloride (*R*)-16**


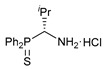


A total of 4.0 M HCl in dioxane (0.25 mL, 1.000 mmol) was slowly added dropwise to a stirred solution of compound **15** (0.200 g, 0.528 mmol) in MeOH (5 mL) at 0–5 °C under an argon atmosphere. The reaction mixture was stirred at room temperature for 3 h and left overnight. The solvent was removed under reduced pressure. The resulting residue was crystallized upon addition of Et_2_O. The mother liquor was decanted, and the obtained precipitate was dried under a vacuum to give 0.160 g of the target product as white crystals. Yield: 93%. Mp: 115–120 °C. ^31^P{^1^H} NMR (161.98 MHz, CD_3_OD): *δ* 44.91 ppm. ^1^H (400.13 MHz, CD_3_OD): *δ* 1.05 (d, 3H, Me, ^3^*J*_HH_ = 7.0 Hz), 1.13 (d, 3H, Me, ^3^*J*_HH_ = 7.0 Hz), 2.16–2.20 (m, 1H, CH in *^i^*Pr), 3.32–3.34 (m, 3H, NH_3_Cl), 4.68–4.71 (m, 1H, CH), 7.57–7.66 (m, 6H, *m*-H and *p*-H in P(S)Ph_2_), 8.09–8.15 (m, 2H, *o*-H in P(S)Ph), 8.18–8.23 (m, 2H, *o*-H in P(S)Ph) ppm.

**(*R*)-*N*-[1-(Diphenylthiophosphoryl)-2-methylpropyl]picolinamide (*R*)-17**


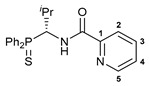


A solution of triethylamine (0.114 g, 1.127 mmol) in CH_2_Cl_2_ (3 mL) was added dropwise to a stirred solution of amine hydrochloride (***R***)-**16** (0.147 g, 0.451 mmol) in CH_2_Cl_2_ (5 mL) at –5 °C. The resulting mixture was stirred upon cooling for 15 min. Then, a solution of picolinoyl chloride generated in situ from picolinic acid (0.056 g, 0.455 mmol), SOCl_2_ (0.054 g, 0.454 mmol), and Et_3_N (0.070 g, 0.692 mmol) in CH_2_Cl_2_ (8 mL) was slowly added dropwise. The reaction mixture was stirred at room temperature for 12 h and then sequentially washed with water, an aqueous solution of NaHCO_3_, and again with water. The organic layer was separated, dried over anhydrous Na_2_SO_4_, and evaporated to dryness. The residue obtained was crystallized upon the addition of Et_2_O. The resulting precipitate was collected by filtration to provide 0.089 g of the target product as beige crystals. Yield: 50%. Mp: 210–212 °C. ^31^P{^1^H} NMR (161.98 MHz, CDCl_3_): *δ* 48.45 ppm. ^1^H NMR (500.13 MHz, CDCl_3_): *δ* 0.89 (d, 3H, Me, ^3^*J*_HH_ = 6.7 Hz), 1.11 (d, 3H, Me, ^3^*J*_HH_ = 6.7 Hz), 2.27–2.35 (m, 1H, CH in *^i^*Pr), 5.38–5.42 (m, 1H, CH), 7.28–7.36 (m, 3H, *m*-H and *p*-H in P(S)Ph), 7.40–7.42 (m, 1H, H(C4)), 7.51–7.57 (m, 3H, *m*-H and *p*-H in P(S)Ph), 7.79 (dt, 1H, H(C3), ^3^*J*_HH_ = 7.6 Hz, ^4^*J*_HH_ = 1.1 Hz), 7.92–7.96 (m, 2H, *o*-H in P(S)Ph), 8.03–8.09 (m, 3H, H(C2) + *o*-H in P(S)Ph), 8.58 (d, 1H, H(C5), ^3^*J*_HH_ = 4.4 Hz), 8.75 (br. d, 1H, NH, ^3^*J*_HH_ = 10.5 Hz) ppm. ^13^C{^1^H} NMR (125.76 MHz, CDCl_3_): *δ* 18.28 (d, Me, ^3^*J*_CP_ = 4.3 Hz), 22.27 (d, Me, ^3^*J*_CP_ = 9.4 Hz), 30.03 (d, CH in *^i^*Pr, ^2^*J*_CP_ = 6.1 Hz), 52.01 (d, CH, ^1^*J*_CP_ = 61.3 Hz), 122.21 (s, C2), 126.35 (s, C4), 128.31 (d, *m*-C in P(S)Ph, ^3^*J*_CP_ = 11.8 Hz), 128.76 (d, *m*-C in P(S)Ph, ^3^*J*_CP_ = 11.8 Hz), 131.21 (d, *ipso*-C in P(S)Ph, ^1^*J*_CP_ = 75.4 Hz), 131.35 (d, *o*-C in P(S)Ph, ^2^*J*_CP_ = 10.0 Hz), 131.40 (d, *p*-C in P(S)Ph, ^4^*J*_CP_ = 2.7 Hz), 131.76 (d, *o*-C in P(S)Ph, ^2^*J*_CP_ = 10.0 Hz), 131.84 (d, *ipso*-C in P(S)Ph, ^1^*J*_CP_ = 80.0 Hz), 131.86 (d, *p*-C in P(S)Ph, ^4^*J*_CP_ = 3.0 Hz), 137.15 (s, C3), 148.43 (s, C5), 148.96 (s, C1), 164.19 (d, C=O, ^3^*J*_CP_ = 4.8 Hz) ppm. IR (KBr, *ν*/cm^−1^): 482(w), 512(m), 522(m), 597(w), 614(w) and 627(m) (both *ν*P=S), 654(w), 690(m), 696(m), 713(m), 745(m), 754(m), 774(m), 817(w), 899(vw), 998(w), 1024(w), 1040(w), 1096(m), 1121(w), 1158(w), 1181(w), 1240(w), 1262(w), 1289(w), 1301(w), 1314(w), 1340(w), 1370(w), 1389(w), 1435(m), 1464(m), 1482(m), 1508(s) (C(O)NH), 1569(w), 1591(w), 1680(s) (*ν*C=O), 2850(w), 2920(m), 2960(w), 3049(w), 3365(m) (*ν*NH). Anal. Calcd for C_22_H_23_N_2_OPS: C, 66.99; H, 5.88; N, 7.10. Found: C, 67.34; H, 6.28; N, 6.81%.

**[κ^3^-*S*,*N*,*N*-(L)Pd(II)Cl] Pd(II) pincer complex (*R*)-18**


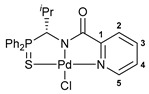


A solution of PdCl_2_(NCPh)_2_ (38 mg, 0.099 mmol) in CH_2_Cl_2_ (3 mL) was slowly added dropwise to a solution of ligand (***R***)-**17** (39 mg, 0.099 mmol) and Et_3_N (10 mg, 0.099 mmol) in CH_2_Cl_2_ (7 mL). The reaction mixture was left under ambient conditions for 3 h and then evaporated to dryness. The residue obtained was purified by column chromatography on silica gel (eluent: CH_2_Cl_2_–EtOH (100:1)) to provide the target complex as a yellow crystalline solid. Yield: 33 mg (62%). ^31^P{^1^H} NMR (121.49 MHz, CDCl_3_): *δ* 63.93 ppm. ^1^H NMR (500.13 MHz, CDCl_3_): *δ* 0.66 (d, 3H, Me, ^3^*J*_HH_ = 6.8 Hz), 1.15 (d, 3H, Me, ^3^*J*_HH_ = 6.8 Hz), 2.29–2.38 (m, 1H, CH in *^i^*Pr), 5.45 (d, 1H, CH, ^2^*J*_HP_ = 7.2 Hz), 7.48–7.51 (m, 1H, H(C4)), 7.52–7.66 (m, 6H, *m*-H and *p*-H in P(S)Ph_2_), 7.75 (dd, 2H, *o*-H in P(S)Ph, ^3^*J*_HP_ = 13.0 Hz, ^3^*J*_HH_ = 7.4 Hz), 7.88 (d, 1H, H(C2), ^3^*J*_HH_ = 7.4 Hz), 7.95–7.99 (m, 3H, H(C3) + *o*-H in P(S)Ph), 8.99 (d, 1H, H(C5), ^3^*J*_HH_ = 4.9 Hz) ppm. ^13^C{^1^H} NMR (125.76 MHz, CDCl_3_): *δ* 19.37 (d, Me, ^3^*J*_CP_ = 7.7 Hz), 21.90 (d, Me, ^3^*J*_CP_ = 4.9 Hz), 30.55 (d, CH in *^i^*Pr, ^2^*J*_CP_ = 4.1 Hz), 64.02 (d, CH, ^1^*J*_CP_ = 70.4 Hz), 125.58 (s, C2), 126.06 (d, *ipso*-C in P(S)Ph, ^1^*J*_CP_ = 69.3 Hz), 127.14 (s, C4), 128.32 (d, *ipso*-C in P(S)Ph, ^1^*J*_CP_ = 82.0 Hz), 129.36 (d, *m*-C in P(S)Ph, ^3^*J*_CP_ = 11.8 Hz), 129.66 (d, *m*-C in P(S)Ph, ^3^*J*_CP_ = 12.7 Hz), 131.73 (d, *o*-C in P(S)Ph, ^2^*J*_CP_ = 10.0 Hz), 132.69 (d, *o*-C in P(S)Ph, ^2^*J*_CP_ = 10.4 Hz), 133.07 (d, *p*-C in P(S)Ph, ^4^*J*_CP_ = 2.9 Hz), 133.68 (d, *p*-C in P(S)Ph, ^4^*J*_CP_ = 2.9 Hz), 139.45 (s, C3), 147.54 (s, C5), 153.82 (s, C1), 171.54 (d, C=O, ^3^*J*_CP_ = 12.4 Hz) ppm. IR (KBr, *ν*/cm^−1^): 497(m), 518(m), 579(m) (*ν*P=S), 684(m), 708(m), 718(w), 748(m), 808(w), 998(w), 1049(w), 1105(m), 1184(vw), 1289(w), 1358(m), 1437(m), 1459(w), 1598(s), 1627(s) (*ν*C=O), 2887(vw), 2929(w), 2963(w), 3055(vw). Anal. Calcd for C_22_H_22_N_2_OPPdS: C, 49.36; H, 4.14; N, 5.23. Found: C, 49.30; H, 4.23; N, 5.23%.

### 3.3. X-Ray Crystallography

Single crystals of the compounds explored were obtained by slow crystallization from a CH_2_Cl_2_–hexane (**8**, **11**), CHCl_3_–Et_2_O (**9**, 1**0a**, **13b**), CH_2_Cl_2_–Et_2_O (**10b**, (***R***)-**18**), CHCl_3_–CH_2_Cl_2_–Et_2_O (**12**, **18**), CHCl_3_–EtOH–hexane (**13a**), MeOH (**16**), and EtOAc ((***R***)-**17**). X-ray diffraction data for compounds **8** and **9** were collected at 120 K with a Bruker APEXII CCD diffractometer, those for **10a**, **10b**, **11**, **13a**, and **13b** were collected at 120 K with a Bruker APEXII DUO diffractometer, while those for **12**, **16**, **18**, (***R***)-**17**, and (***R***)-**18** were collected at 100 K with a Bruker Quest D8 CMOS diffractometer, all using graphite monochromated Mo-Kα radiation (λ = 0.71073 Å). Using Olex2 [37], the structures were solved with the ShelXT [38] structure solution program using Intrinsic Phasing and refined with the XL [39] refinement package using Least-Squares minimization against F^2^_hkl_ in anisotropic approximation for non-hydrogen atoms. Hydrogen atoms of NH groups in **8**, (***R***)-**17,** and **16** and hydrogen atoms of lattice ethanol molecule in **13a** were located using difference Fourier synthesis, the positions of other hydrogen atoms were calculated, and they all were refined in isotropic approximation within the riding model. Crystal data and structure refinement parameters are given in Table S2 in the SI. CCDC 2435219 (**8**), 2435227 (**9**), 2435218 (**10a**), 2435221 (**10b**), 2435228 (**11**), 2435226 (**12**), 2435225 (**13a**), 2435217 (**13b**), 2435224 (**16**), 2435222 (**18**), 2435220 ((***R***)-**17**), and 2435223 ((***R***)-**18**) contain the supplementary crystallographic data.

### 3.4. Cytotoxicity Studies

The cytotoxicity of the compounds obtained was investigated on human colorectal carcinoma (HCT116), breast cancer (MCF7), prostate adenocarcinoma (PC3), glioblastoma (U251), ovarian adenocarcinoma (Scov3), chronic myelogenous leukemia (K562 and K562/iS9), multiple plasmacytoma (AMO1), and acute lymphoblastic leukemia (H9) cell lines, as well as human embryonic kidney (HEK293) and mammary epithelial (HBL100 and HBL100/Dox) cells used as non-cancerous cell lineages. All the cell lines were obtained from American Type Culture Collection (ATCC). The tested compounds were initially dissolved in DMSO. Cisplatin was obtained from a commercial source (as an infusion concentrate in natural saline solution). The experiments were performed using the conventional MTT assay (ICN Biomedicals, Eschwege, Germany) according to the previously published procedure [29].

To evaluate the cytotoxicity of Dox to the selected cell lines in the presence of enantiomerically pure and racemic complexes **18**, HBL-100, HBL-100/Dox, K562, and K562/iS9 cells were incubated for 72 h with subtoxic concentrations of (***R***)-**18** or **18** (2 μM for K562 and K562/iS9 cells, 5 μM for HBL100 and HBL100/Dox cells) in the presence of different concentrations of Dox. The results are presented in Figure S59 in the SI.

### 3.5. Apoptosis Induction Assay

The apoptosis-inducing ability of enantiomerically pure and racemic complexes **18** was studied on K562 and K562/iS9 cells that were cultured in a medium containing 10 μM of the tested palladocycle for 20 h. The experiments were performed following the published procedure [29]. The analysis was carried out on a FACScan flow cytometer (Becton Dickinson, Franklin Lakes, NJ, USA) using the CellQuest software (version 3.3).

### 3.6. Evaluation of P-gp Functional Activity

The functional activity of P-gp was evaluated by the method described earlier [40]. K562/iS9 cells (3 × 10^6^) were incubated for 15 min in a culture medium containing 5 μg/mL of Rh123 (Sigma-Aldrich, St. Louis, MO, USA). After incubation, the cells were washed twice and divided into several fractions. One fraction was incubated in pure medium, and the others were incubated with the addition of 20 μM of complex (***R***)-**18** or **18**. The incubation was carried out in the full culture medium at 37 °C for 30 min. Elacridar, a potent third-generation P-gp inhibitor, was used as a positive control. The cell fluorescence was evaluated using a flow cytometer (Becton Dickinson). The results were analyzed using the CellQuest software.

### 3.7. Antibacterial Activity Tests

The microbial strains *Bacillus subtillis* (VKM B-501), *Micrococcus luteus* (VKM Ac-2230), and *Groenewaldozyma auringiensis* (VKM Y-2927) were used for antimicrobial activity tests. All strains were obtained from the All-Russian Collection of Microorganisms (VKM) at the Skryabin Institute of Biochemistry and Physiology of Microorganisms, Pushchino Scientific Center of Biological Research. *B. subtilis* and *M. luteus* were grown on the following medium (g/L): aminopeptide 60 mL; tryptone 5.0 g; yeast extract 1.0 g; soybean extract 30 mL; bacto-agar 15.0 g; final pH 7.2. *G. auringiensis* was grown on malt extract medium containing the following medium (g/L): malt extract 12.75; dextrin 2.75; glycerol 2.35; gelatin peptone 0.78; bacto-agar 15; final pH 5.4. The antimicrobial activity of ligand **17** and its cyclopalladated derivative **18** (both in the racemic form) was studied by the agar well diffusion method. The overnight grown culture of each strain (100 µL) was applied to the surface of the solid medium, then spread on agar plates using a glass spreader, and left for 30 min. The wells were cut in the culture plates using a sterilized glass tube (4 mm in diameter), and each compound sample was dissolved in DMSO to a final concentration of 0.25 or 2.5 mM (10 µL) and was placed in each well. After this, the plates were kept at room temperature for 3 h to improve the diffusion of the compounds and incubated at 28 °C for 24 h. DMSO (15%) was used as a negative control, while kanamycin (50 mg/mL) was used as a positive control. The antimicrobial activity was determined by measuring the inhibition zone around each well as a mean of three replicates.

## 4. Conclusions

To summarize the results presented, an extended family of functionalized amide ligands based on different carboxylic acids and thiophosphoryl-substituted alkylamines were synthesized and shown to readily form Pd(II) pincer complexes with *S*,*N*,*N*- or *S*,*N*,*S*-donor sets. The cytotoxicity studies revealed the high activity of the hemilabile derivatives based on picolinamides, especially towards blood cancer cell lines. Using sulfur chirality, a convenient synthetic route to the optically active ligand bearing an isopropyl substituent in the bridging unit of the amine component was devised, the cyclopalladated derivative of which also provided prominent cytotoxic effects, slightly outperforming the racemic sample. Additional investigations with these promising candidates ascertained their remarkable cytotoxicity against doxorubicin-resistant cell lines, high apoptosis induction ability, and low, if any, affinity to P-gp, suggesting the high potential to overcome drug resistance associated with P-gp overexpression. The racemic palladocycle with an isopropyl substituent in the ligand framework also exhibited moderate antibacterial activity, which expands the bioactivity spectrum of thiophosphoryl-appended Pd(II) pincer complexes with a deprotonated amide core.

## Figures and Tables

**Table 1:** The cytotoxicities of the complexes obtained against selected human solid cancer and non-cancerous cell lines.

Entry	Compound	IC_50_ ± SD, ^a^ μM					
		HCT116	MCF7	PC3	HEK293	HBL100	HBL100/Dox
1	**Ia** (Ref. [28])	3.0 ± 0.5	10.0 ± 2.5	4.0 ± 0.4	6.2 ± 0.5	16.0 ± 0.5	34.0 ± 4.0
2	**9**	7.0 ± 0.5	6.2 ± 2.0	8.0 ± 0.5	10.0 ± 4.0	6.5 ± 1.5	10.5 ± 1.5
3	**10a**	22.0 ± 2.0	>65.0	33.0 ± 3.0	15.0 ± 4.0	>60	>60
4	**10b**	14.0 ± 4.0	31.0 ± 2.0	23.0 ± 1.0	20.0 ± 4.0	34.2 ± 2.8	46.0 ± 1.4
5	**11**	18.0 ± 2.0	46.0 ± 4.0	31.0 ± 1.0	41.0 ± 4.0	29.0 ± 5.0	41.0 ± 1.0
6	**12**	11.3 ± 3.1	19.0 ± 4.2	10.0 ± 2.8	6.5 ± 2.2	12.0 ± 3.5	6.7 ± 1.5
7	**13a**	17.0 ± 2.0	31.0 ± 6.0	30.0 ± 2.5	22.0 ± 6.0	32.0 ± 6.0	54.0 ± 5.6
8	**13b**	23.0 ± 7.0	40.0 ± 5.0	30.0 ± 2.0	34.0 ± 2.0	52 ± 4.0	>60
9	**14**	4.5 ± 1.1	9.2 ± 0.9	4.2 ± 1.4	4.9 ± 1.5	8.4 ± 0.5	2.1 ± 0.3
10	(***R***)-**18**	7.9 ± 0.9	12.4 ± 2.2	10.0 ± 1.5	10.4 ± 1.8	11.5 ± 2.5	15.8 ± 2.2
11	**18**	9.2 ± 1.8	19.2 ± 1.3	11.5 ± 1.5	9.5 ± 1.8	14.0 ± 1.4	23.3 ± 2.7
12	cisplatin	18.0 ± 2.0	25.0 ± 4.0	16.0 ± 3.0	12.5 ± 1.5	14.6 ± 3.6	23.6 ± 3.6

^a^ SD is the standard deviation of the value.

**Table 2:** The cytotoxicities of the complexes obtained against selected human hematopoietic cancer cell lines.

Entry	Compound	IC_50_ ± SD, ^a^ μM			
		K562	K562/iS9	AMO1	H9
1	**Ia** (Ref. [28])	7.4 ± 0.4	5.8 ± 1.2	2.4 ± 0.3	3.2 ± 0.4
2	**9**	8.0 ± 1.5	6.2 ± 1.8	2.4 ± 0.6	3.2 ± 0.4
3	**12**	4.2 ± 0.8	2.0 ± 0.4	3.7 ± 0.7	2.2 ± 0.7
4	**13a**	14.2 ± 2.2	15.2 ± 1.8	4.2 ± 0.3	4.0 ± 0.6
5	**14**	2.1 ± 0.2	1.7 ± 0.2	1.2 ± 0.3	2.3 ± 0.5
6	(***R***)-**18**	4.3 ± 1.3	5.2 ± 0.6	3.9 ± 0.2	3.3 ± 0.4
7	**18**	4.8 ± 1.2	6.4 ± 1.7	3.0 ± 1.2	3.4 ± 0.1
8	cisplatin	15.5 ± 0.5	16.0 ± 2.0	3.2 ± 0.6	3.0 ± 1.0

^a^ SD is the standard deviation of the value.

**Table 3:** Antimicrobial activity of complex 18.

Compound, Concentration		Inhibition Zone, mm		
		*B. subtillis* (VKM B-501)	*M. luteus* (VKM Ac-2230)	*G. auringiensis* (VKM Y-2927)
Complex **18**	0.25 mM	0.5 ± 0.2	0.5 ± 0.3	0
	2.50 mM	0.5 ± 0.2	4.0 ± 0.5	0
DMSO, 15%		0	0	0
Km, 50 mg/mL		10.0 ± 0.8	15.0 ± 1.0	0

## Data Availability

The data presented in this study are available in the article and Supplementary Materials.

## Supplementary Materials

The following supporting information can be downloaded at https://www.mdpi.com/article/10.3390/ijms26104536/s1.
