# Supplementary material for: Extended Family of Thiophosphoryl-Appended Pd(II) Pincer Complexes with a Deprotonated Amide Core: Synthesis and Biological Evaluation
Source: Int J Mol Sci. 2025 May 9;26(10):4536. doi: 10.3390/ijms26104536 (PMC12111564; doi:10.3390/ijms26104536)
Supplement: Supplementary file 1 [file ijms-26-04536-s001.zip › ijms-3593035-supplementary.pdf]

Supporting information

Extended family of thiophosphoryl-appended Pd(II) pincer complexes with a deprotonated amide core: synthesis and biological evaluation

Diana V. Aleksanyan,[a]* Svetlana G. Churusova,[a] Aleksandr V. Konovalov,[a] Ekaterina Yu. Rybalkina,[b] Lidia A. Laletina,[b] Yana V. Ryzhmanova,[c] Yulia V. Nelyubina,[a,d] Svetlana A. Soloveva,[a] Sergey E. Lyubimov,[a] Alexander S. Peregudov,[a] Zinaida S. Klemenkova,[a] and Vladimir A. Kozlov[a]

[a] *A. N. Nesmeyanov Institute of Organoelement Compounds, Russian Academy of Sciences, ul. Vavilova 28, str. 1, Moscow, 119334 Russia*

[b] *N. N. Blokhin National Medical Research Center of Oncology of the Ministry of Health of the Russian Federation, Kashirskoe shosse 23, Moscow, 115478 Russia*

[c] *Skryabin Institute of Biochemistry and Physiology of Microorganisms, Pushchino Scientific Center of Biological Research, Russian Academy of Sciences, pr. Nauki 5, Pushchino, 142292 Russia*

[d] *Federal Research Center of Problems of Chemical Physics and Medicinal Chemistry, Russian Academy of Sciences, pr. Akademika Semenova 1, Chernogolovka, Moscow Region, 142432 Russia*

*corresponding author: aleksanyan.diana@ineos.ac.ru

Table of contents

	Page
**Figure S1.** $^{31}P\{^1H\}$ NMR spectrum of ligand **3a** (161.98 MHz, $CDCl_3$)	S4
**Figure S2.** ^1H NMR spectrum of ligand **3a** (400.13 MHz, $CDCl_3$)	S5
**Figure S3.** $^{13}C\{^1H\}$ spectrum of ligand **3a** (100.61 MHz, $CDCl_3$)	S6
**Figure S4.** $^{31}P\{^1H\}$ NMR spectrum of complex **10a** (161.98 MHz, $CDCl_3$)	S7
**Figure S5.** ^1H NMR spectrum of complex **10a** (400.13 MHz, $CDCl_3$)	S8
**Figure S6.** $^{13}C\{^1H\}$ spectrum of complex **10a** (100.61 MHz, $CDCl_3$–$(CD_3)_2SO$)	S9
**Figure S7.** $^{31}P\{^1H\}$ NMR spectrum of ligand **7b** (161.98 MHz, $CDCl_3$)	S10
**Figure S8.** ^1H NMR spectrum of ligand **7b** (400.13 MHz, $CDCl_3$)	S11
**Figure S9.** $^{31}P\{^1H\}$ NMR spectrum of complex **13b** (161.98 MHz, $CDCl_3$)	S12
**Figure S10.** ^1H NMR spectrum of complex **13b** (400.13 MHz, $CDCl_3$)	S13
**Figure S11.** $^{13}C\{^1H\}$ spectrum of complex **13b** (100.61 MHz, $CDCl_3$)	S14
**Figure S12.** $^{31}P\{^1H\}$ NMR spectrum of ligand **8** (202.45 MHz, $CDCl_3$)	S15
**Figure S13.** ^1H NMR spectrum of ligand **8** (500.13 MHz, $CDCl_3$)	S16
**Figure S14.** $^{13}C\{^1H\}$ spectrum of ligand **8** (125.76 MHz, $CDCl_3$))	S17
**Figure S15.** ^1H–^1H COSY spectrum of ligand **8** (500.13 MHz, $CDCl_3$)	S18
**Figure S16.** Extended fragments of the ^1H–^1H COSY spectrum of ligand **8** (500.13 MHz, $CDCl_3$)	S19
**Figure S17.** HMQC spectrum of ligand **8** ($CDCl_3$)	S20
**Figure S18.** Extended fragment of the HMQC spectrum of ligand **8** ($CDCl_3$)	S21
**Figure S19.** ^1H–^{13}C HMBC spectrum of ligand **8** ($CDCl_3$)	S22

Figure S20. Extended fragments of the $^{1}H-^{13}C$ HMBC spectrum of ligand **8** ($CDCl_3$) S23

Figure S21. $^{31}P\{^{1}H\}$ NMR spectrum of complex **14** (202.45 MHz, $CDCl_3$, 258 K) S24

Figure S22. ^{1}H NMR spectrum of complex **14** (500.13 MHz, $CDCl_3$, 258 K) S25

Figure S23. Extended fragment of the ^{1}H NMR spectrum of complex **14** (500.13 MHz, $CDCl_3$, 258 K) S26

Figure S24. $^{13}C\{^{1}H\}$ spectrum of complex **14** (125.76 MHz, $CDCl_3$, 258 K) S27

Figure S25. Extended fragments of the $^{13}C\{^{1}H\}$ spectrum of complex **14** (125.76 MHz, $CDCl_3$, 258 K) S28

Figure S26. $^{1}H-^{1}H$ COSY spectrum of complex **14** (500.13 MHz, $CDCl_3$, 258 K) S29

Figure S27. Extended fragments of the $^{1}H-^{1}H$ COSY spectrum of complex **14** (500.13 MHz, $CDCl_3$, 258 K) S30

Figure S28. HSQC spectrum of complex **14** ($CDCl_3$, 258 K) S31

Figure S29. Extended fragment of the HSQC spectrum of complex **14** ($CDCl_3$, 258 K) S32

Figure S30. $^{1}H-^{13}C$ HMBC spectrum of complex **14** ($CDCl_3$, 258 K) S33

Figure S31. Extended fragments of the $^{1}H-^{13}C$ HMBC spectrum of complex **14** ($CDCl_3$, 258 K) S34

Figure S32. ^{31}P NMR spectra of the solutions of complexes **9** (left) and **10b** (right) in 1 h (top) or 3 days (bottom) after dissolution in $(CD_3)_2SO$ (161.98 MHz) S35

Figure S33. General view of ligand **8** S36

Table S1. Main bond lengths (Å) and angles (°) for the complexes explored S37

Figure S34. Enantiomeric excess of the isopropyl-substituted amine derived from enantiomerically pure amine chloride ***(R)*-16** upon treatment with Et_3N in benzene determined by HPLC (an Agilent 1100 chromatograph, Chiralcel OD 250 mm × 4.6 mm column, flow rate 1.0 mL/min, UV 254 nm, eluent: hexane/isopropanol/triethylamine = 98/2/0.2) S38

Figure S35. Enantiomeric excesses of amide ***(R)*-17** obtained from enantiomerically pure amine hydrochloride ***(R)*-16** (left) and amide **17** obtained from racemic amine hydrochloride **16** determined by HPLC (an Agilent 1100 chromatograph, Chiralcel OD 250 mm × 4.6 mm column, flow rate 0.7 mL/min, UV 254 nm, eluent: hexane/isopropanol/triethylamine = 99/1/0.5) S38

Figure S36. $^{31}P\{^{1}H\}$ NMR spectrum of ligand **17** (161.98 MHz, $CDCl_3$) S39

Figure S37. ^{1}H NMR spectrum of ligand **17** (500.13 MHz, $CDCl_3$) S40

Figure S38. Extended fragments of the ^{1}H NMR spectrum of ligand **17** (500.13 MHz, $CDCl_3$) S41

Figure S39. $^{13}C\{^{1}H\}$ spectrum of ligand **17** (125.76 MHz, $CDCl_3$) S42

Figure S40. Extended fragments of the $^{13}C\{^{1}H\}$ spectrum of ligand **17** (125.76 MHz, $CDCl_3$) S43

Figure S41. $^{1}H-^{1}H$ COSY spectrum of ligand **17** (500.13 MHz, $CDCl_3$) S44

Figure S42. Extended fragments of the $^{1}H-^{1}H$ COSY spectrum of ligand **17** (500.13 MHz, $CDCl_3$) S45

Figure S43. HMQC spectrum of ligand **17** ($CDCl_3$) S46

Figure S44. Extended fragments of the HMQC spectrum of ligand **17** ($CDCl_3$) S47

Figure S45. $^{1}H-^{13}C$ HMBC spectrum of ligand **17** ($CDCl_3$) S48

Figure S46. Extended fragments of the $^{1}H-^{13}C$ HMBC spectrum of ligand **17** ($CDCl_3$) S49

Figure S47. $^{31}P\{^{1}H\}$ NMR spectrum of complex **18** (121.49 MHz, $CDCl_3$) S50

Figure S48. ^{1}H NMR spectrum of complex **18** (500.13 MHz, $CDCl_3$) S51

Figure S49. Extended fragments of the ^{1}H NMR spectrum of complex **18** (500.13 MHz, $CDCl_3$) S52

Figure S50. $^{13}C\{^{1}H\}$ spectrum of complex **18** (125.76 MHz, $CDCl_3$) S53

Figure S51. Extended fragments of the $^{13}C\{^{1}H\}$ spectrum of complex **18** (125.76 MHz, $CDCl_3$) S54

Figure S52. $^{1}H{-}^{1}H$ COSY spectrum of complex **18** (500.13 MHz, $CDCl_3$) S55
Figure S53. Extended fragments of the $^{1}H{-}^{1}H$ COSY spectrum of complex **18** (500.13 MHz, $CDCl_3$) S56
Figure S54. HMQC spectrum of complex **18** ($CDCl_3$) S57
Figure S55. Extended fragments of the HMQC spectrum of complex **18** ($CDCl_3$) S58
Figure S56. $^{1}H{-}^{13}C$ HMBC spectrum of complex **18** ($CDCl_3$) S59
Figure S57. Extended fragments of the $^{1}H{-}^{13}C$ HMBC spectrum of complex **18** ($CDCl_3$) S60
Figure S58. General views of racemic amine hydrochloride **16** (left) and palladocycle **18** (right) S61
Table S2. Crystal data and structure refinement parameters for compounds **8**, **9**, **10a**, **10b**, **11**, **12**, **13a**, **13b**, **16**, **18**, **(*R*)-17**, and **(*R*)-18** S62
Figure S59. Cytotoxic activity of doxorubicin against parental and doxorubicin-resistant cells upon combined incubation with subtoxic concentrations of complexes **(*R*)-18** and **18** (2 μM for K562 and K562/iS9 cells, 5 μM for HBL100 and HBL100/Dox cells) according to the results of the MTT assay (Dox – solid line, Dox + **18** (dashed line), Dox + **(*R*)-18** (dash-dotted line) S64

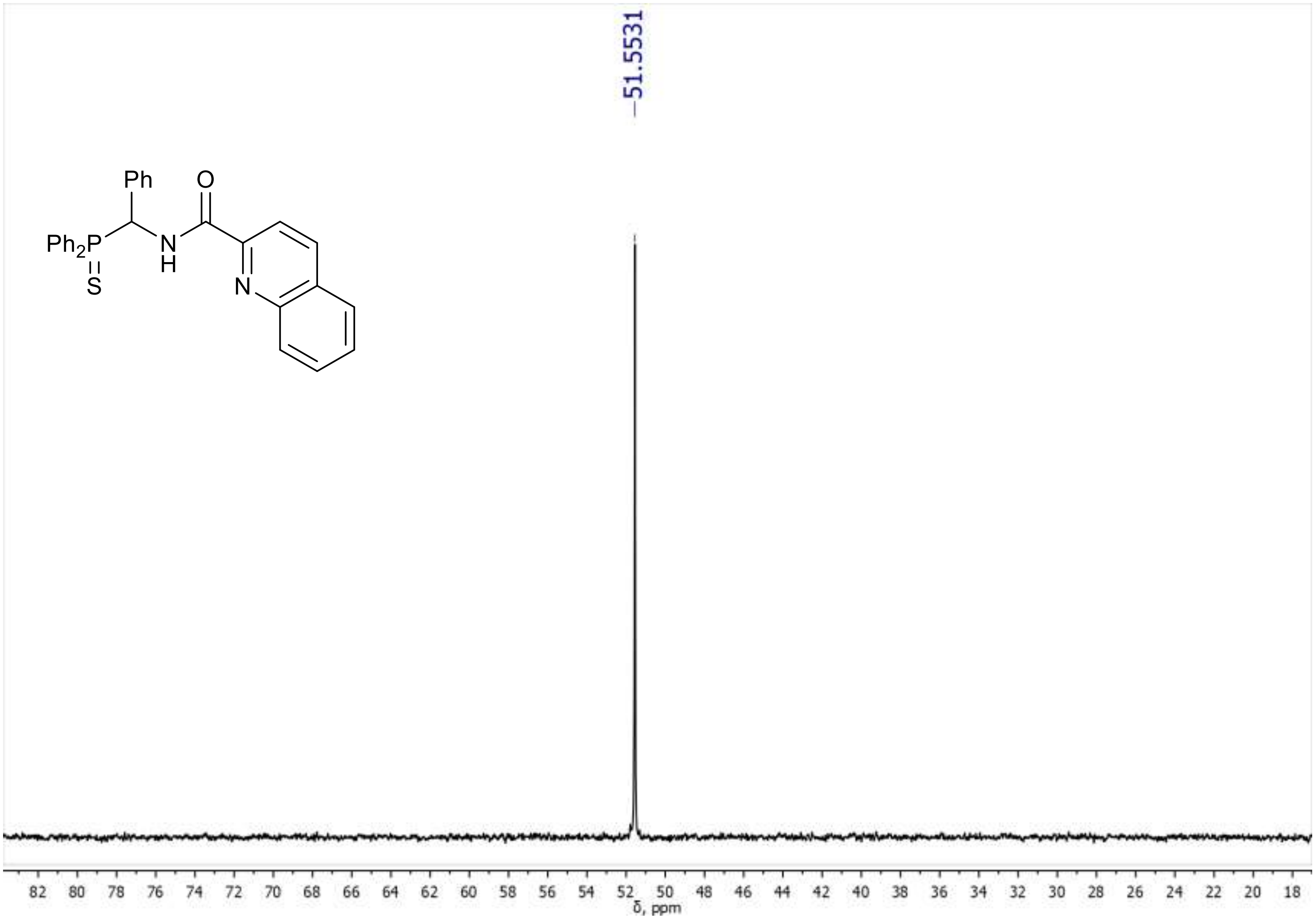

Figure S1. $^{31}P\{^{1}H\}$ NMR spectrum of ligand **3a** (161.98 MHz, $CDCl_3$)

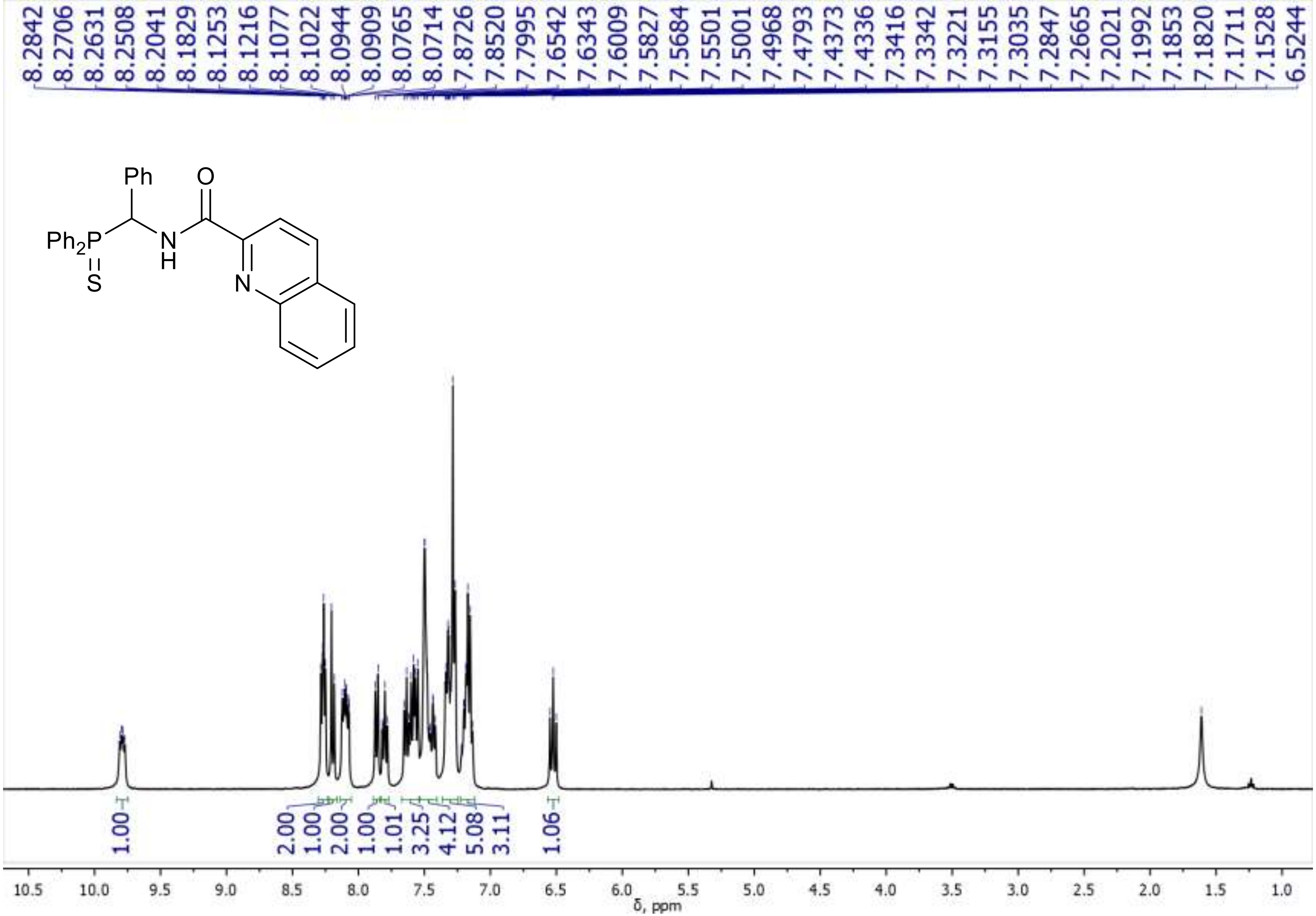

Figure S2. ^{1}H NMR spectrum of ligand **3a** (400.13 MHz, $CDCl_3$)

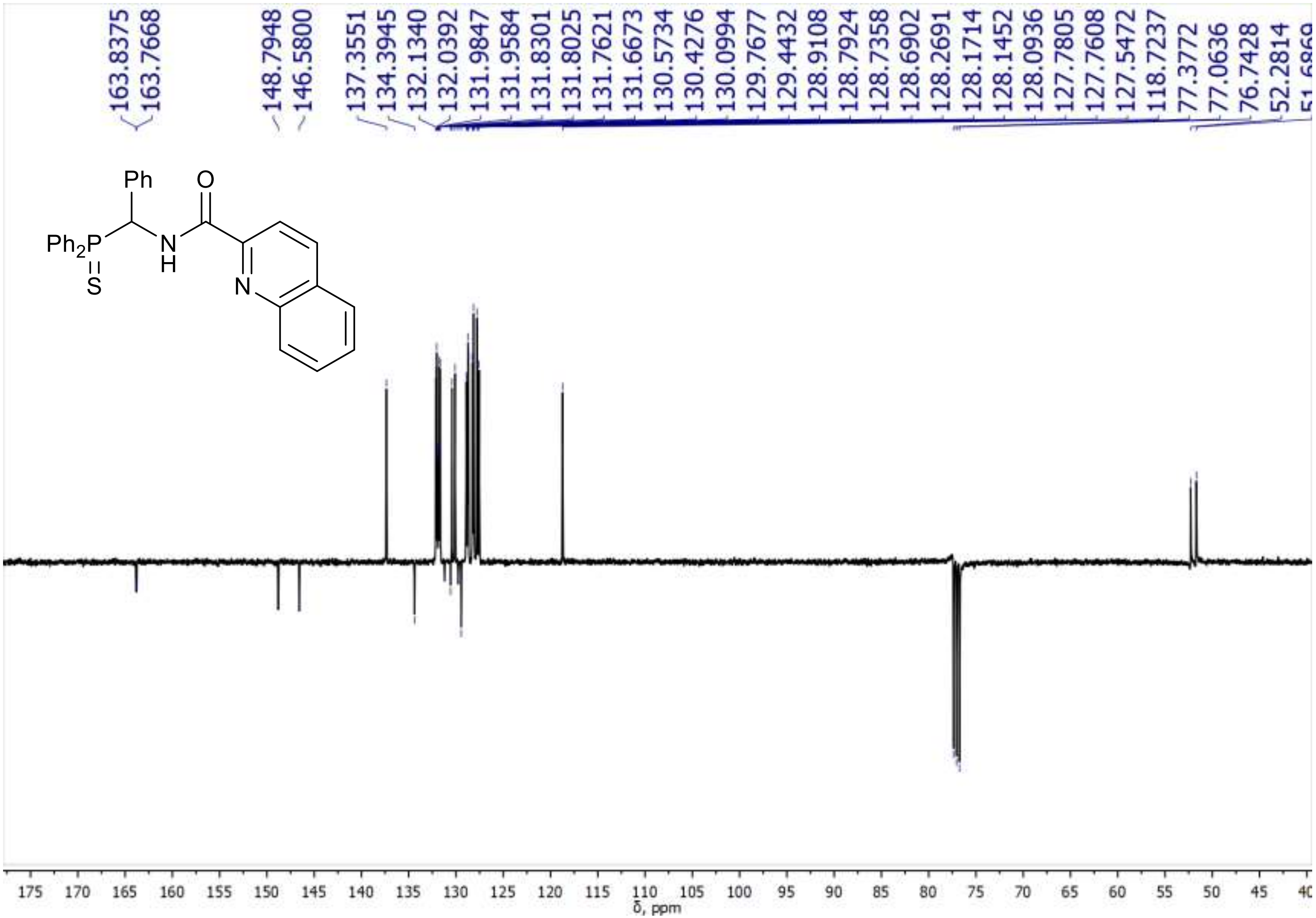

Figure S3. $^{13}C\{^{1}H\}$ spectrum of ligand **3a** (100.61 MHz, $CDCl_3$)

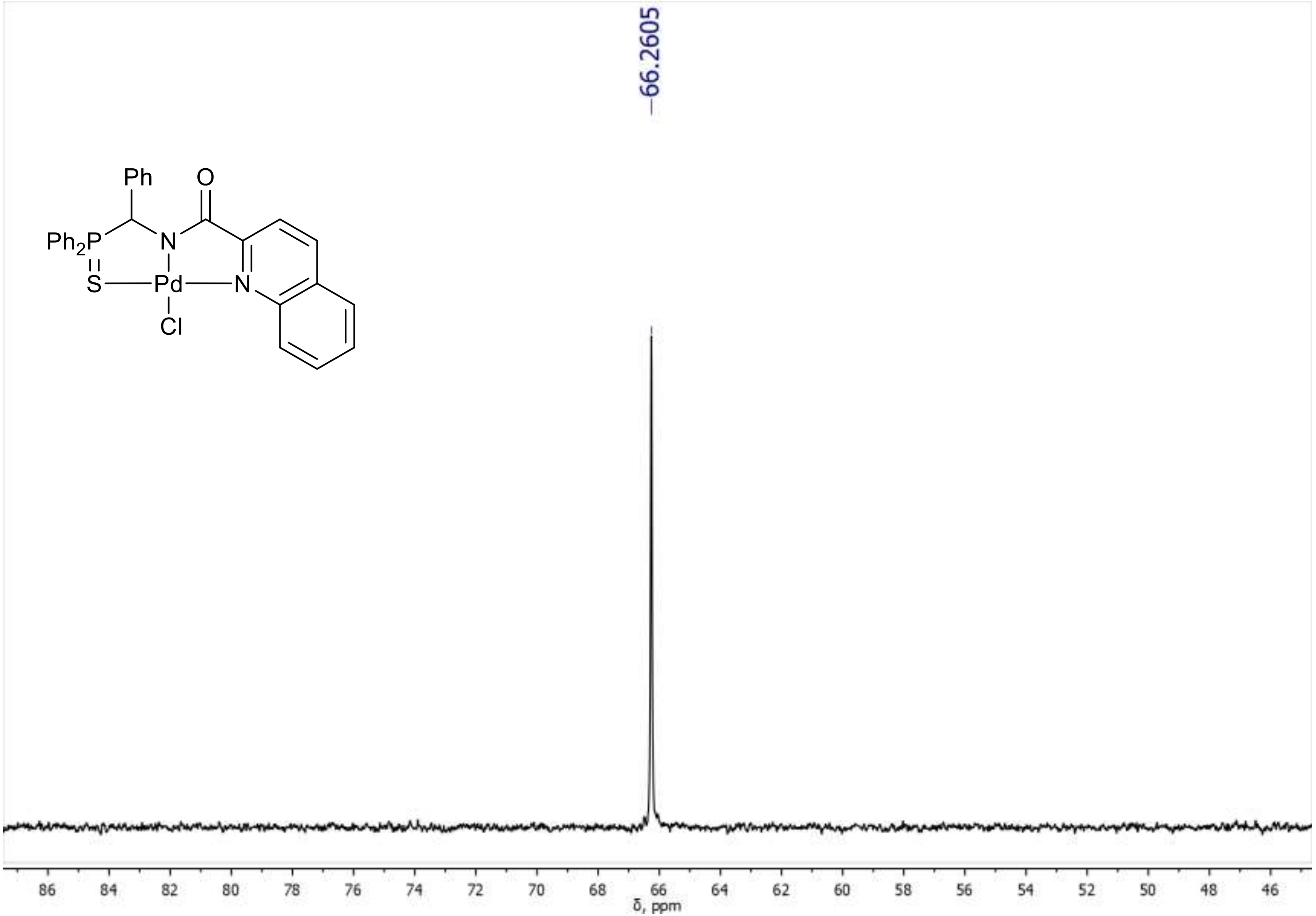

Figure S4. $^{31}P\{^{1}H\}$ NMR spectrum of complex **10a** (161.98 MHz, $CDCl_3$)

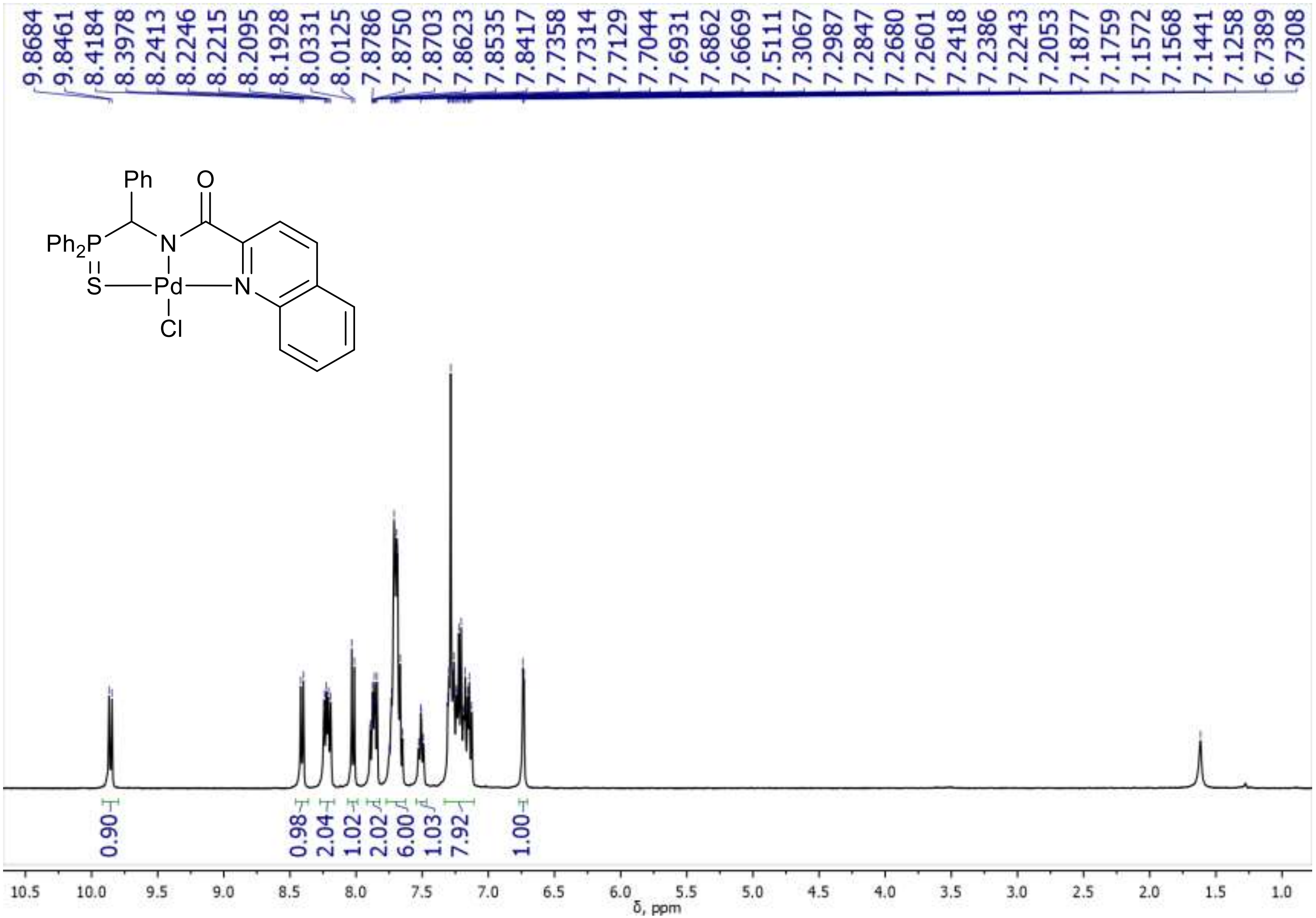

Figure S5. ^{1}H NMR spectrum of complex **10a** (400.13 MHz, $CDCl_3$)

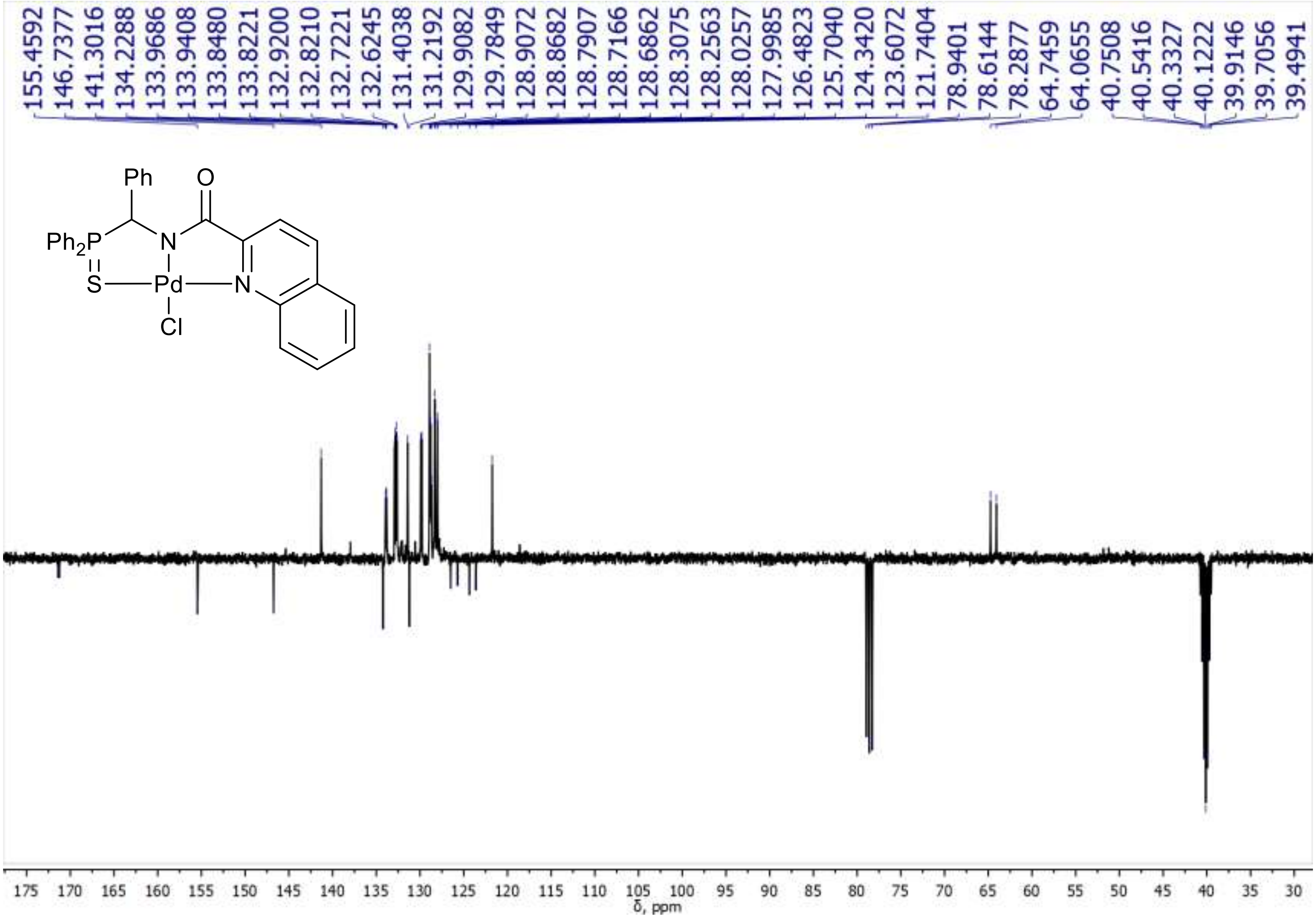

Figure S6. $^{13}C\{^{1}H\}$ spectrum of complex **10a** (100.61 MHz, $CDCl_3$–$(CD_3)_2SO$)

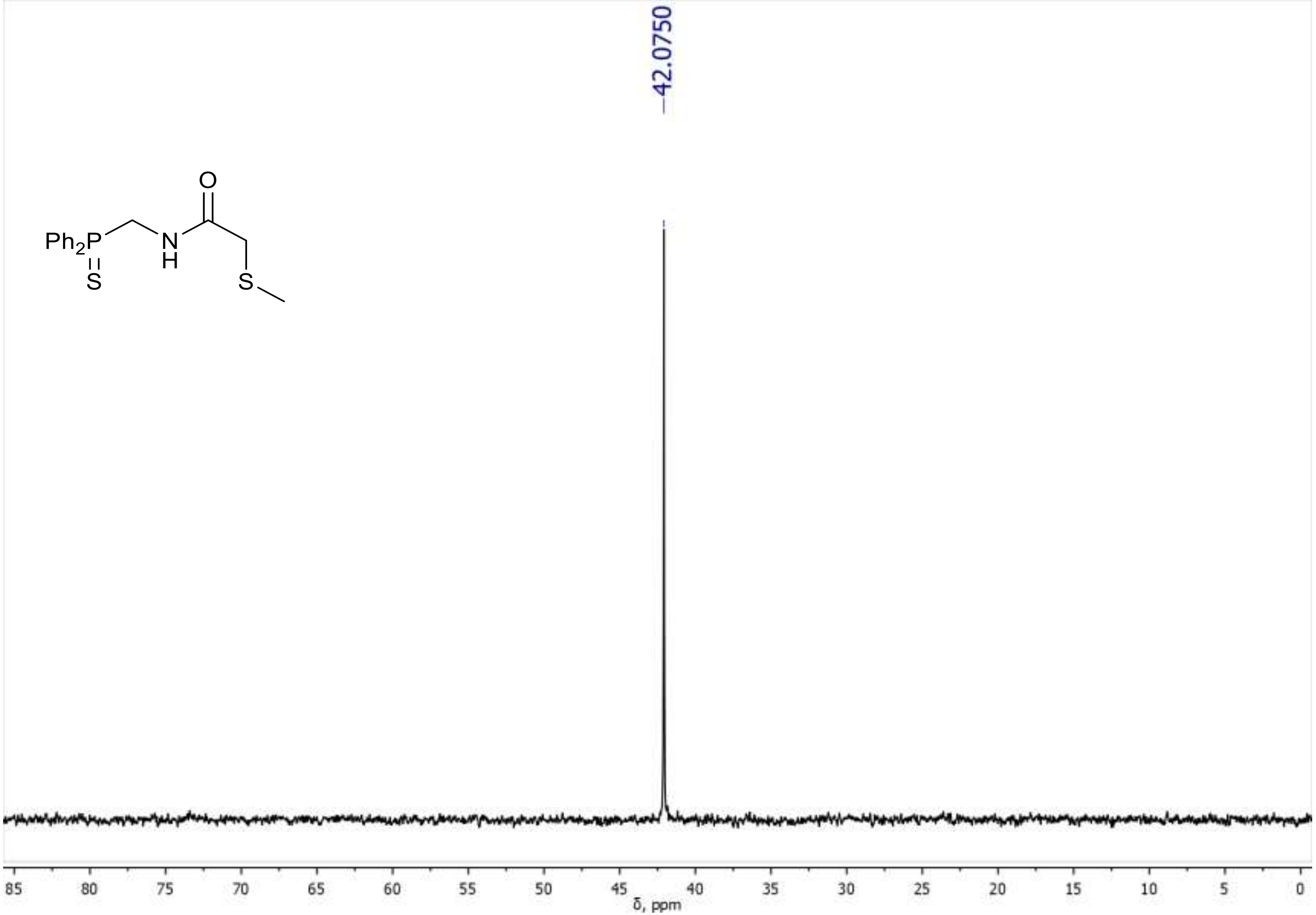

Figure S7. $^{31}P\{^{1}H\}$ NMR spectrum of ligand **7b** (161.98 MHz, $CDCl_3$)

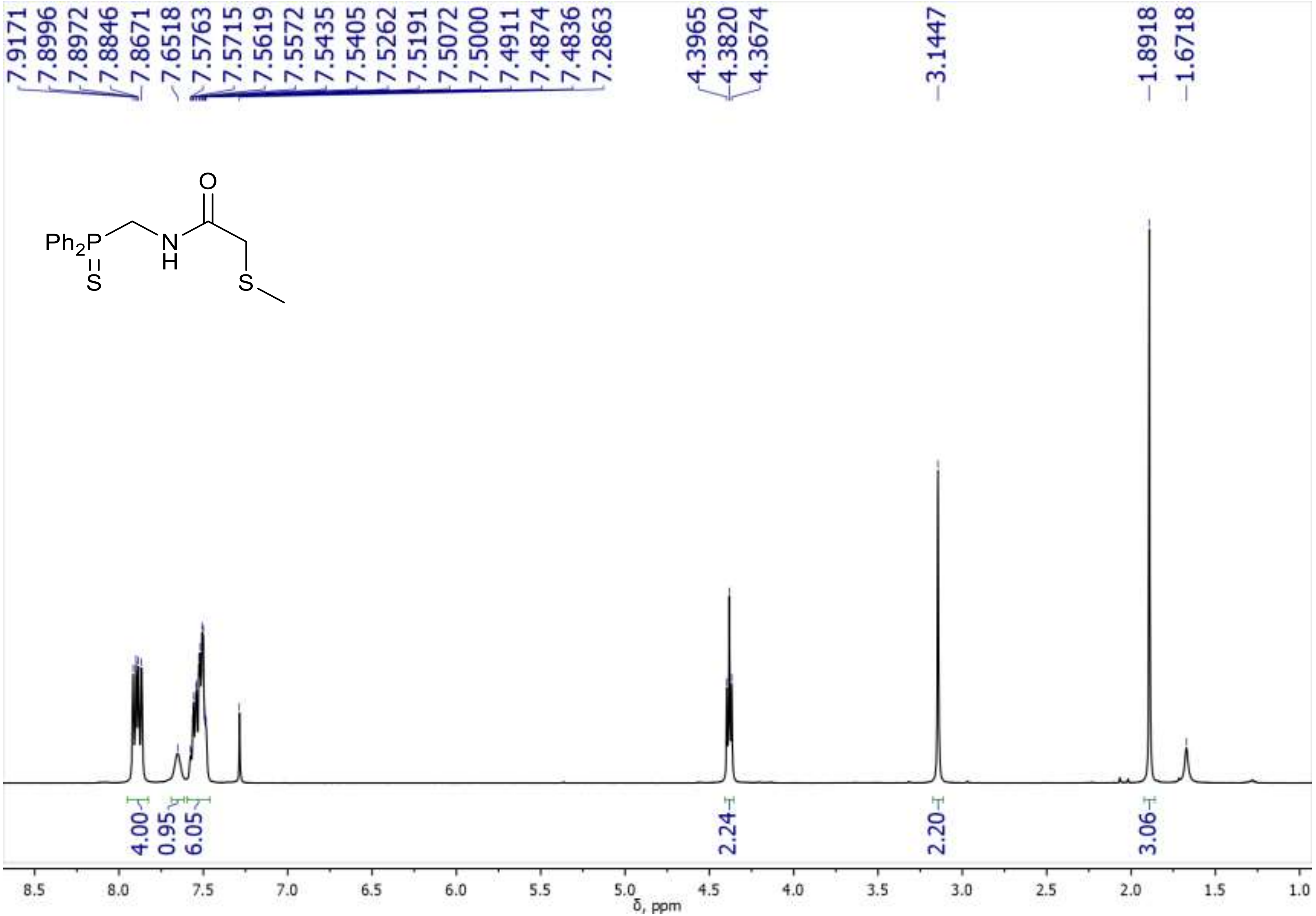

Figure S8. ^{1}H NMR spectrum of ligand **7b** (400.13 MHz, $CDCl_3$)

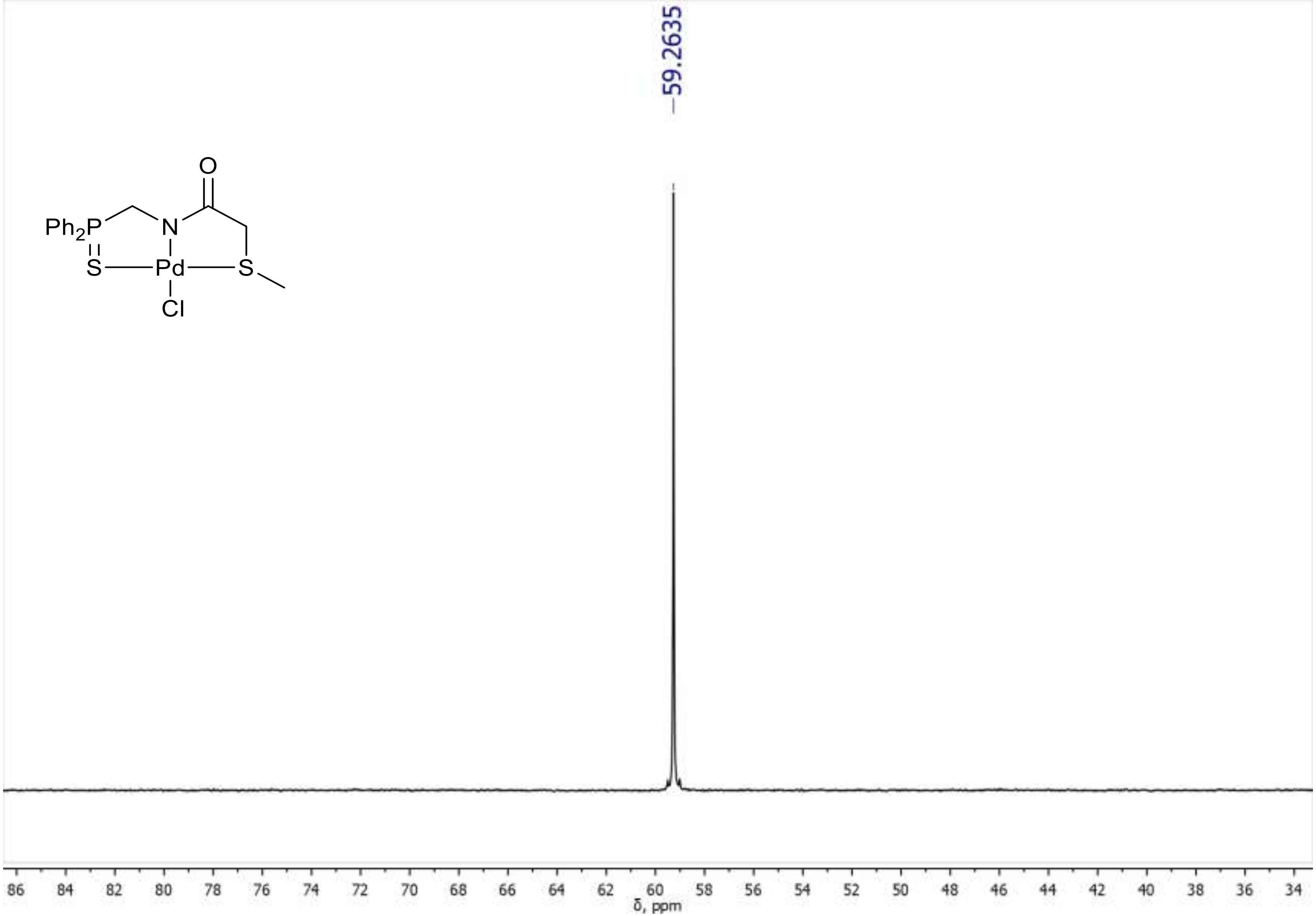

Figure S9. $^{31}P\{^{1}H\}$ NMR spectrum of complex **13b** (161.98 MHz, $CDCl_3$)

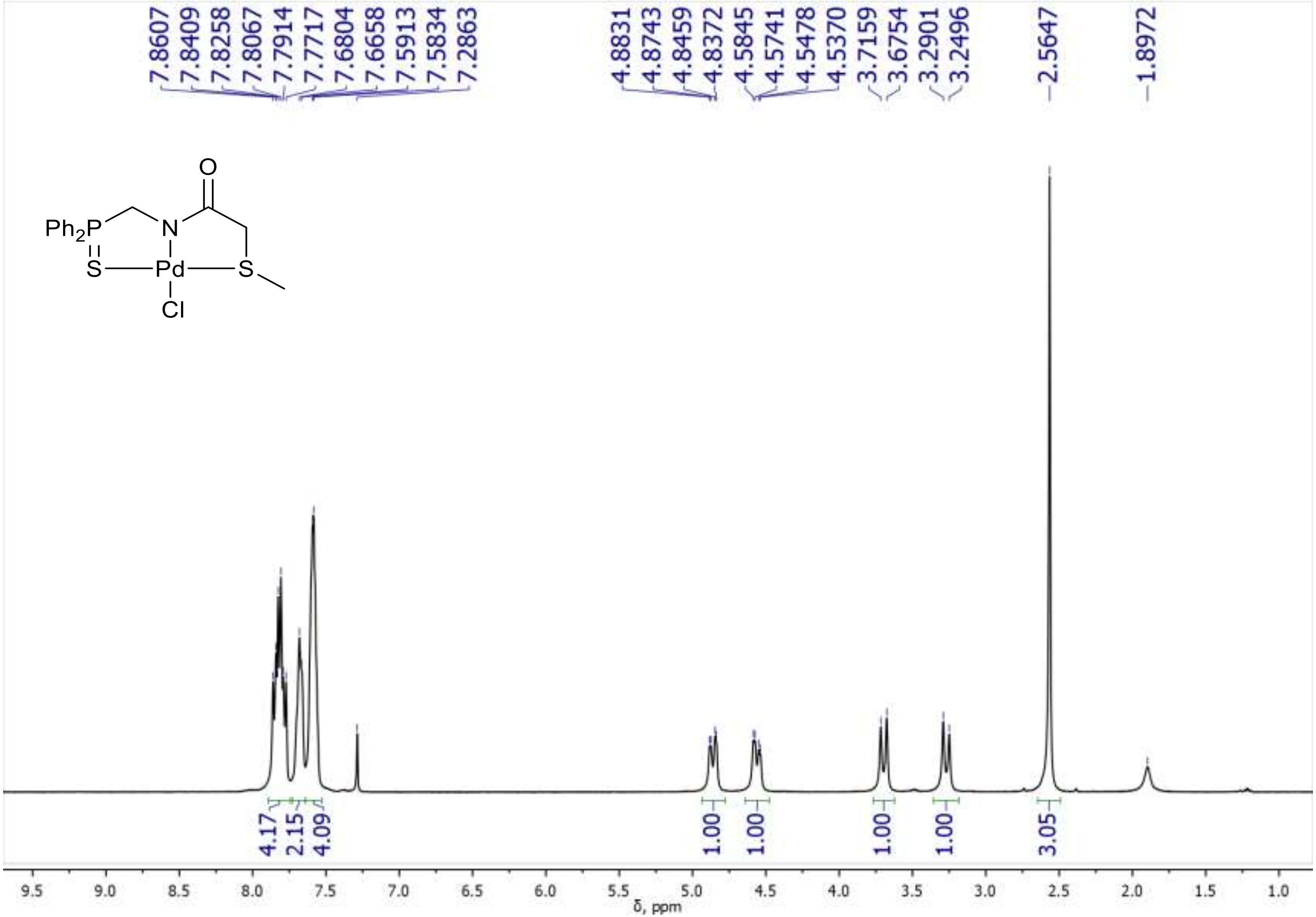

Figure S10. ^{1}H NMR spectrum of complex **13b** (400.13 MHz, $CDCl_3$)

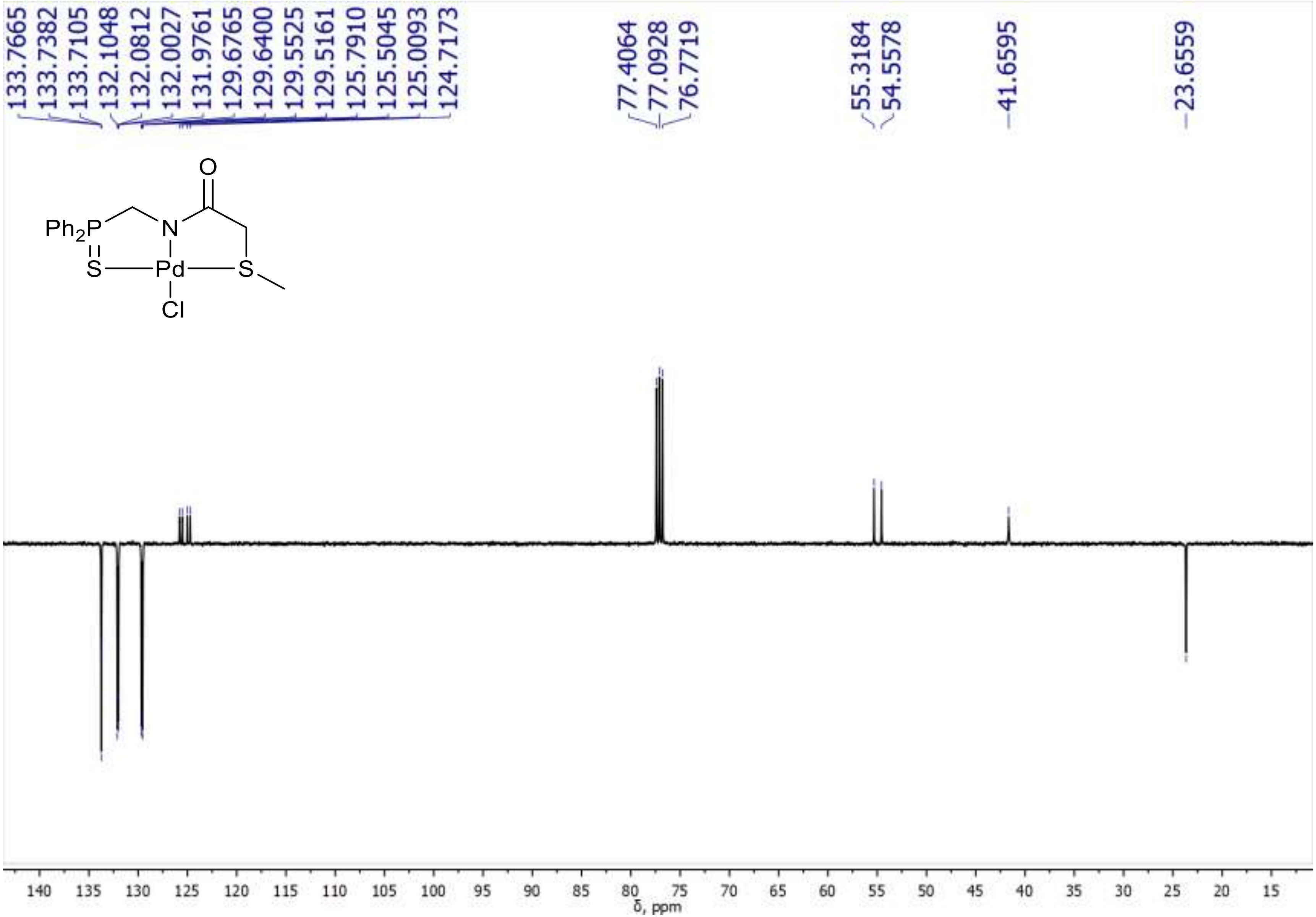

Figure S11. $^{13}C\{^1H\}$ spectrum of complex **13b** (100.61 MHz, $CDCl_3$)

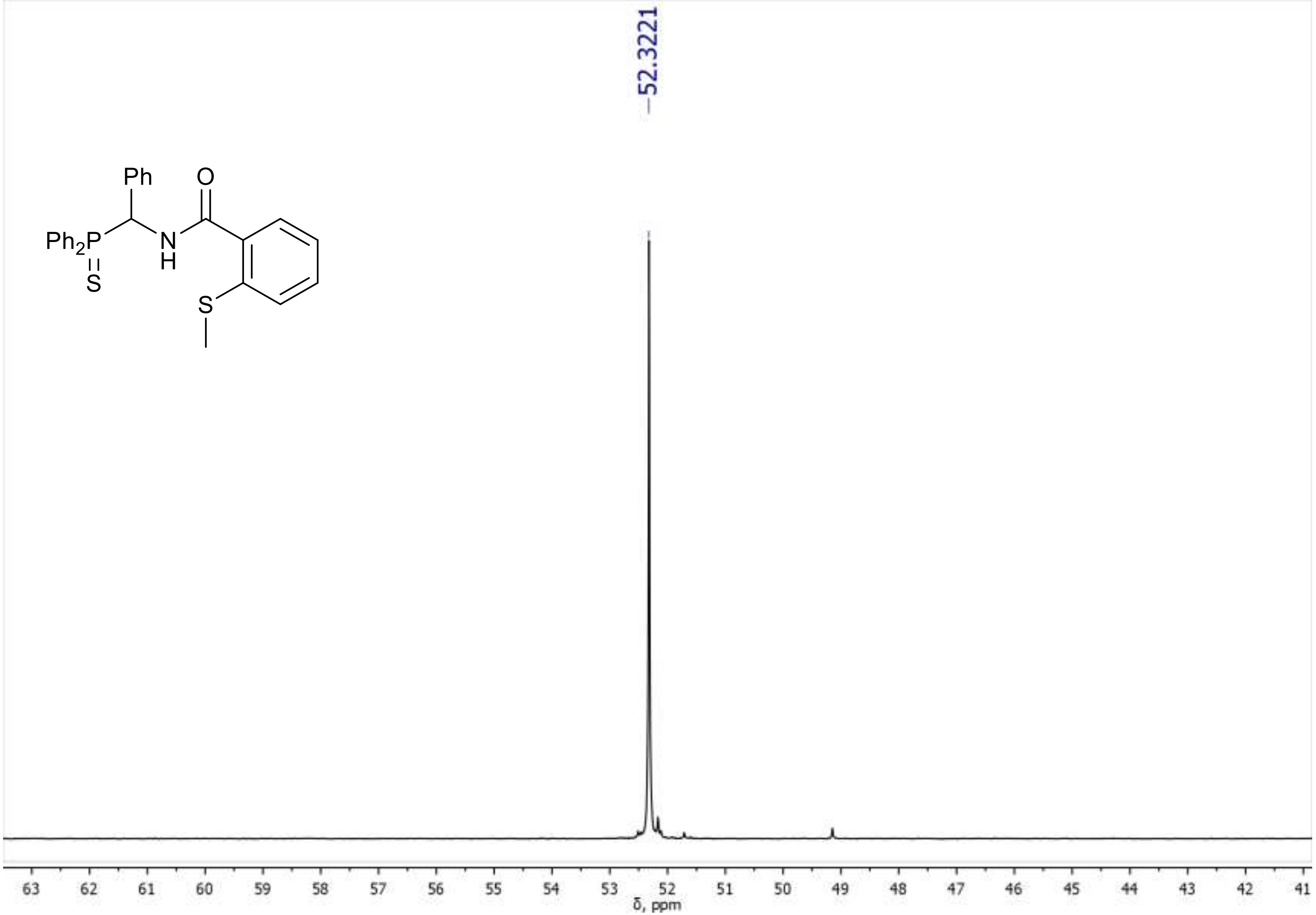

Figure S12. $^{31}P\{^1H\}$ NMR spectrum of ligand **8** (202.45 MHz, $CDCl_3$)

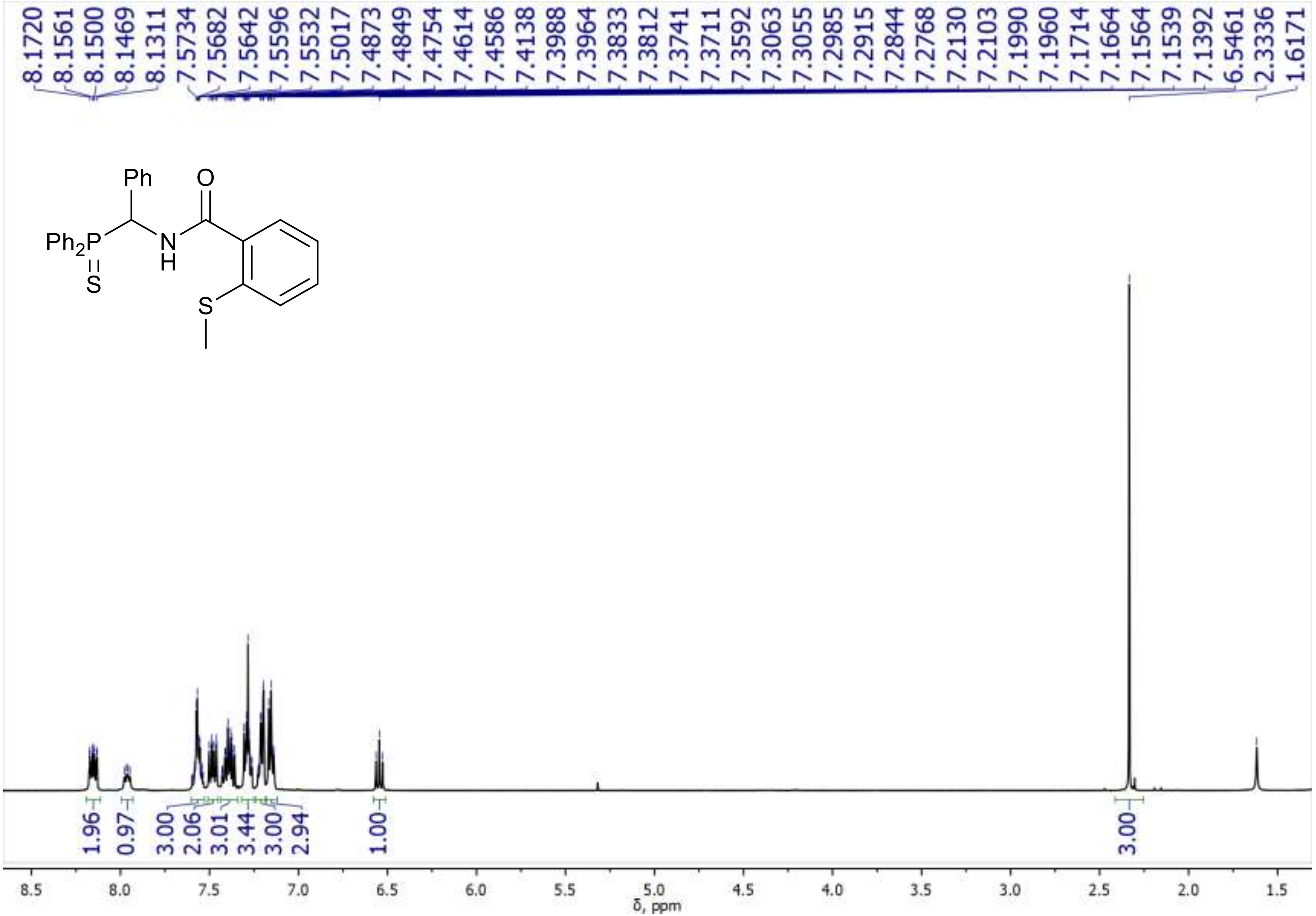

Figure S13. ^{1}H NMR spectrum of ligand **8** (500.13 MHz, $CDCl_3$)

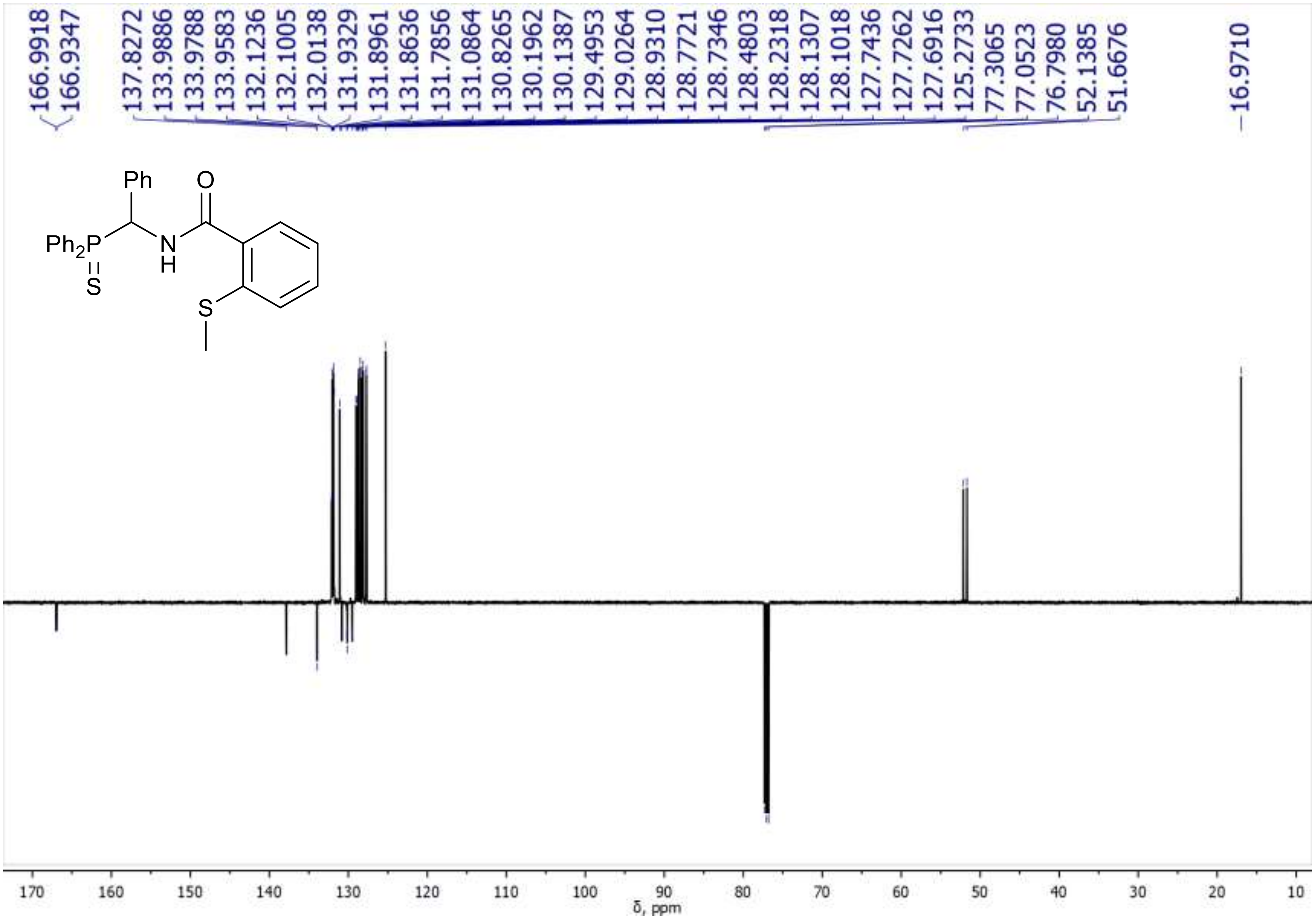

Figure S14. $^{13}C\{^1H\}$ spectrum of ligand **8** (125.76 MHz, $CDCl_3$)

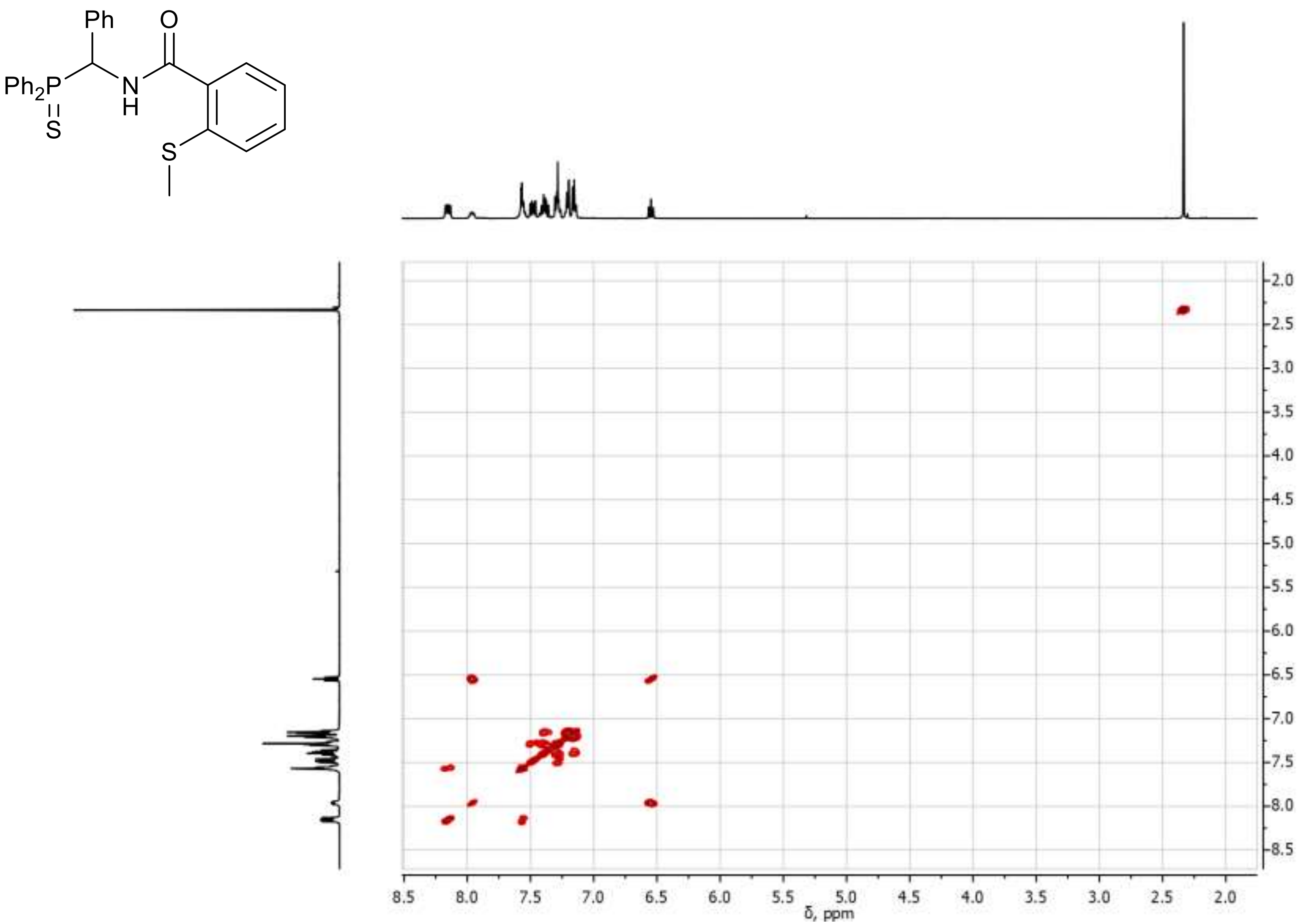

Figure S15. ^{1}H–^{1}H COSY spectrum of ligand **8** (500.13 MHz, $CDCl_3$)

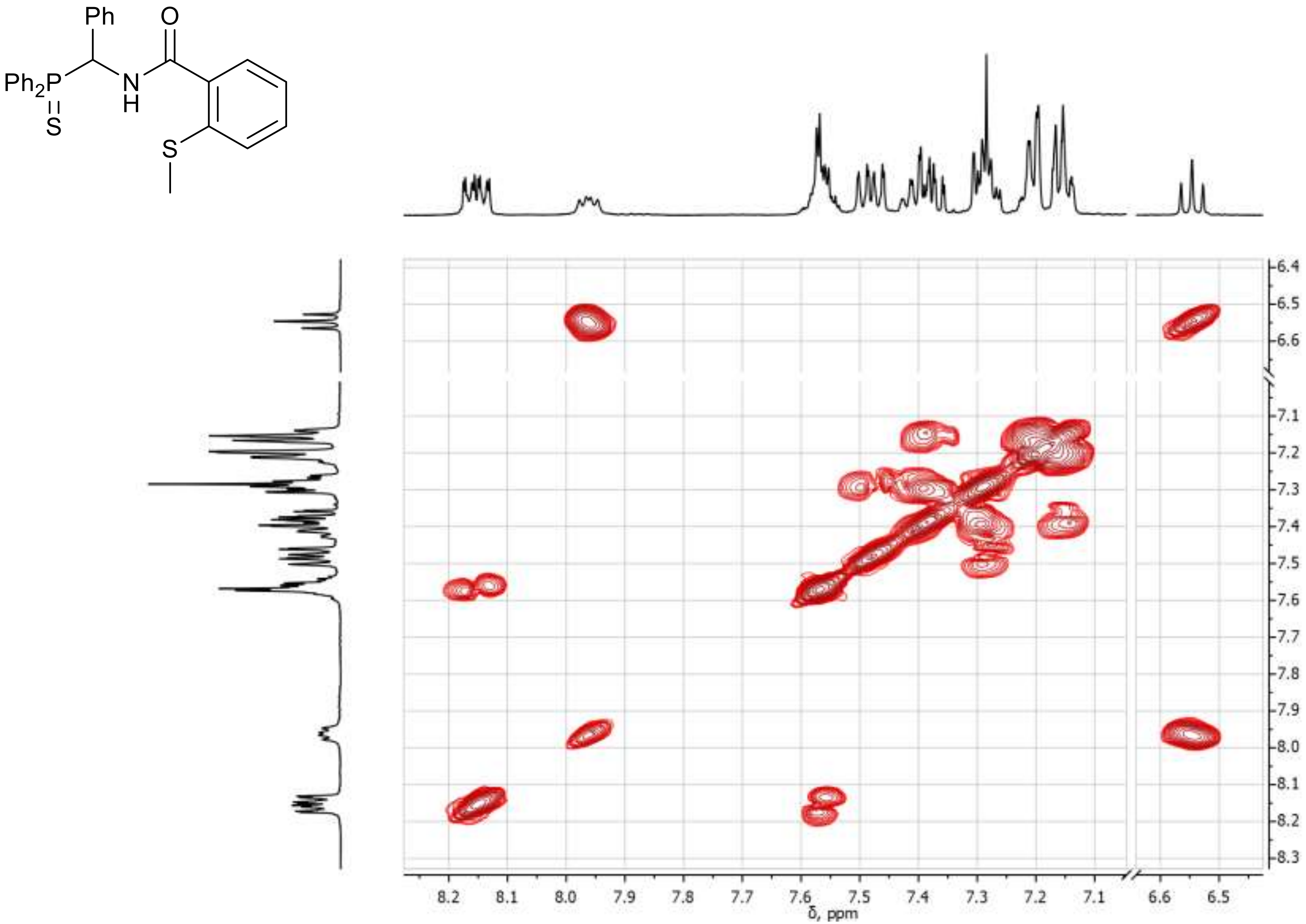

Figure S16. Extended fragments of the $^1H{-}^1H$ COSY spectrum of ligand **8** (500.13 MHz, $CDCl_3$)

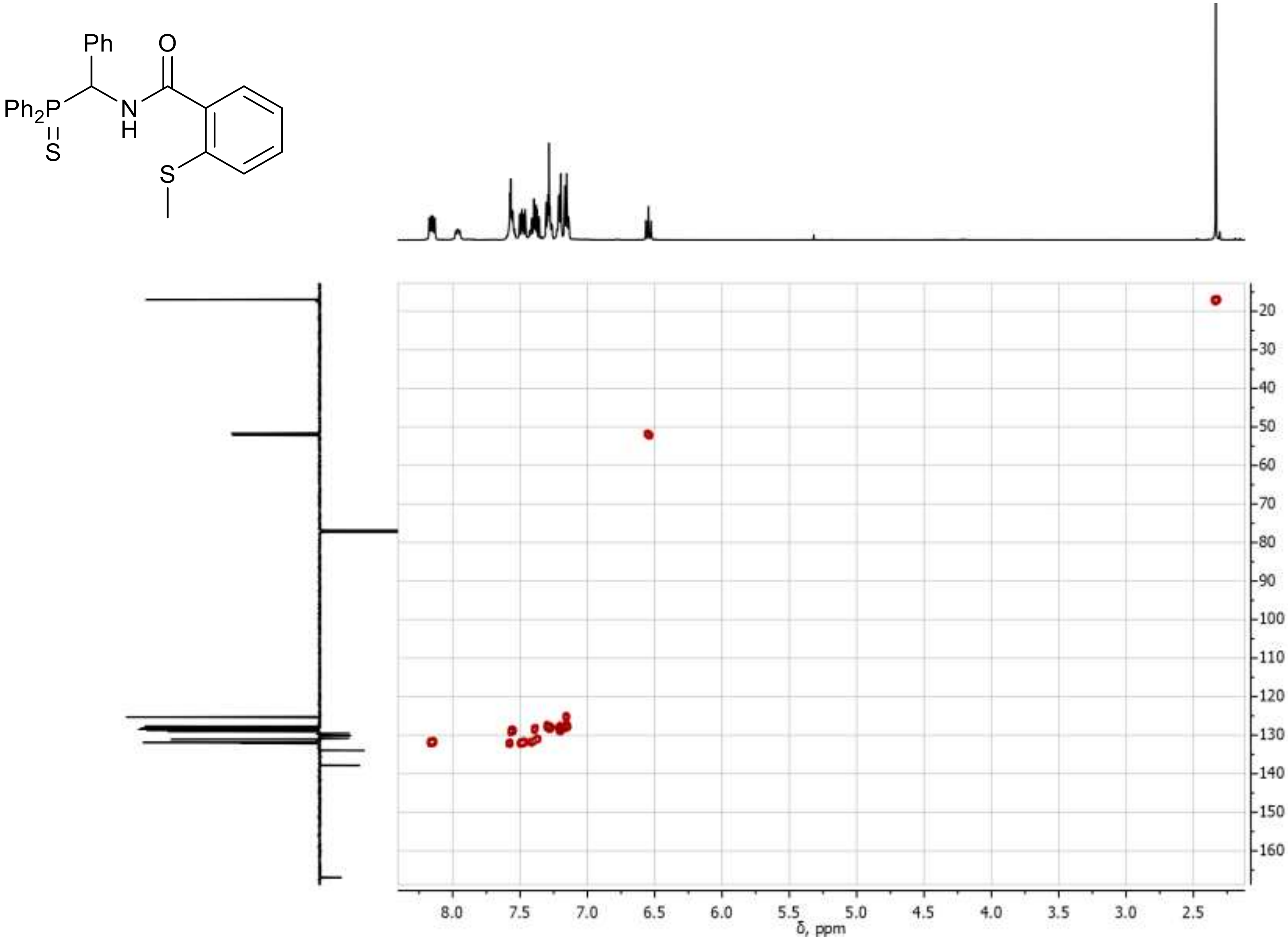

Figure S17. HMQC spectrum of ligand **8** ($CDCl_3$)

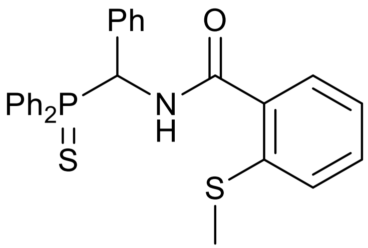

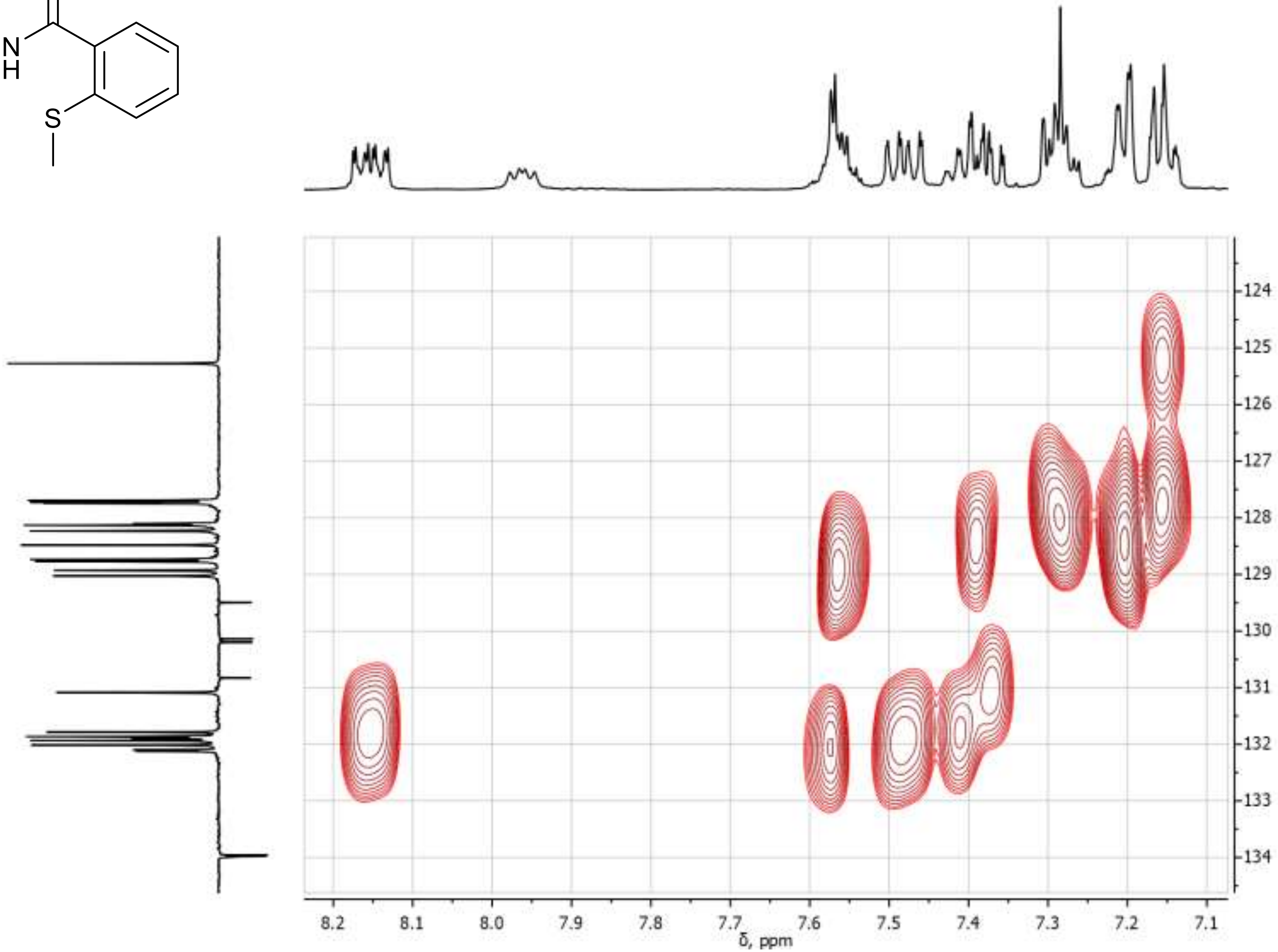

Figure S18. Extended fragment of the HMQC spectrum of ligand **8** ($CDCl_3$)

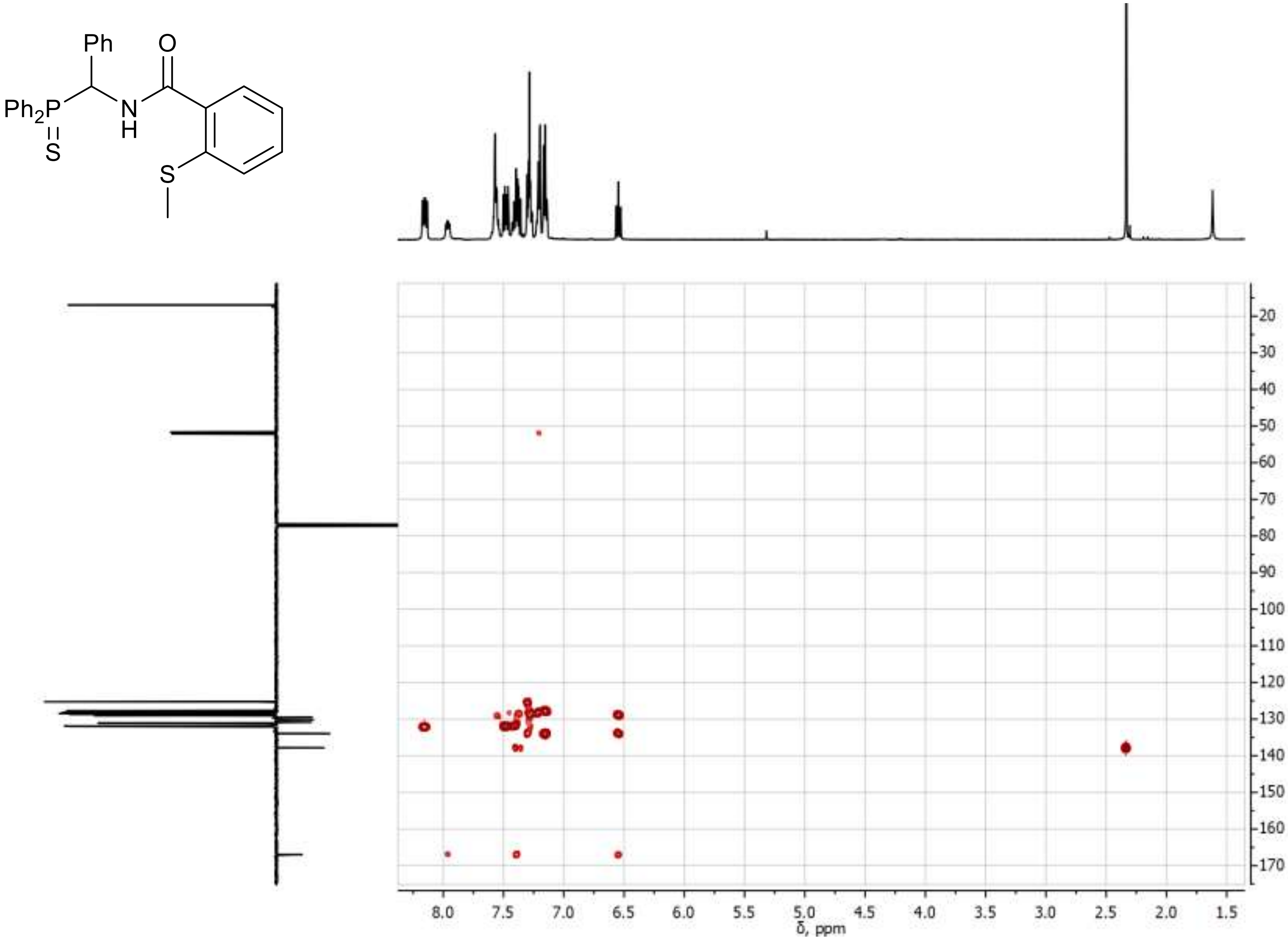

Figure S19. ^{1}H–^{13}C HMBC spectrum of ligand **8** ($CDCl_3$)

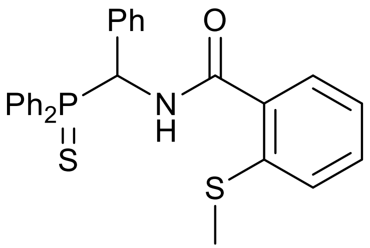

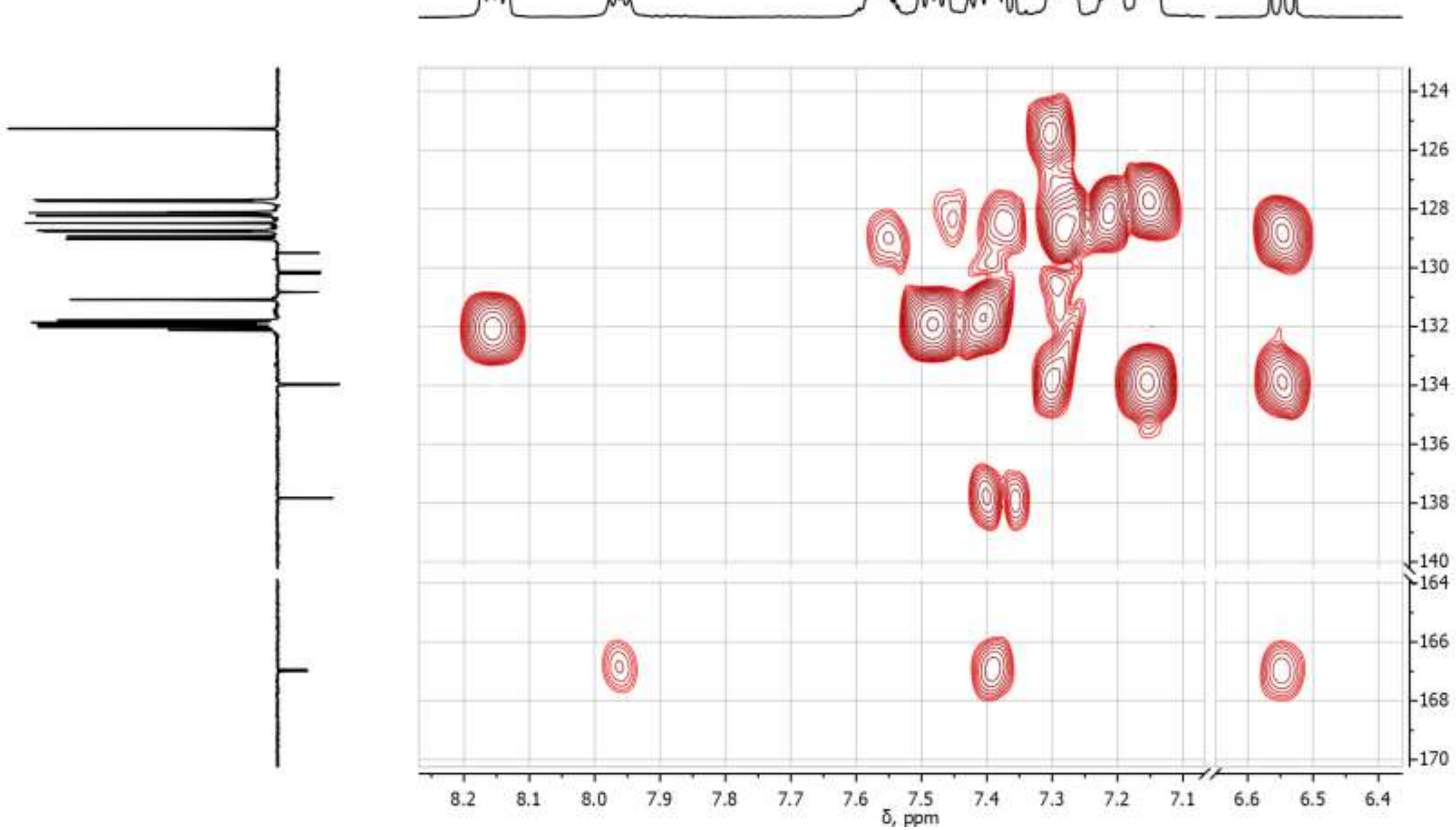

Figure S20. Extended fragments of the $^{1}H-^{13}C$ HMBC spectrum of ligand **8** ($CDCl_3$)

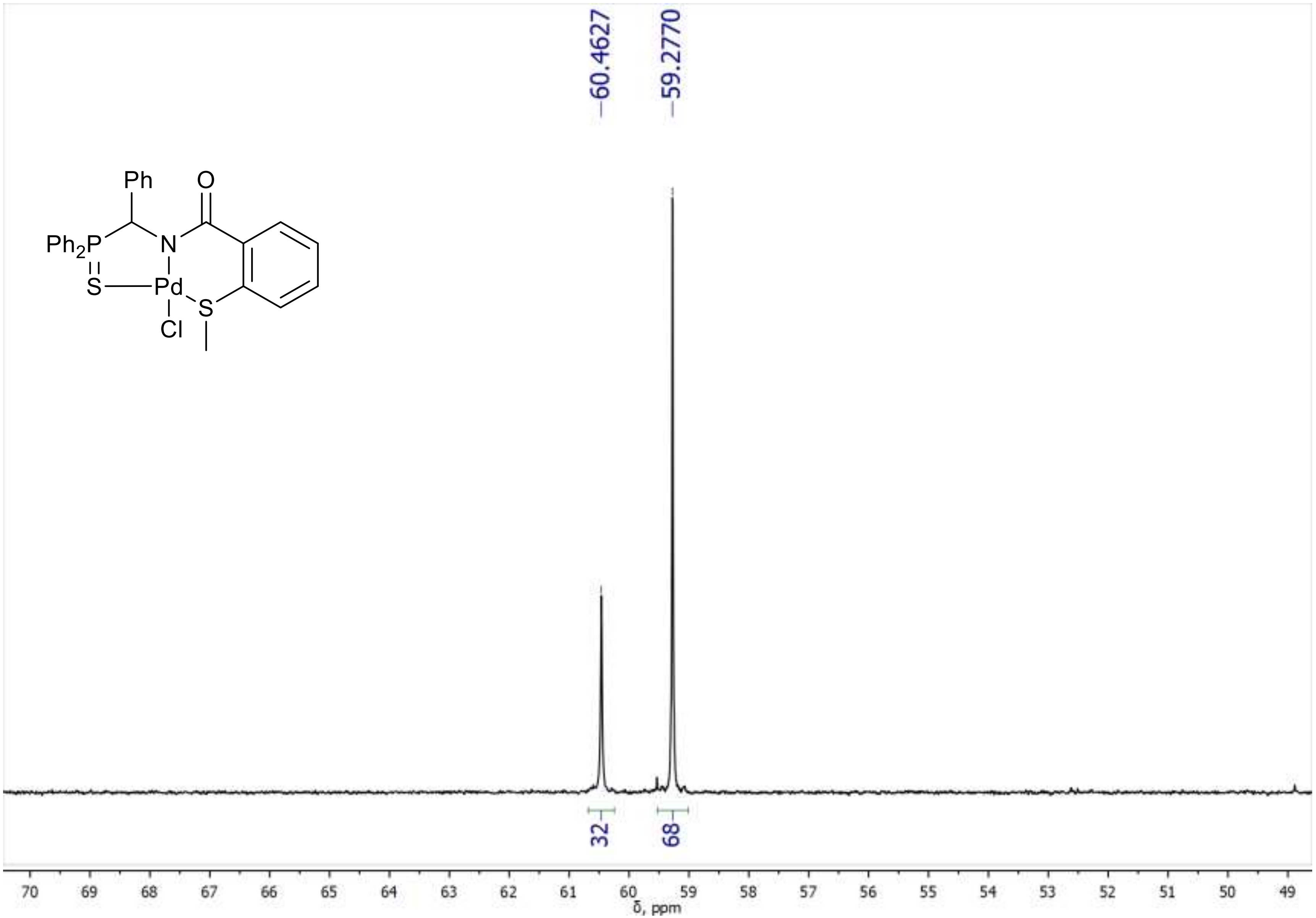

Figure S21. $^{31}P\{^1H\}$ NMR spectrum of complex **14** (202.45 MHz, $CDCl_3$, 258 K)

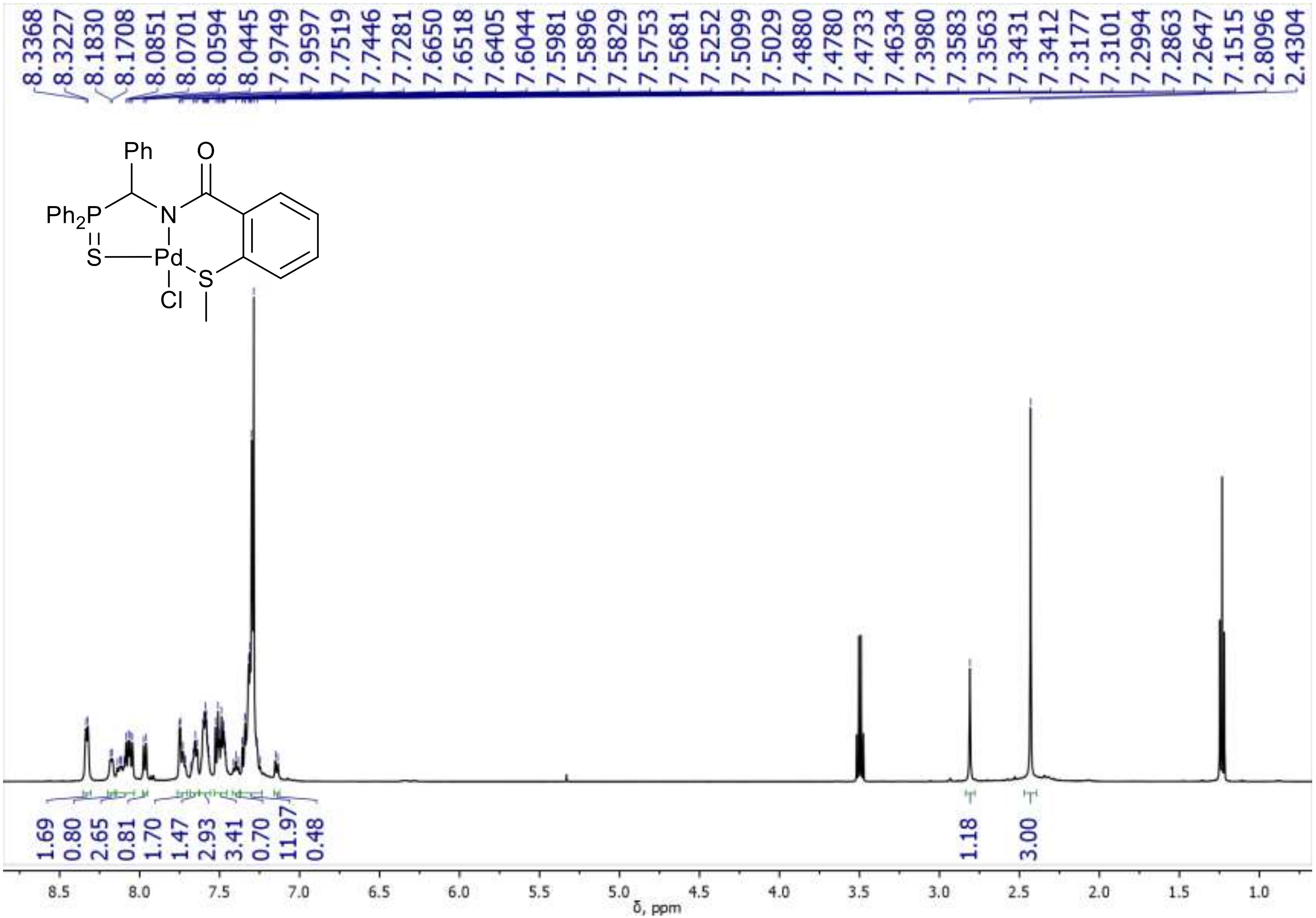

Figure S22. ^1H NMR spectrum of complex **14** (500.13 MHz, $CDCl_3$, 258 K)

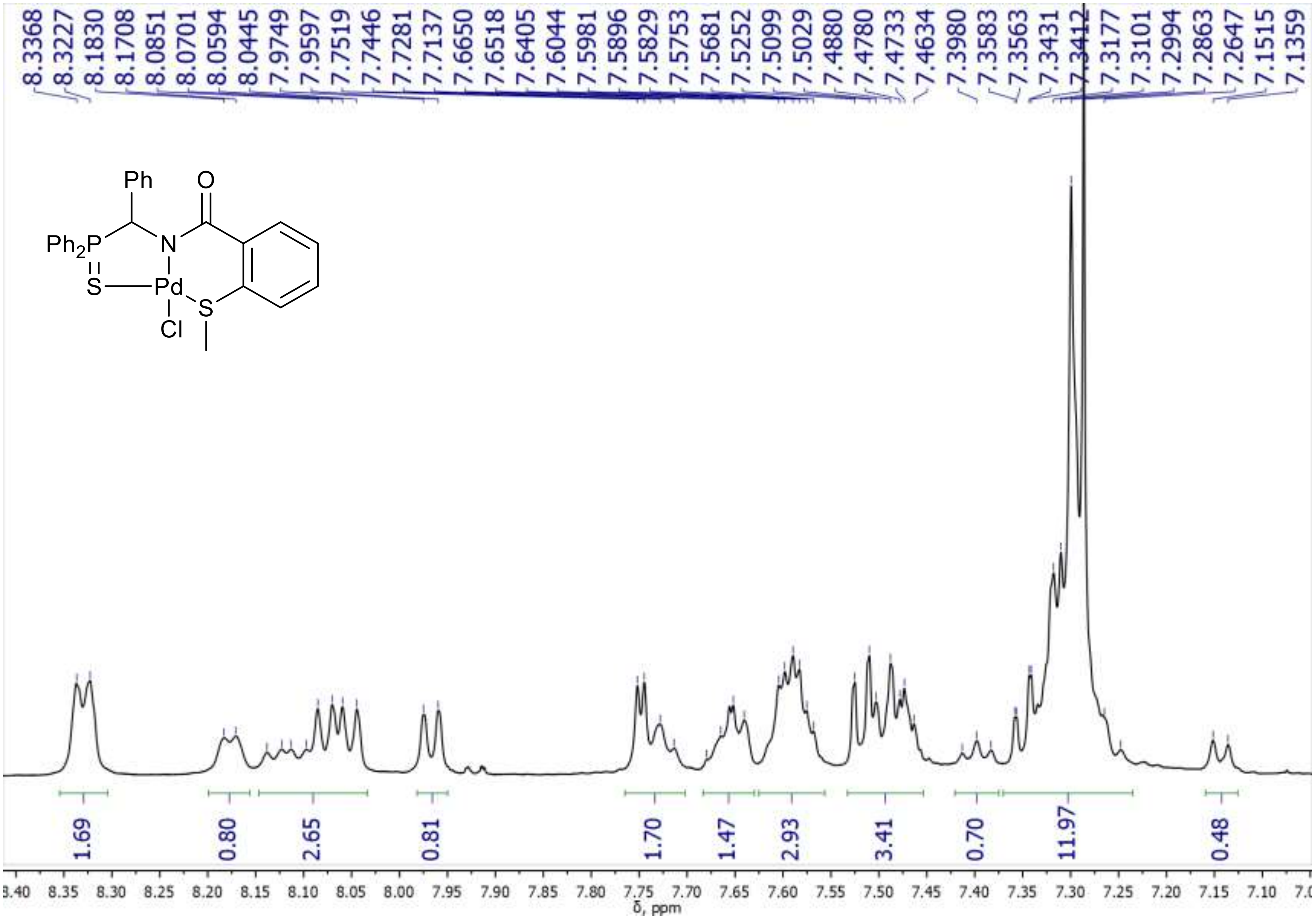

Figure S23. Extended fragment of the ^{1}H NMR spectrum of complex **14** (500.13 MHz, $CDCl_3$, 258 K)

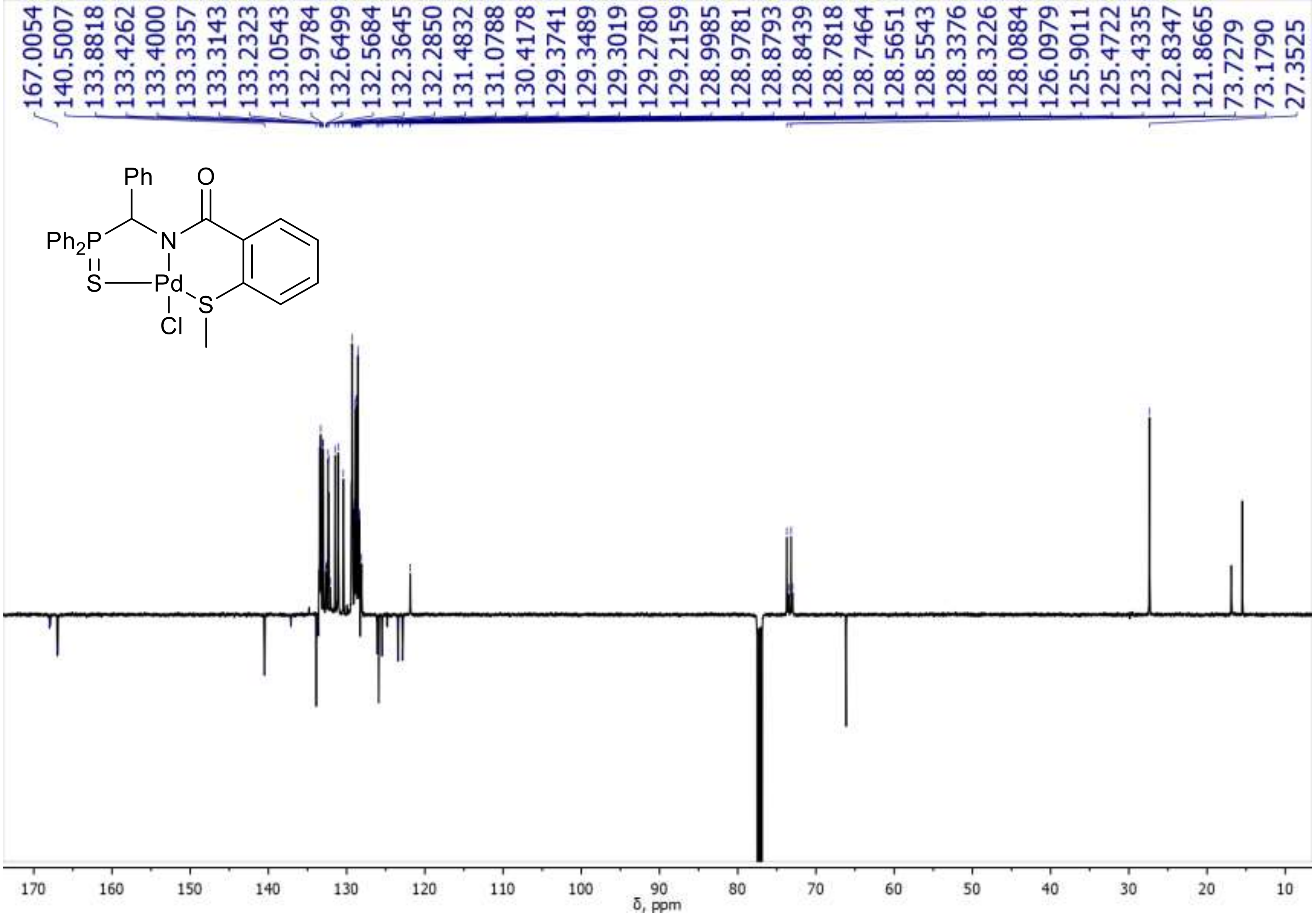

Figure S24. $^{13}C\{^1H\}$ spectrum of complex **14** (125.76 MHz, $CDCl_3$, 258 K)

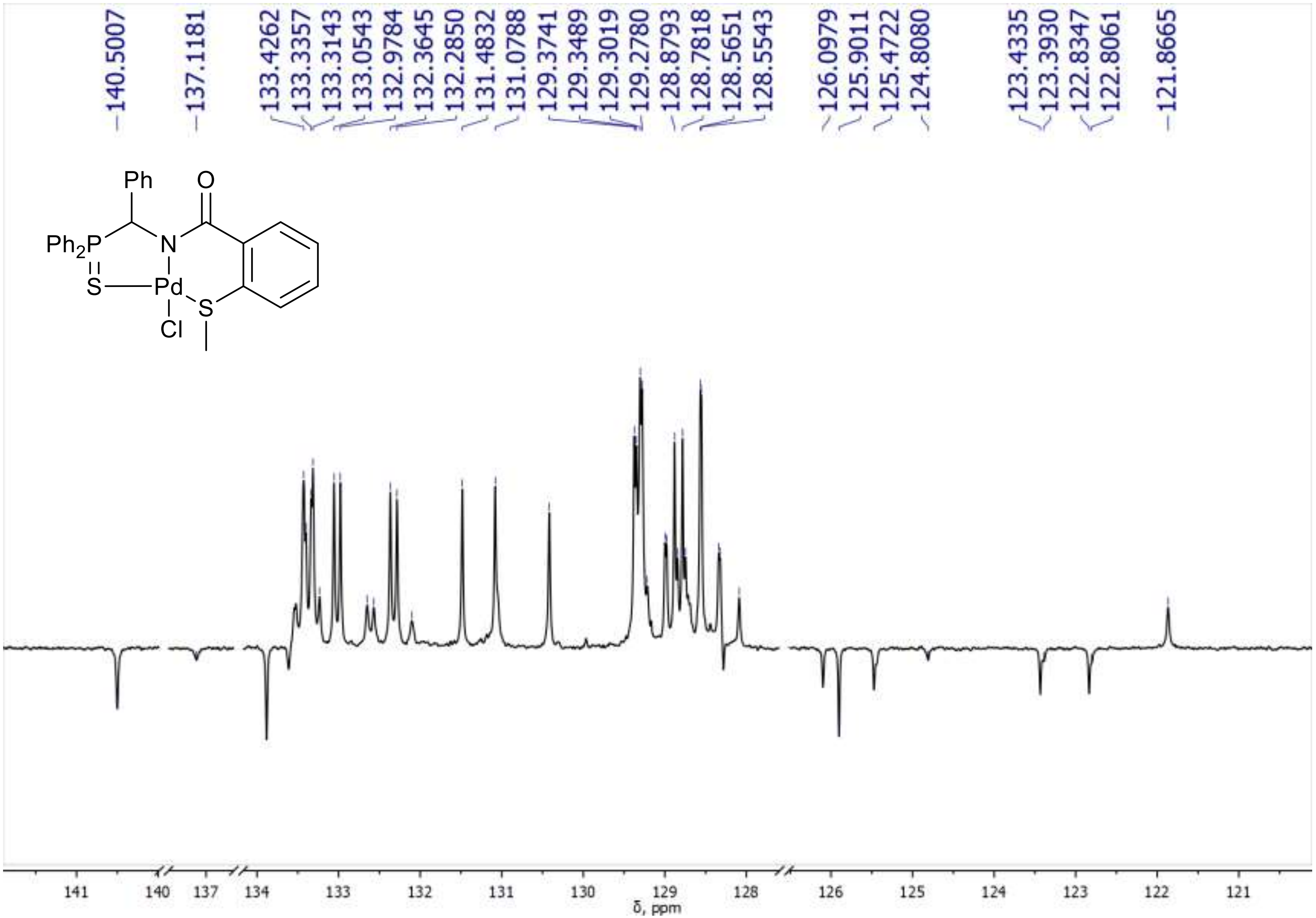

Figure S25. Extended fragments of the $^{13}C\{^{1}H\}$ spectrum of complex **14** (125.76 MHz, $CDCl_3$, 258 K)

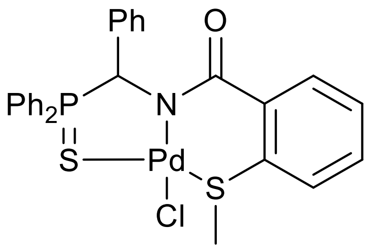

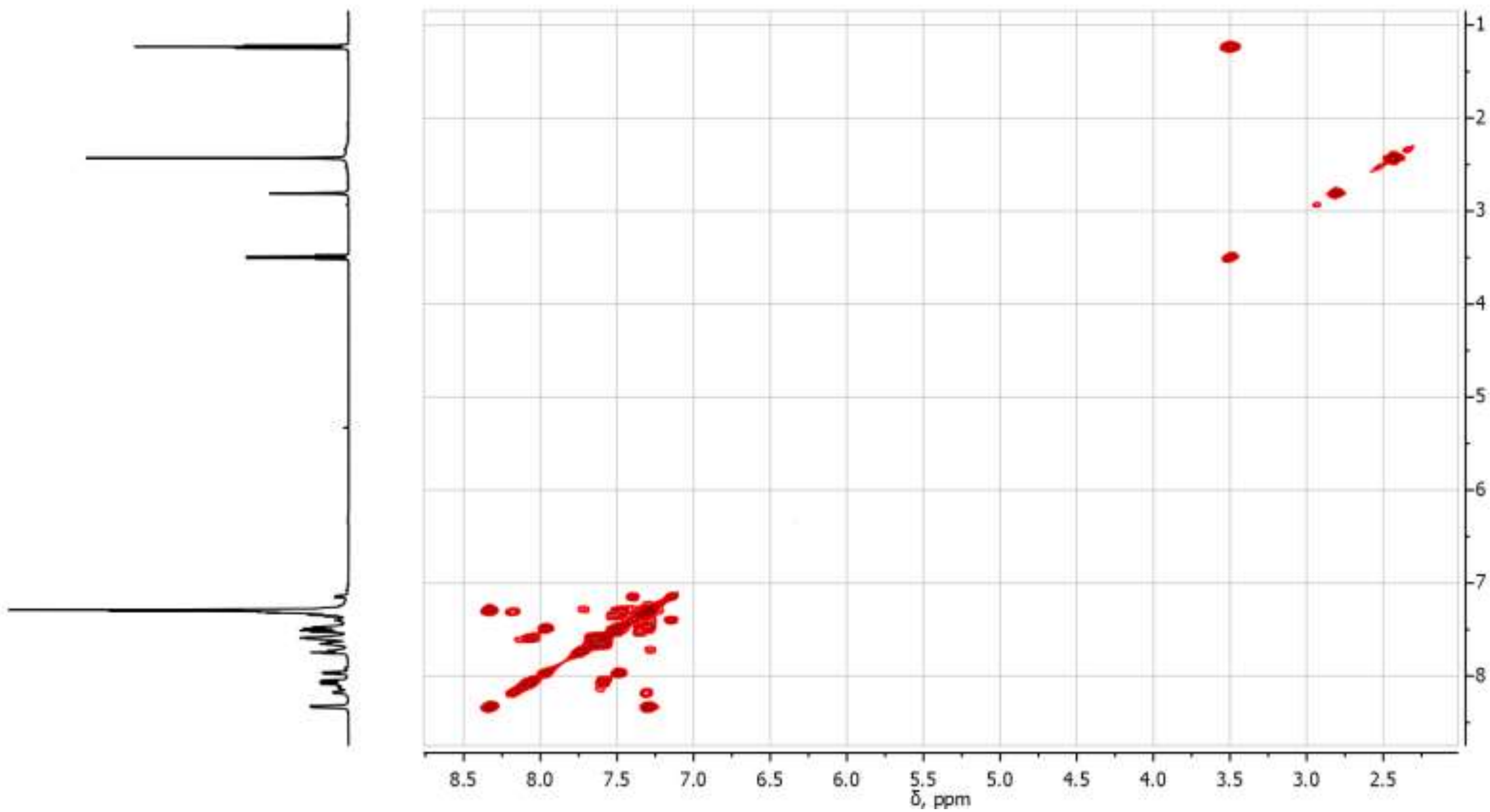

Figure S26. $^1H-^1H$ COSY spectrum of complex **14** (500.13 MHz, $CDCl_3$, 258 K)

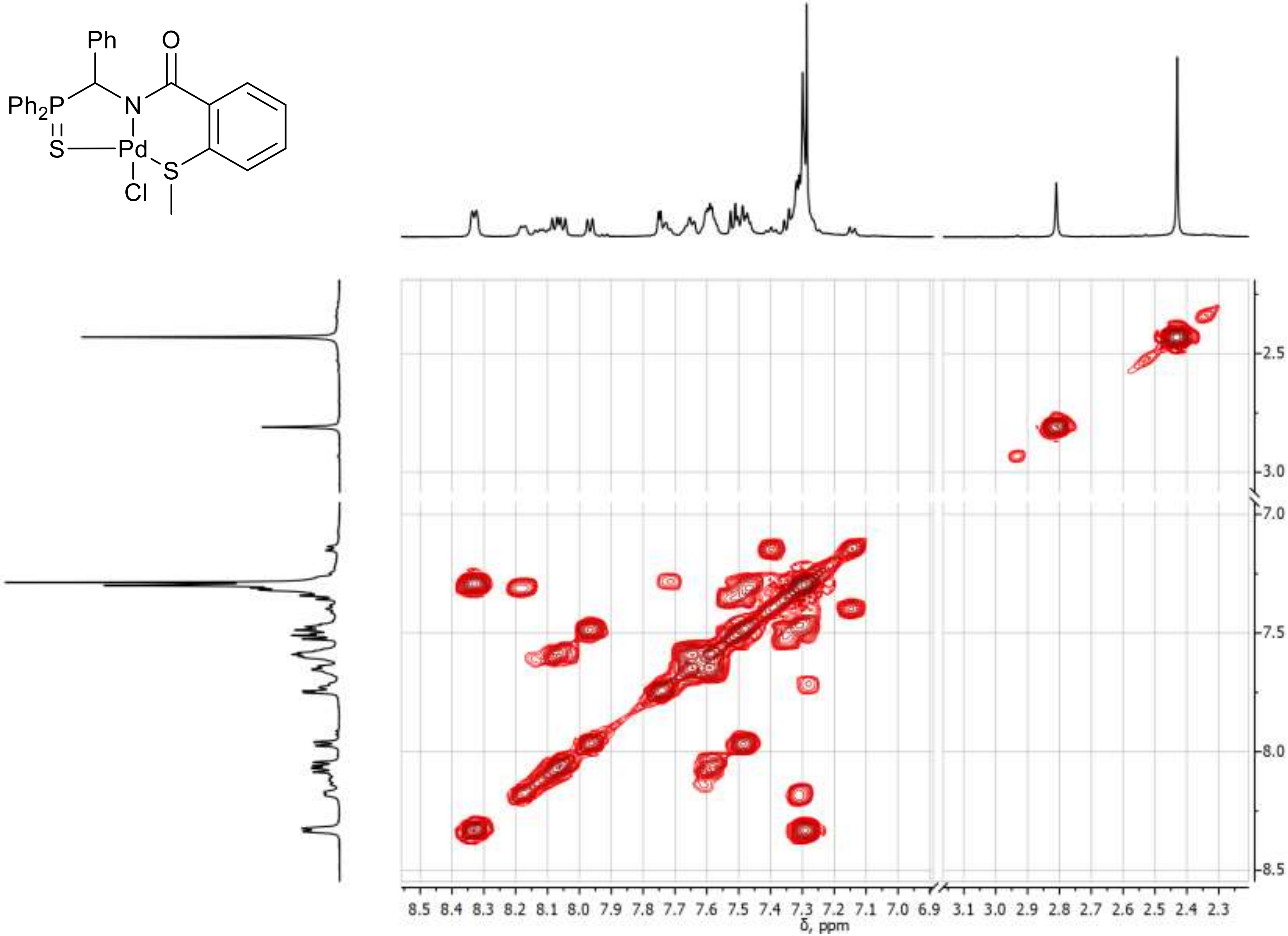

Figure S27. Extended fragments of the $^1H-^1H$ COSY spectrum of complex **14** (500.13 MHz, $CDCl_3$, 258 K)

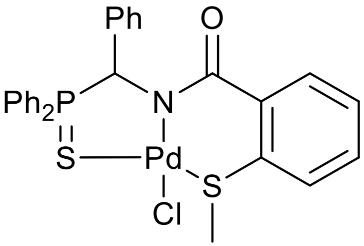

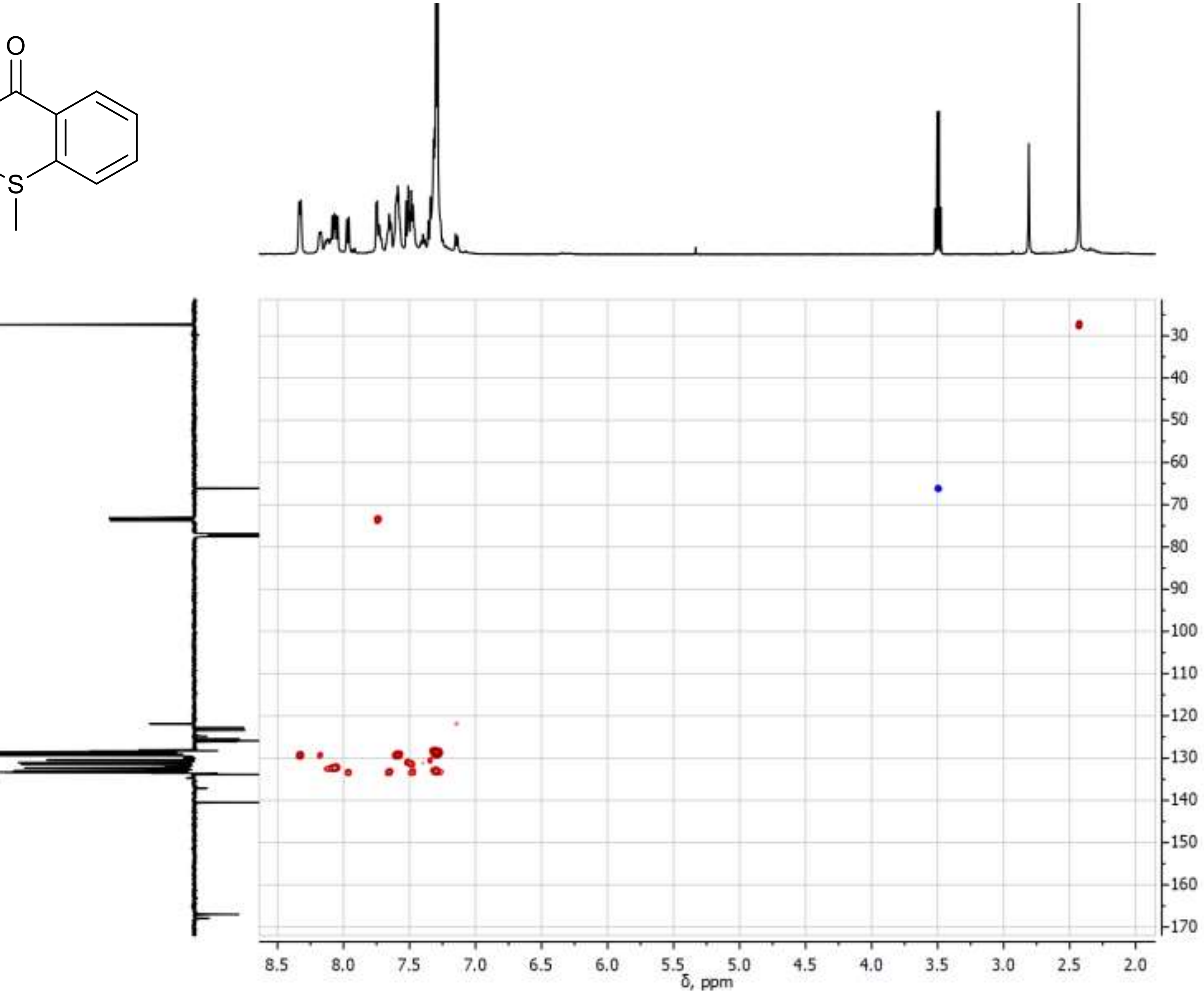

Figure S28. HSQC spectrum of complex **14** ($CDCl_3$, 258 K)

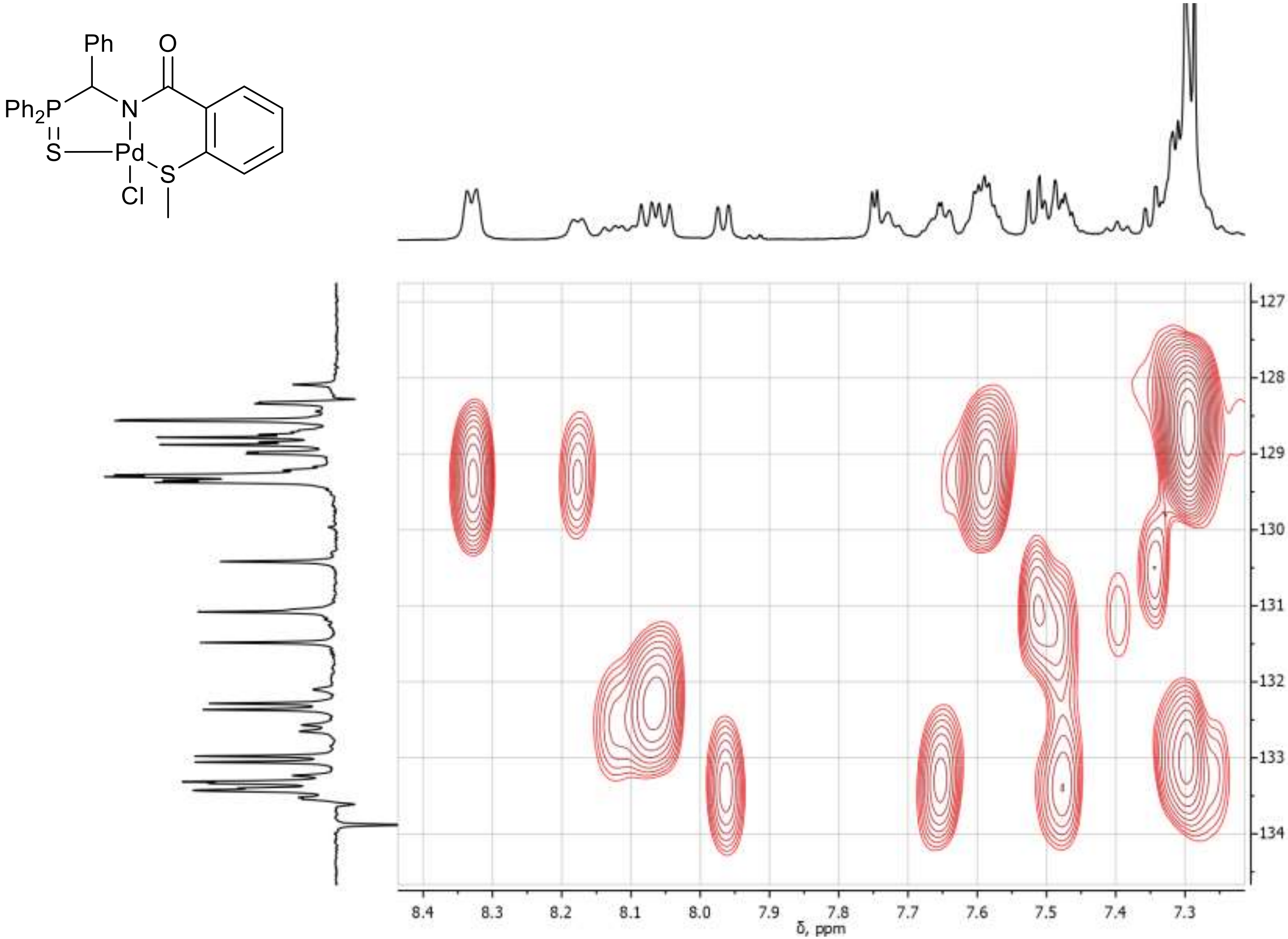

Figure S29. Extended fragment of the HSQC spectrum of complex **14** ($CDCl_3$, 258 K)

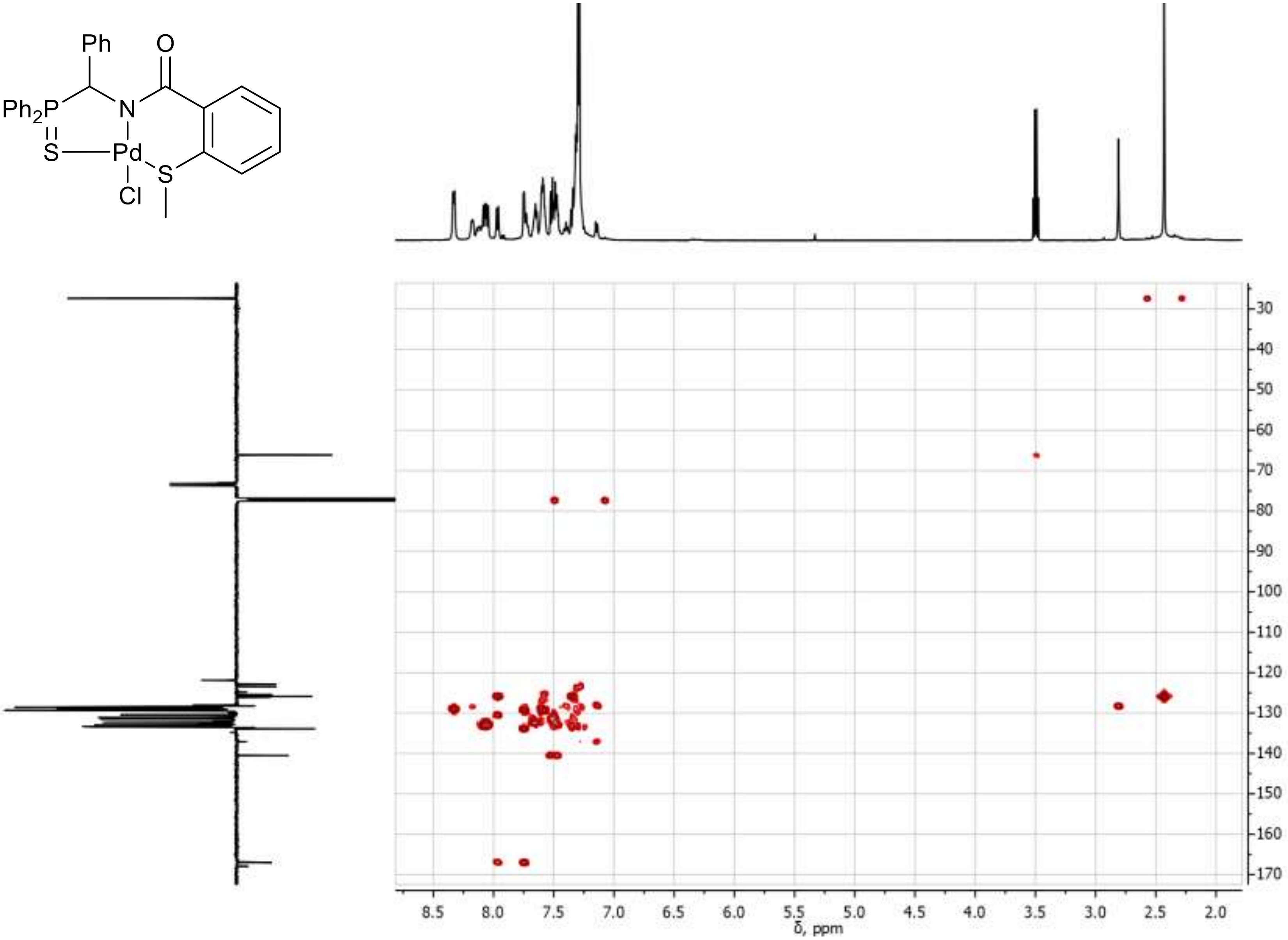

Figure S30. ^{1}H−^{13}C HMBC spectrum of complex **14** ($CDCl_3$, 258 K)

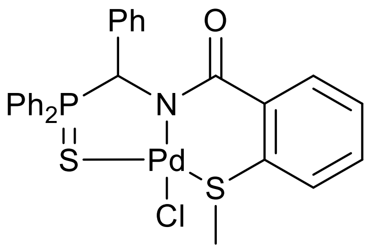

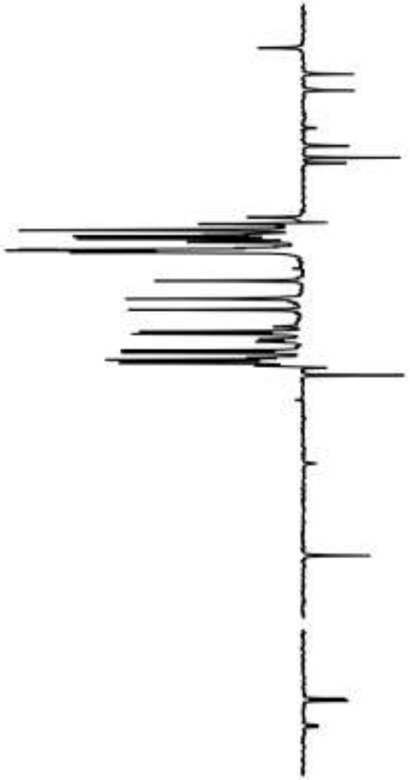

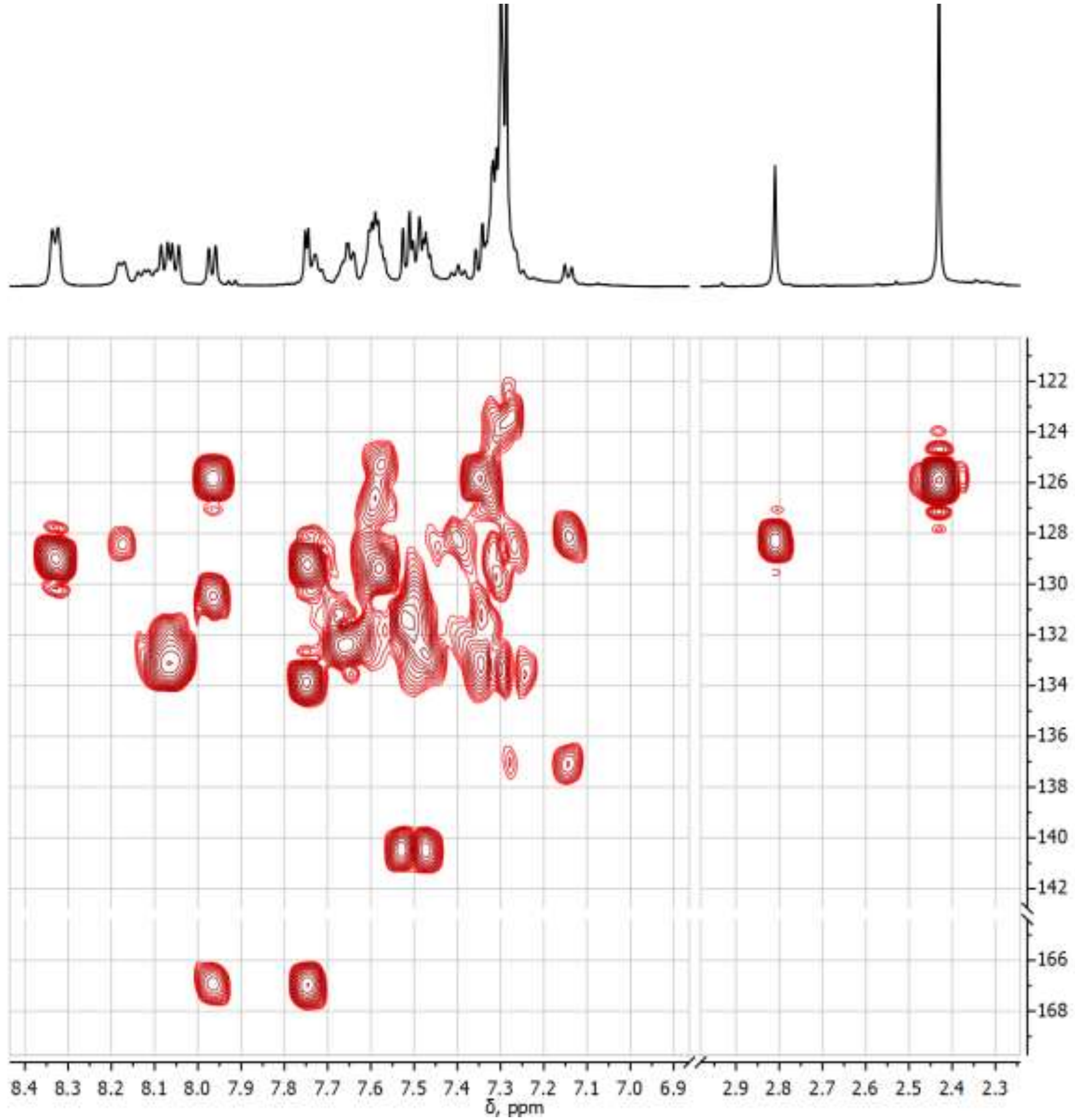

Figure S31. Extended fragments of the $^{1}H-^{13}C$ HMBC spectrum of complex **14** ($CDCl_3$, 258 K)

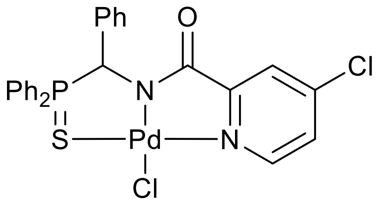

9

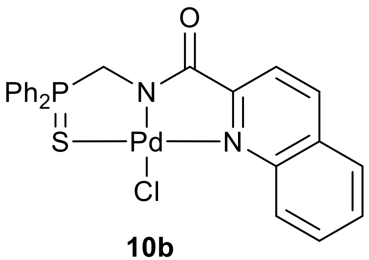

10b

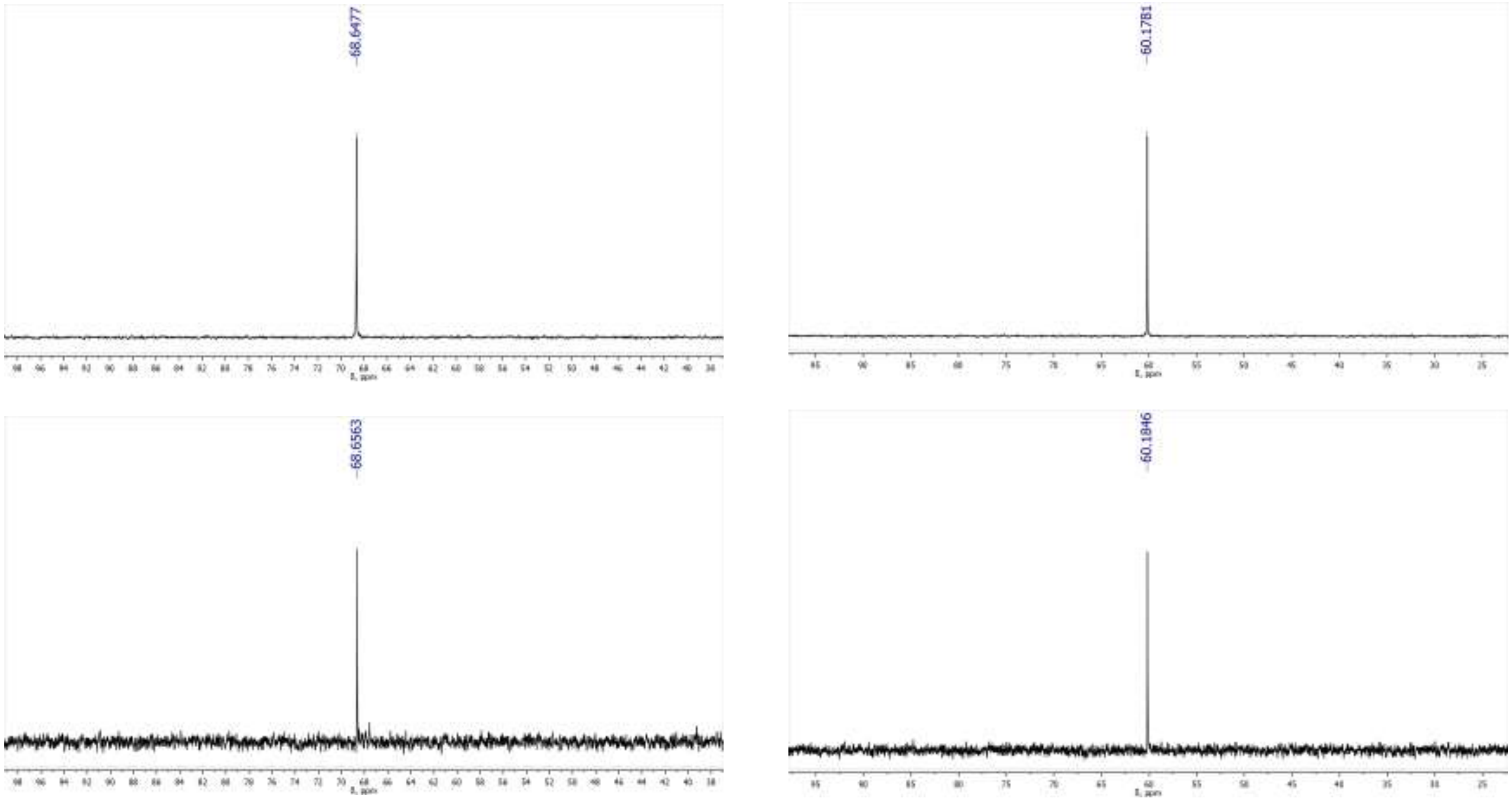

Figure S32. ^{31}P NMR spectra of the solutions of complexes **9** (left) and **10b** (right) in 1 h (top) or 3 days (bottom) after dissolution in $(CD_3)_2SO$ (161.98 MHz)

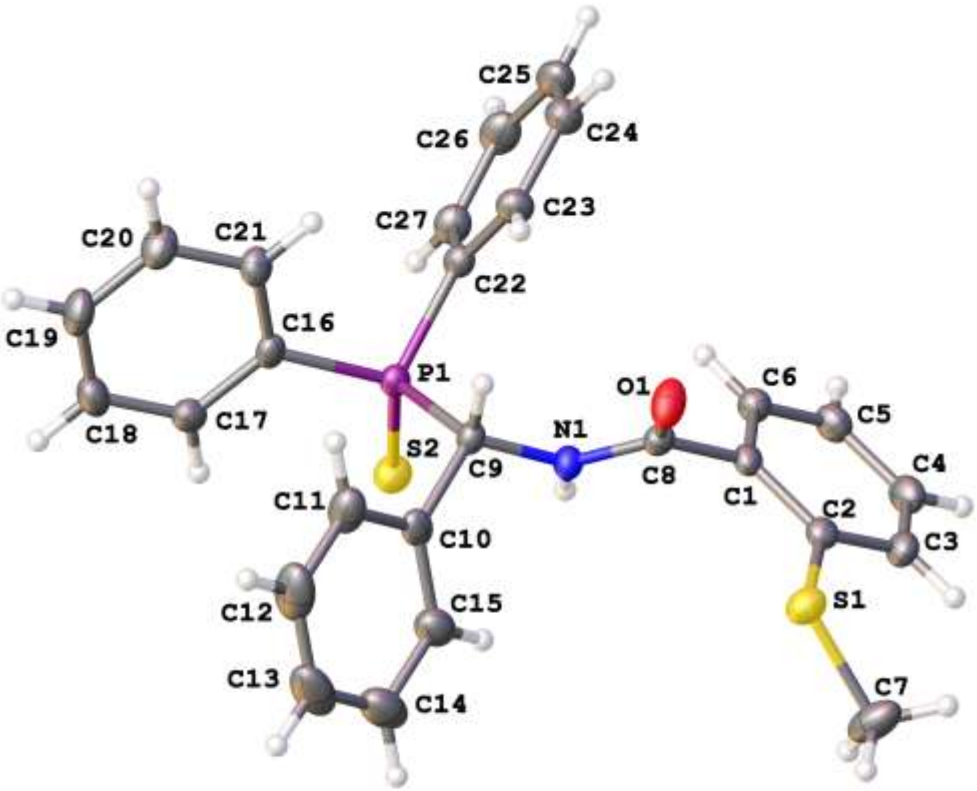

Figure S33. General view of ligand **8**

Hereinafter, the non-hydrogen atoms are drawn as thermal ellipsoids at 50% probability level.

Table S1. Main bond lengths (Å) and angles (°) for the complexes explored

	**9**	**10a**	**10b**	**11**	**12**	**13a**	**13b**	***R*-18**
Pd–S(P)	2.2990(4)	2.2884(9)	2.2939(6)	2.3135(5)	2.2696(5)	2.3347(6)	2.3490(9)	2.3137(9)
Pd–N(amide)	1.9961(13)	1.990(3)	1.9926(17)	1.9998(14)	2.0071(16)	2.0152(16)	2.005(3)	1.998(3)
Pd–Cl	2.3018(4)	2.3074(9)	2.3460(5)	2.3253(4)	2.3067(5)	2.3127(6)	2.3051(9)	2.3076(9)
Pd–X[a]	2.0359(13)	2.090(3)	2.1310(17)	2.0621(15)	2.0763(17)	2.2905(6)	2.2872(9)	2.038(3)
P–S	2.0163(6)	2.0149(12)	2.0148(7)	2.0209(6)	2.0185(7)	2.0236(7)	2.0147(12)	2.0220(13)
Cl–Pd–N(amide)	176.48(4)	176.46(8)	173.78(5)	177.16(4)	175.53(5)	176.94(5)	175.70(8)	176.06(9)
X–Pd–S(P)[a]	172.42(4)	168.75(7)	171.27(5)	175.19(4)	172.20(5)	176.64(2)	176.91(3)	170.68(10)
(P)S–Pd–N(amide)	92.03(4)	91.62(8)	91.34(5)	92.27(4)	91.56(5)	92.52(5)	93.15(8)	91.47(9)
X–Pd–N(amide)[a]	80.51(5)	80.11(10)	79.94(7)	89.62(4)	80.66(6)	85.44(5)	84.98(8)	81.13(13)
X–Pd–Cl[a]	96.00(4)	102.43(8)	106.19(5)	93.09(4)	100.41(5)	91.89(2)	91.57(3)	95.39(10)
Cl–Pd–S(P)	91.474(14)	85.53(3)	82.535(19)	85.097(16)	87.387(18)	90.08(2)	90.18(3)	92.17(3)

[a] X = N or S

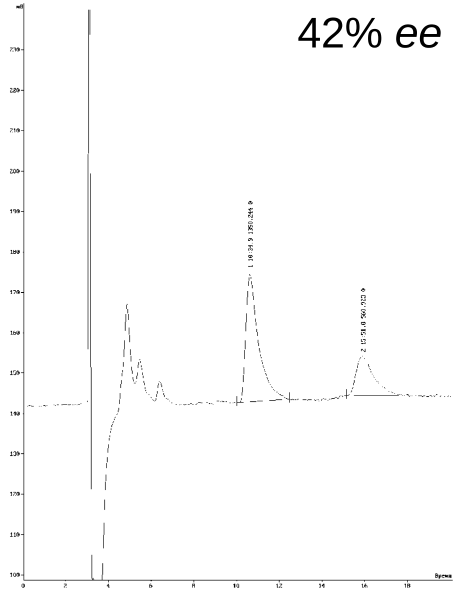

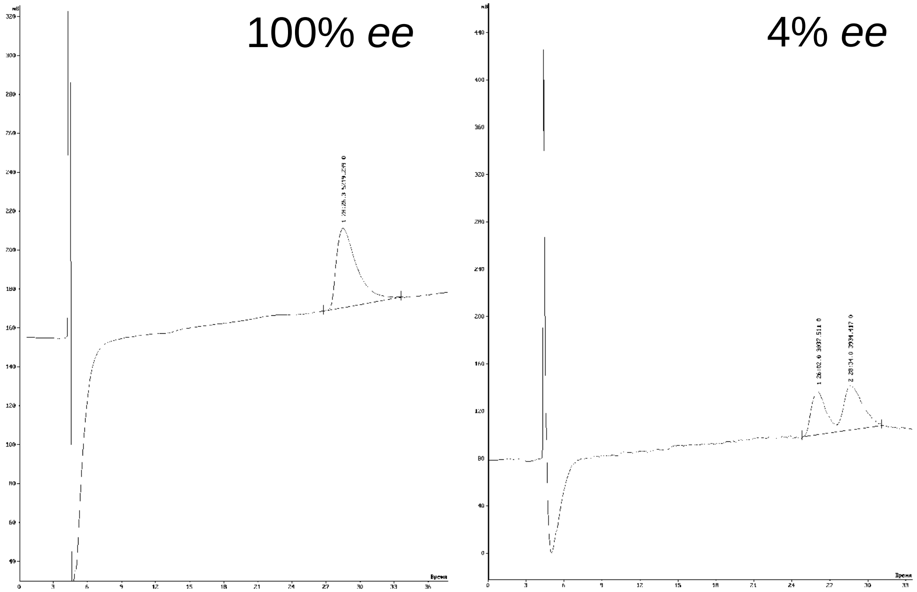

Figure S34. Enantiomeric excess of the isopropyl-substituted amine derived from enantiomerically pure amine chloride **(*R*)-16** upon treatment with Et_3N in benzene determined by HPLC (an Agilent 1100 chromatograph, Chiralcel OD 250 mm × 4.6 mm column, flow rate 1.0 mL/min, UV 254 nm, eluent: hexane/isopropanol/triethylamine = 98/2/0.2)

Figure S35. Enantiomeric excesses of amide **(*R*)-17** obtained from enantiomerically pure amine hydrochloride **(*R*)-16** (left) and amide **17** obtained from racemic amine hydrochloride **16** determined by HPLC (an Agilent 1100 chromatograph, Chiralcel OD 250 mm × 4.6 mm column, flow rate 0.7 mL/min, UV 254 nm, eluent: hexane/isopropanol/triethylamine = 99/1/0.5)

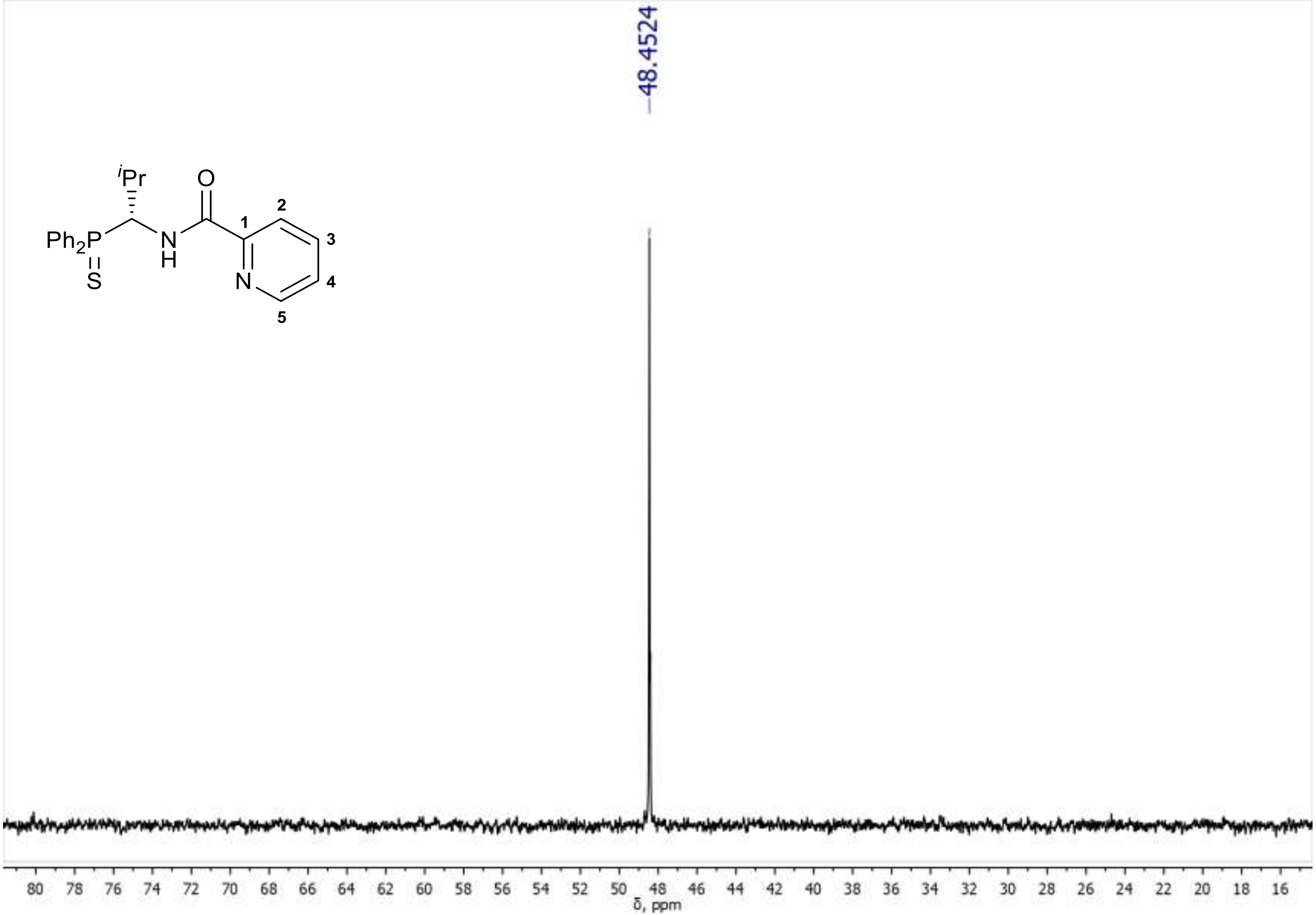

Figure S36. $^{31}P\{^{1}H\}$ NMR spectrum of ligand **17** (161.98 MHz, $CDCl_3$)

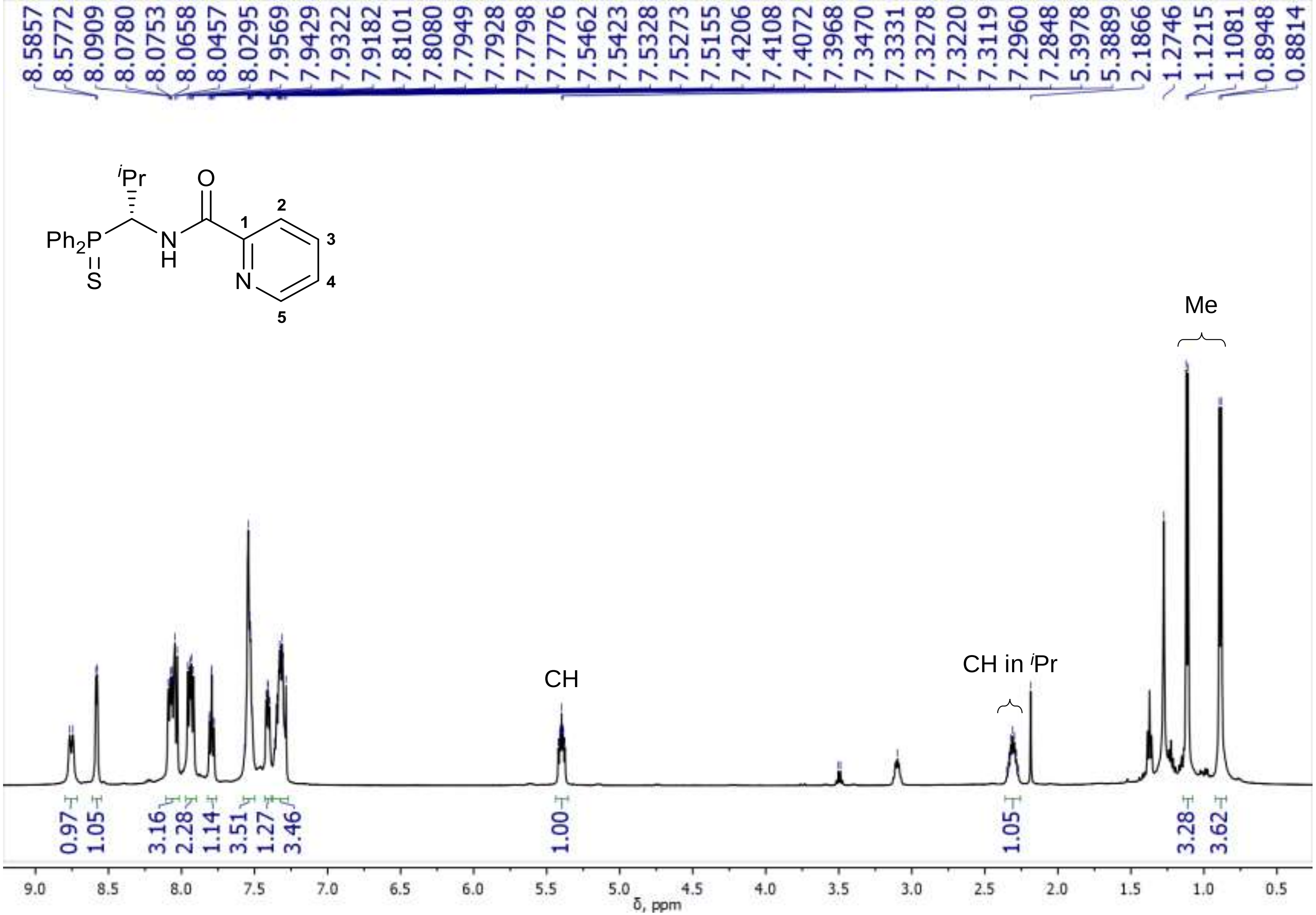

Figure S37. ^{1}H NMR spectrum of ligand **17** (500.13 MHz, $CDCl_3$)

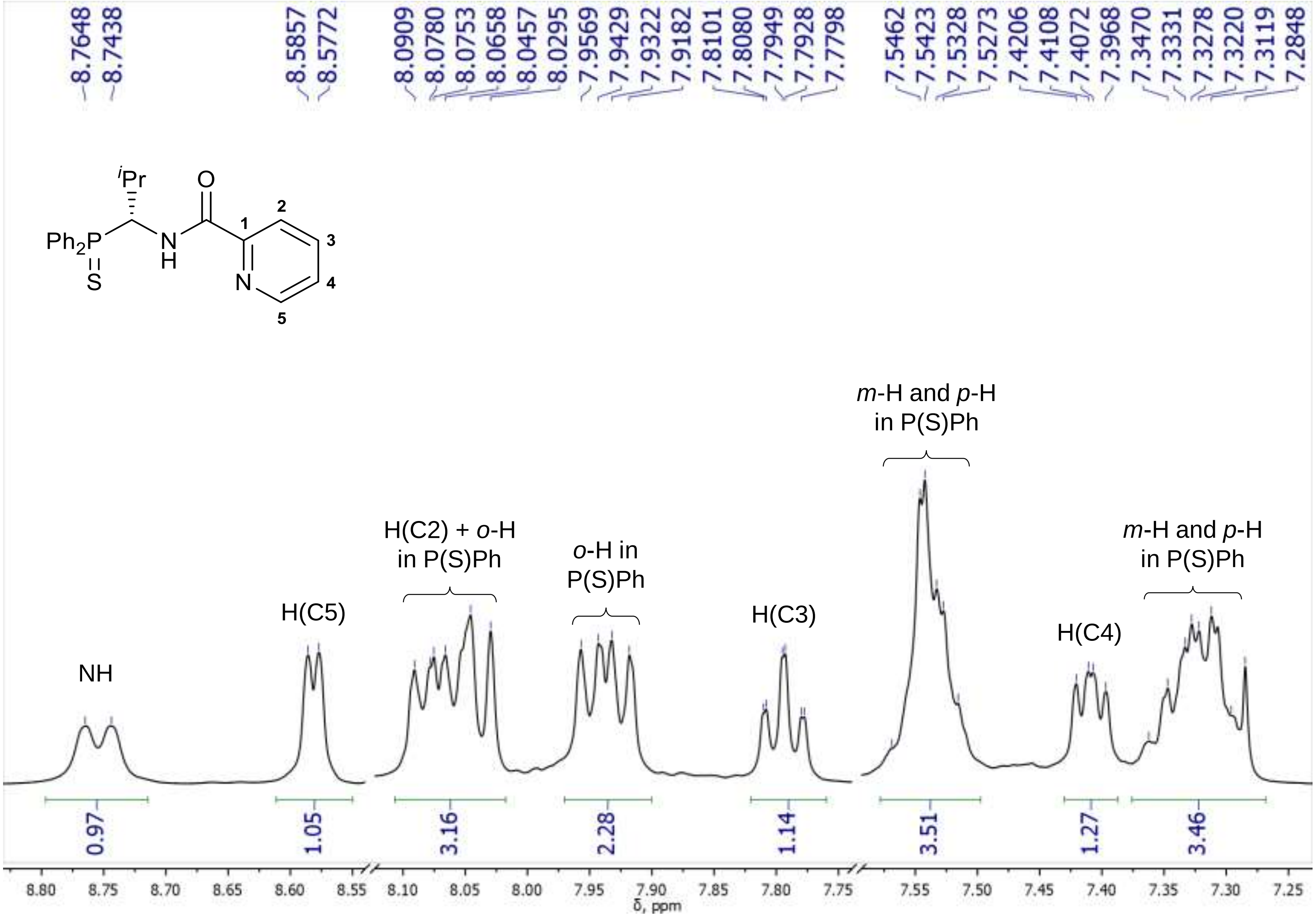

Figure S38. Extended fragments of the ^{1}H NMR spectrum of ligand **17** (500.13 MHz, $CDCl_3$)

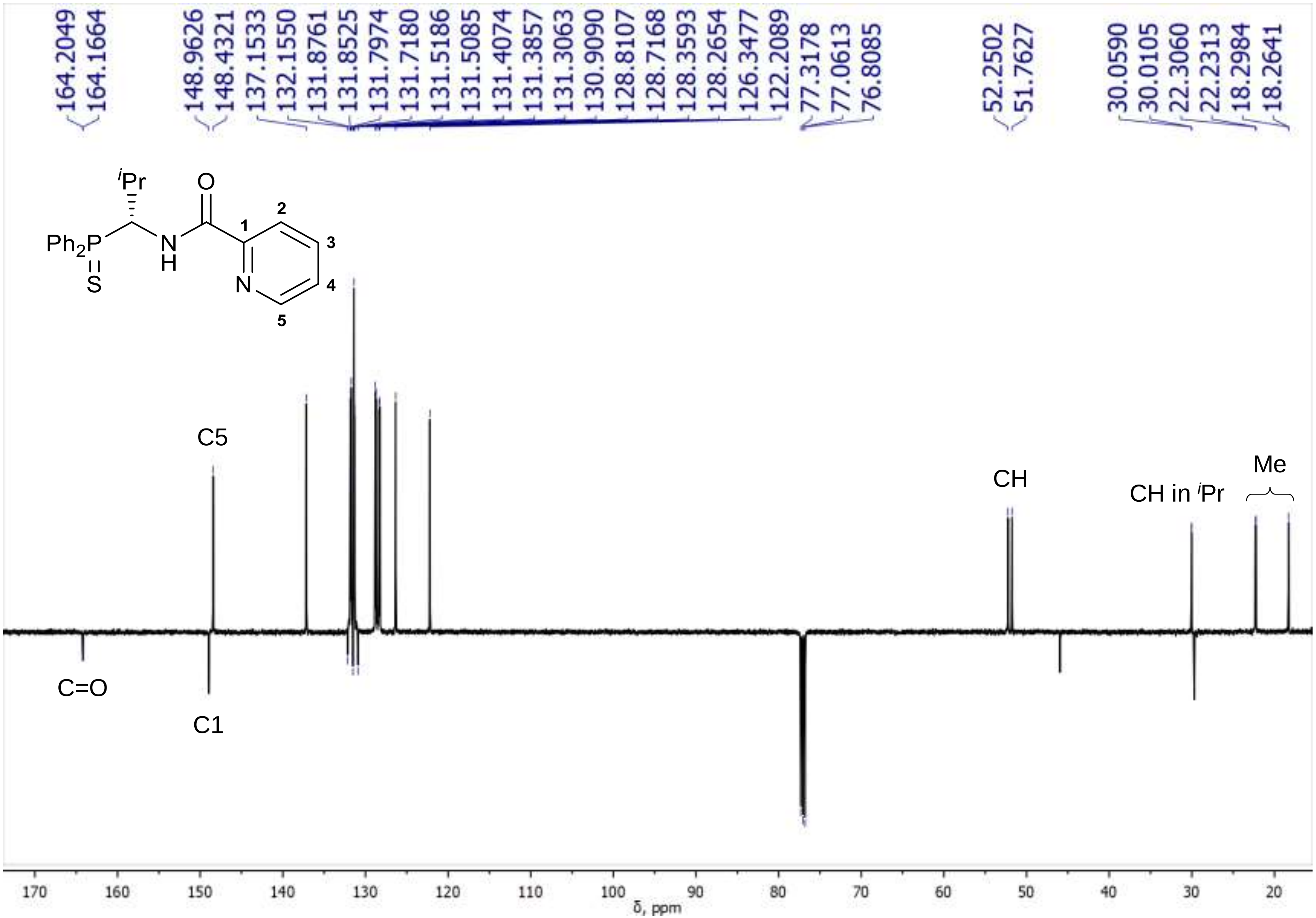

Figure S39. $^{13}C\{^{1}H\}$ spectrum of ligand **17** (125.76 MHz, $CDCl_3$)

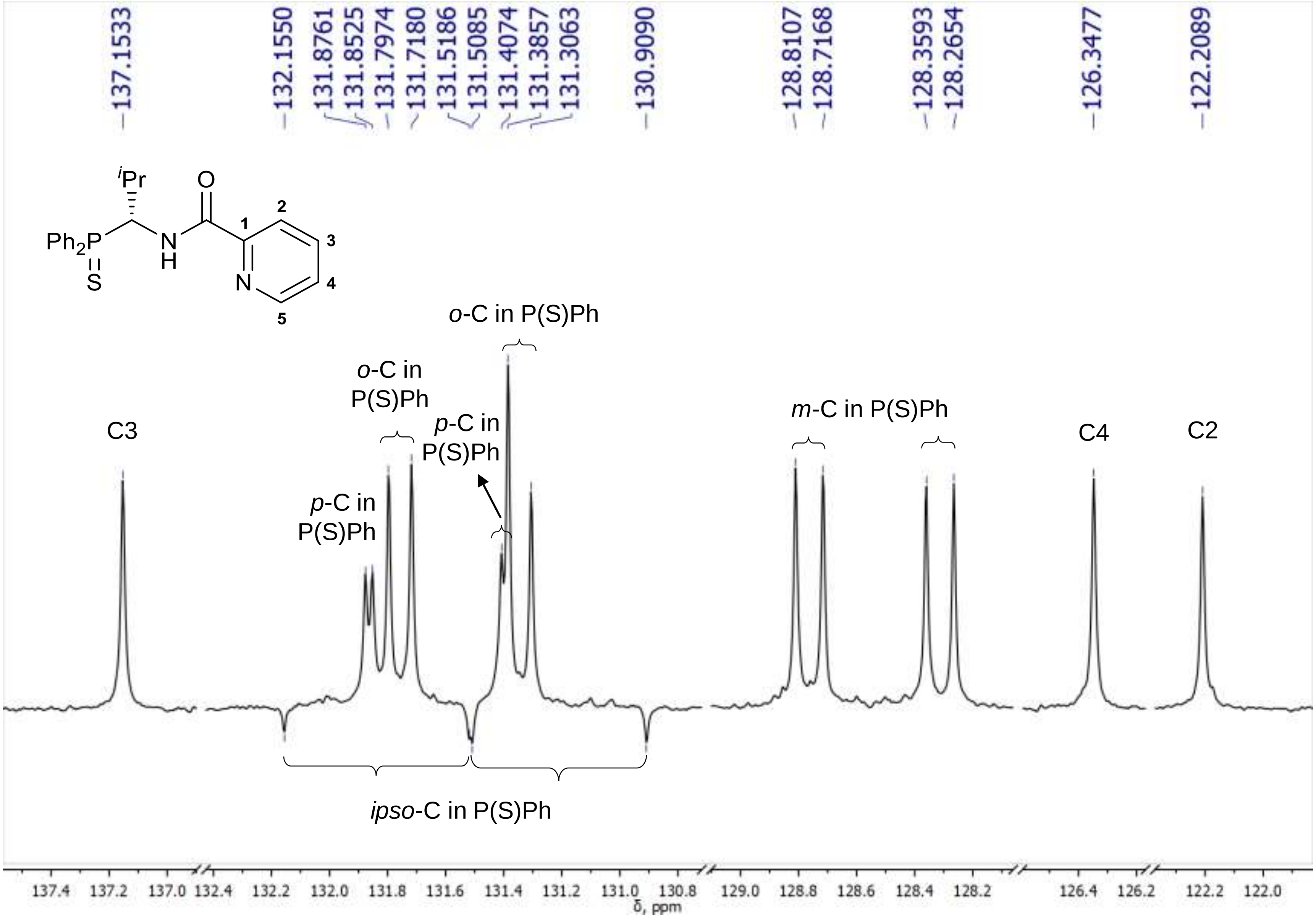

Figure S40. Extended fragments of the $^{13}C\{^1H\}$ spectrum of ligand **17** (125.76 MHz, $CDCl_3$)

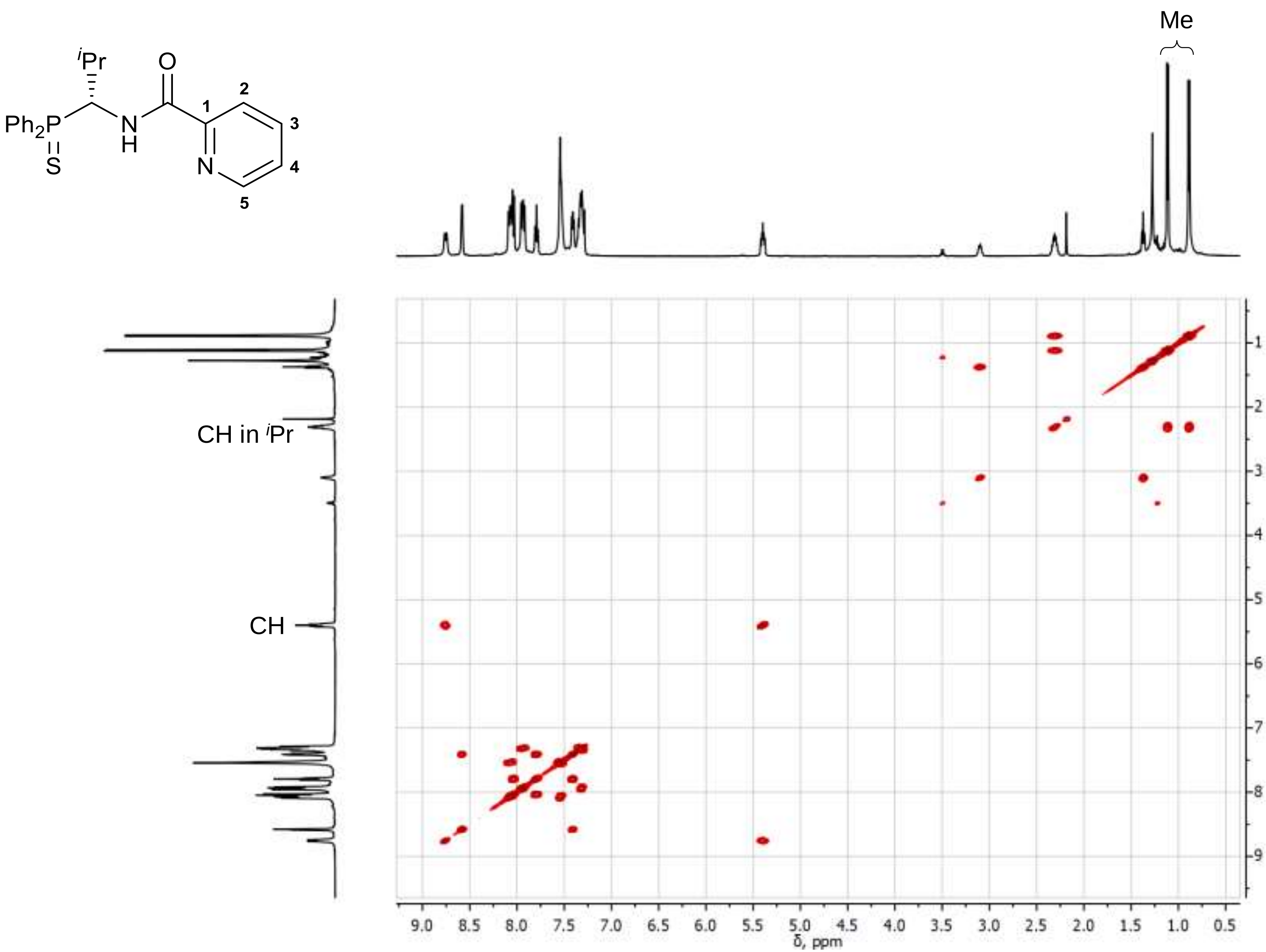

Figure S41. ^{1}H–^{1}H COSY spectrum of ligand **17** (500.13 MHz, $CDCl_3$)

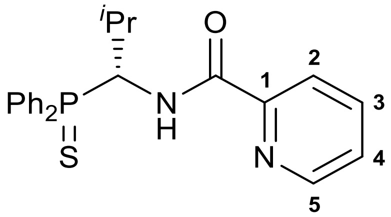

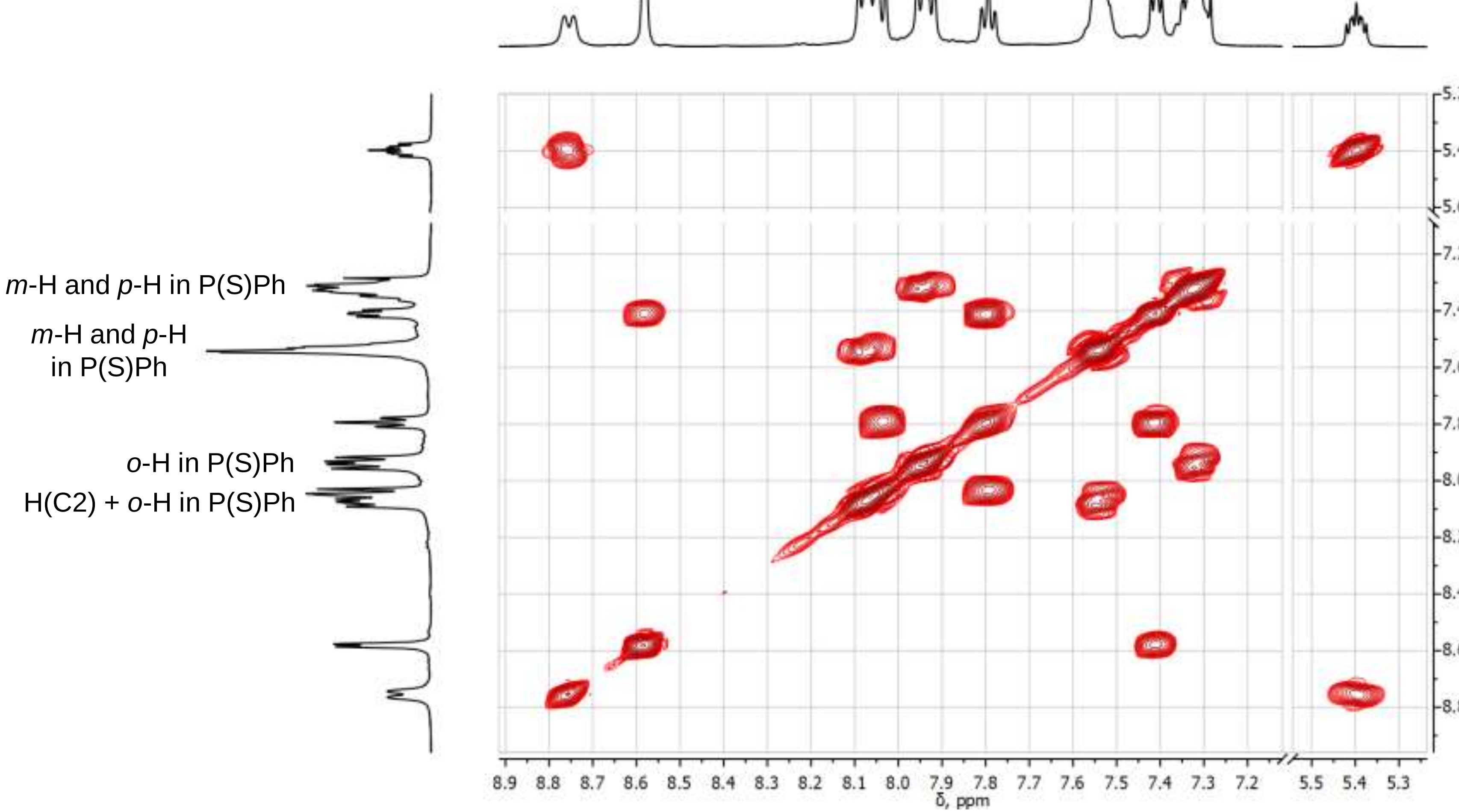

Figure S42. Extended fragments of the $^{1}H-^{1}H$ COSY spectrum of ligand **17** (500.13 MHz, $CDCl_3$)

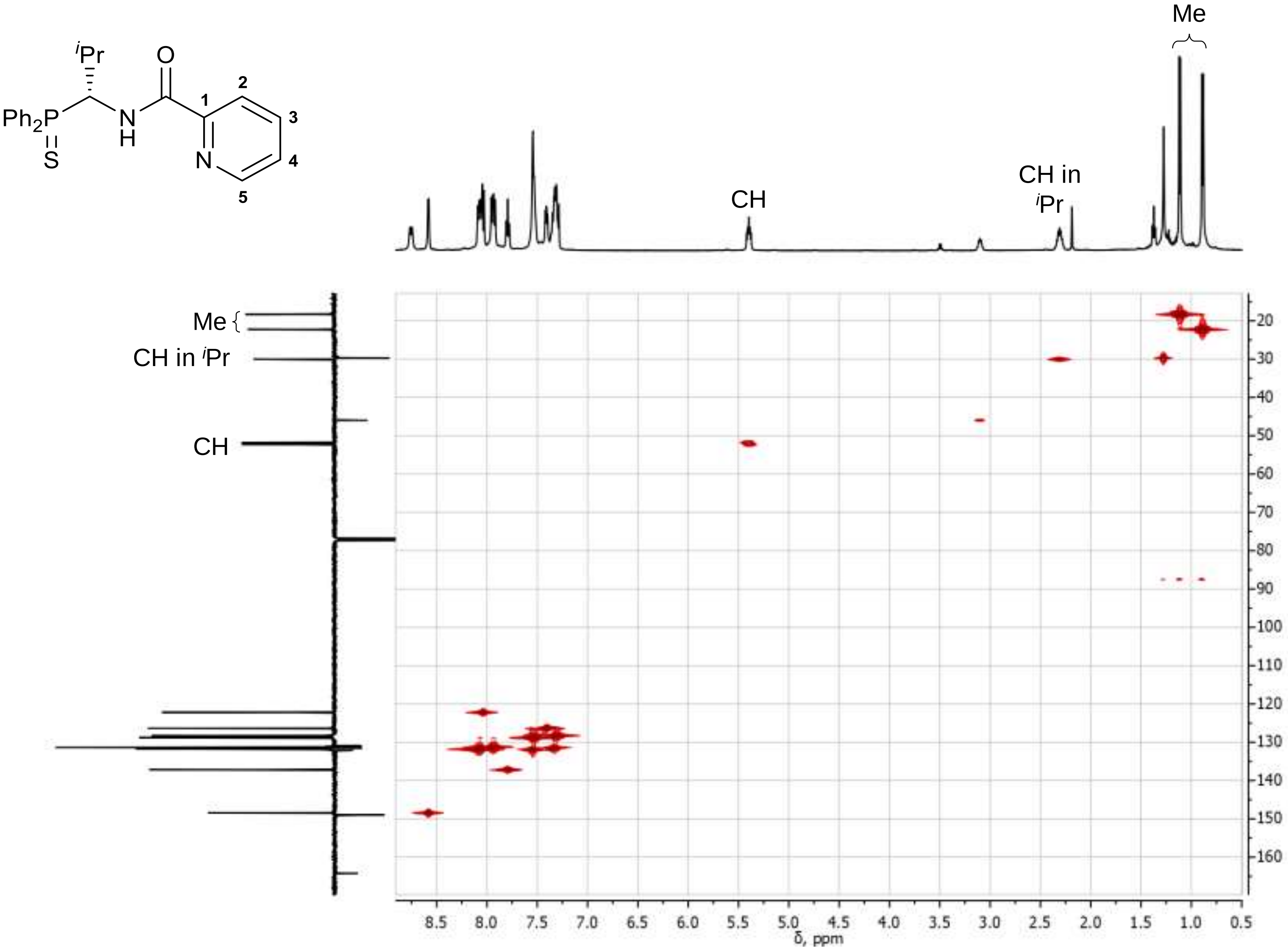

Figure S43. HMQC spectrum of ligand **17** ($CDCl_3$)

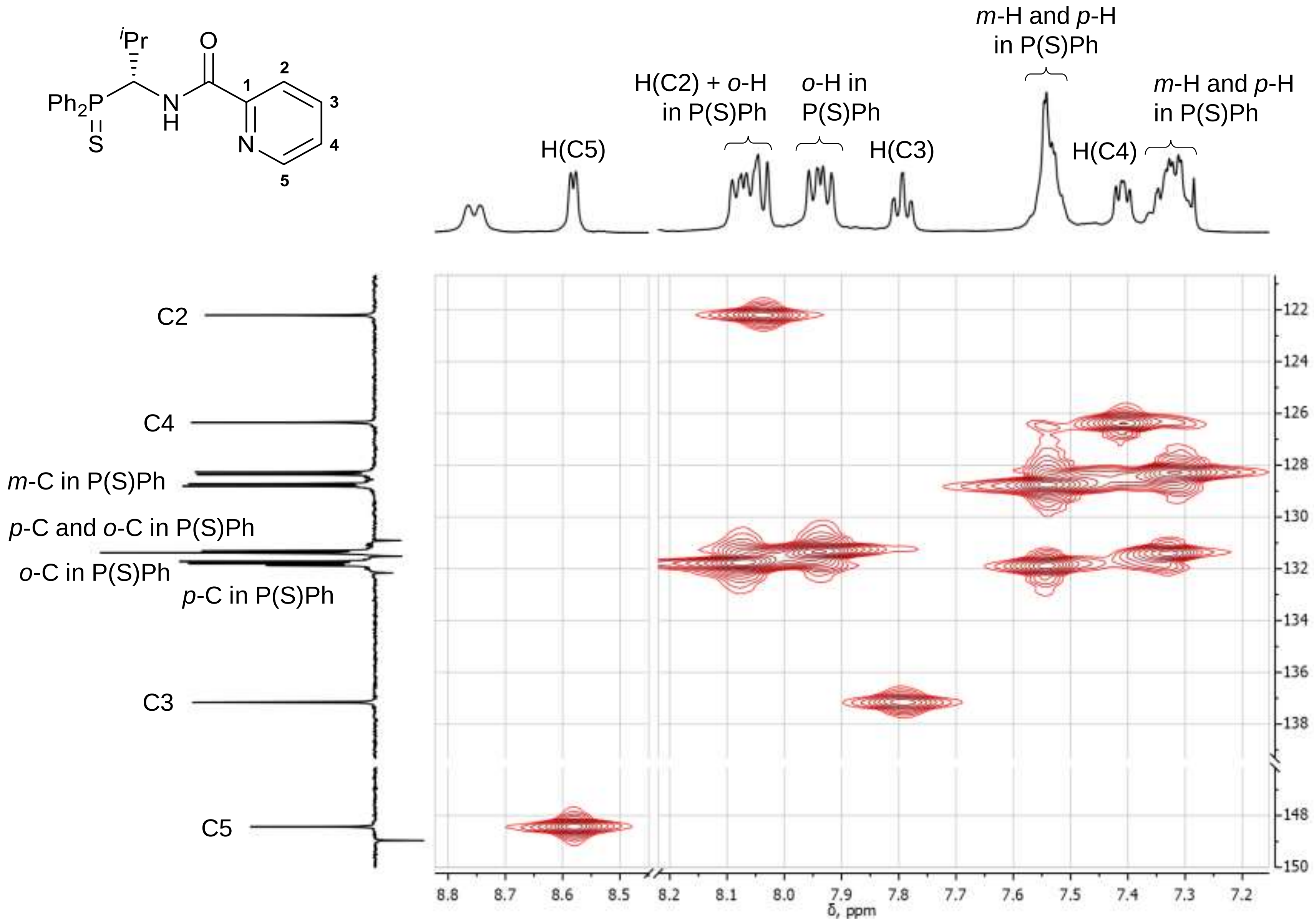

Figure S44. Extended fragments of the HMQC spectrum of ligand **17** ($CDCl_3$)

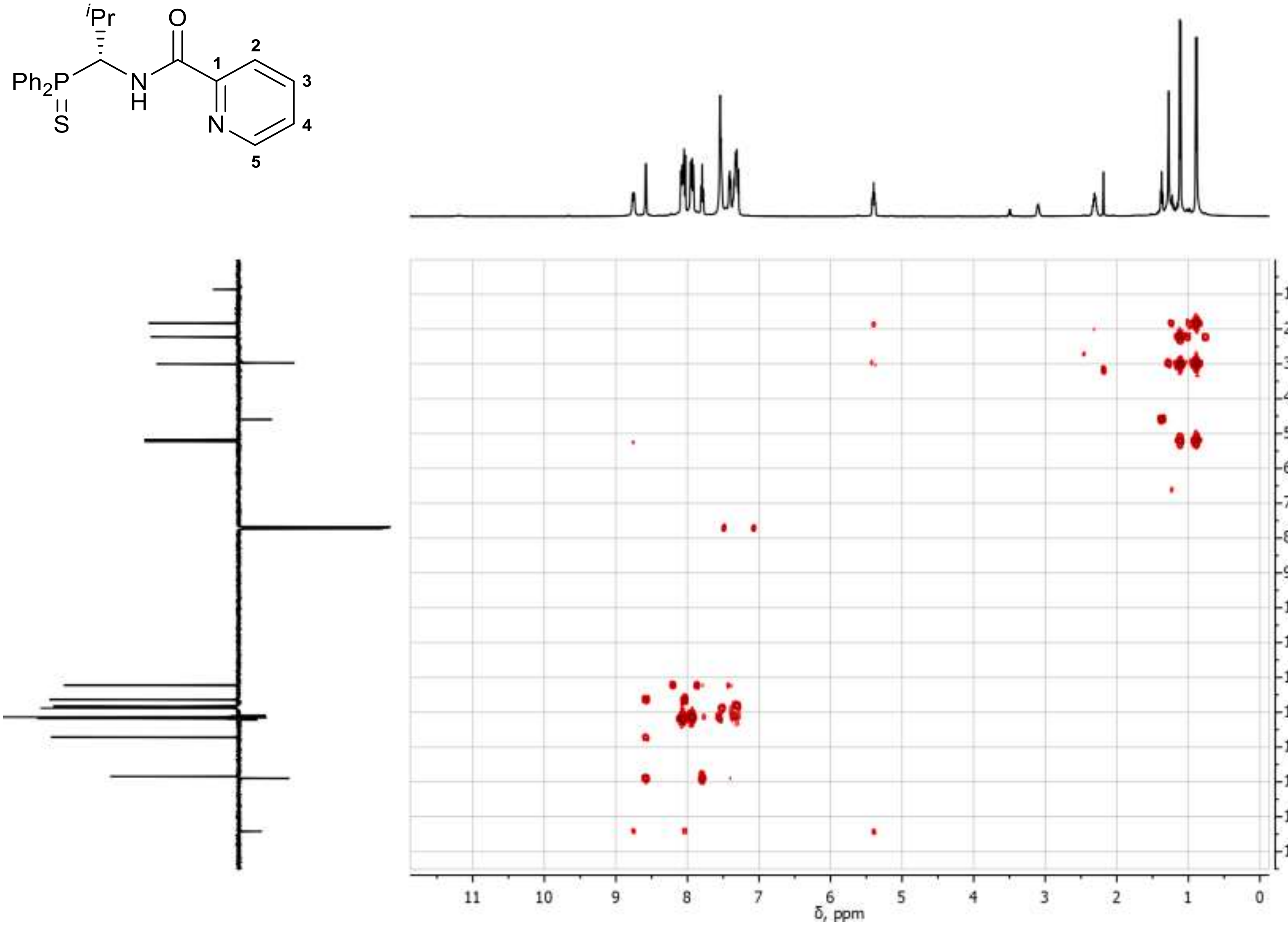

Figure S45. $^{1}H-^{13}C$ HMBC spectrum of ligand **17** ($CDCl_3$)

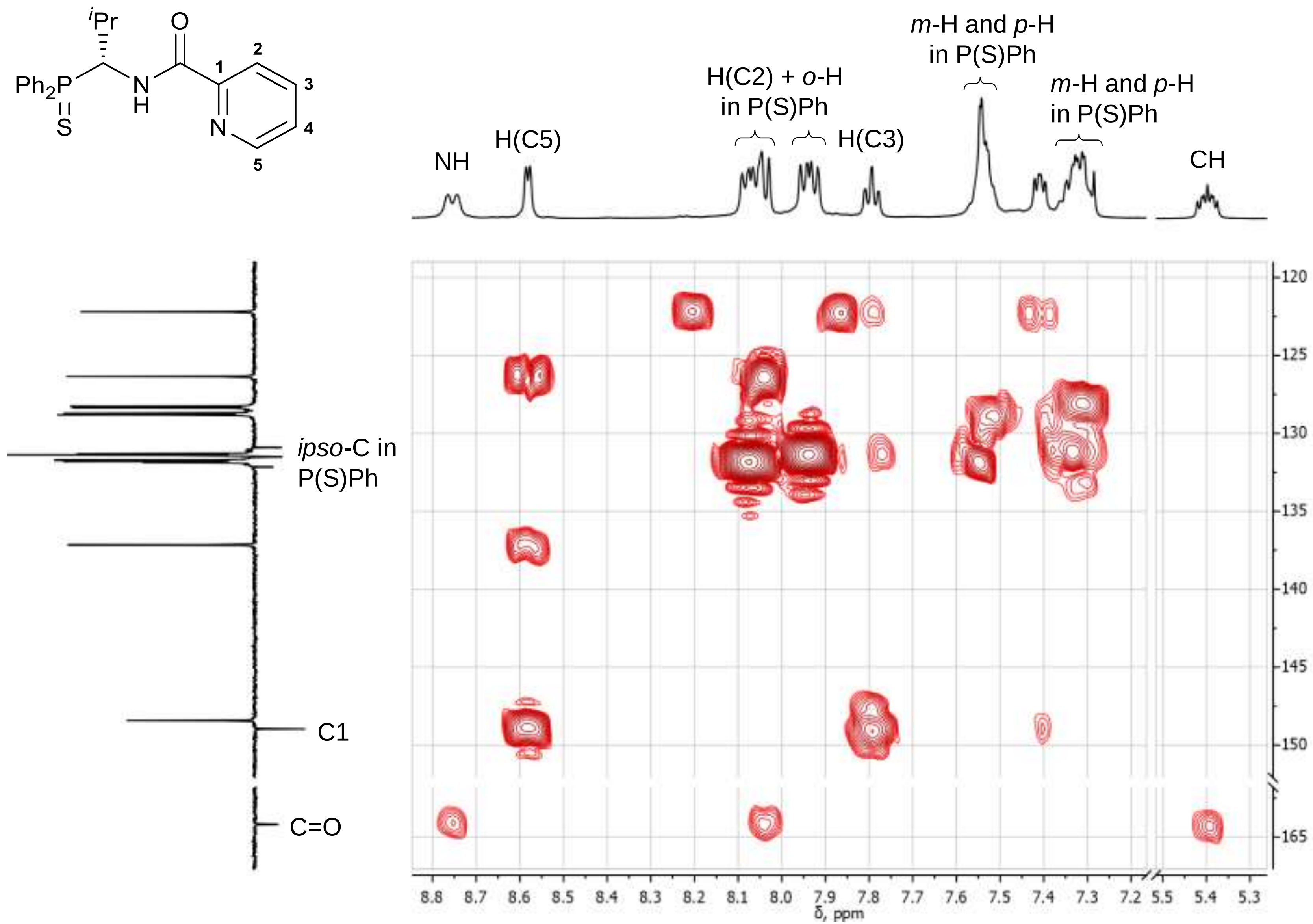

Figure S46. Extended fragments of the $^{1}H-^{13}C$ HMBC spectrum of ligand **17** ($CDCl_3$)

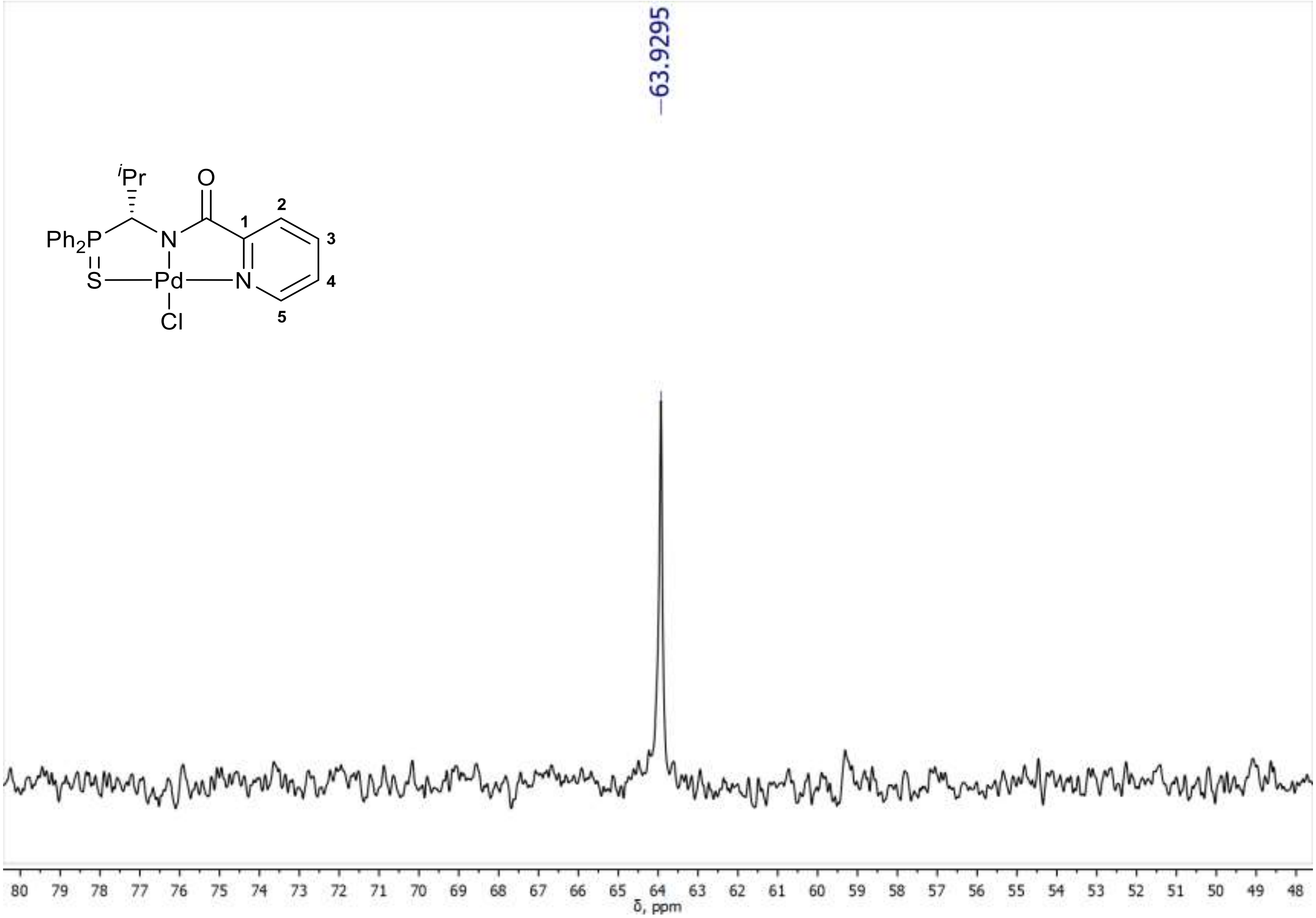

Figure S47. $^{31}P\{^{1}H\}$ NMR spectrum of complex **18** (121.49 MHz, $CDCl_3$)

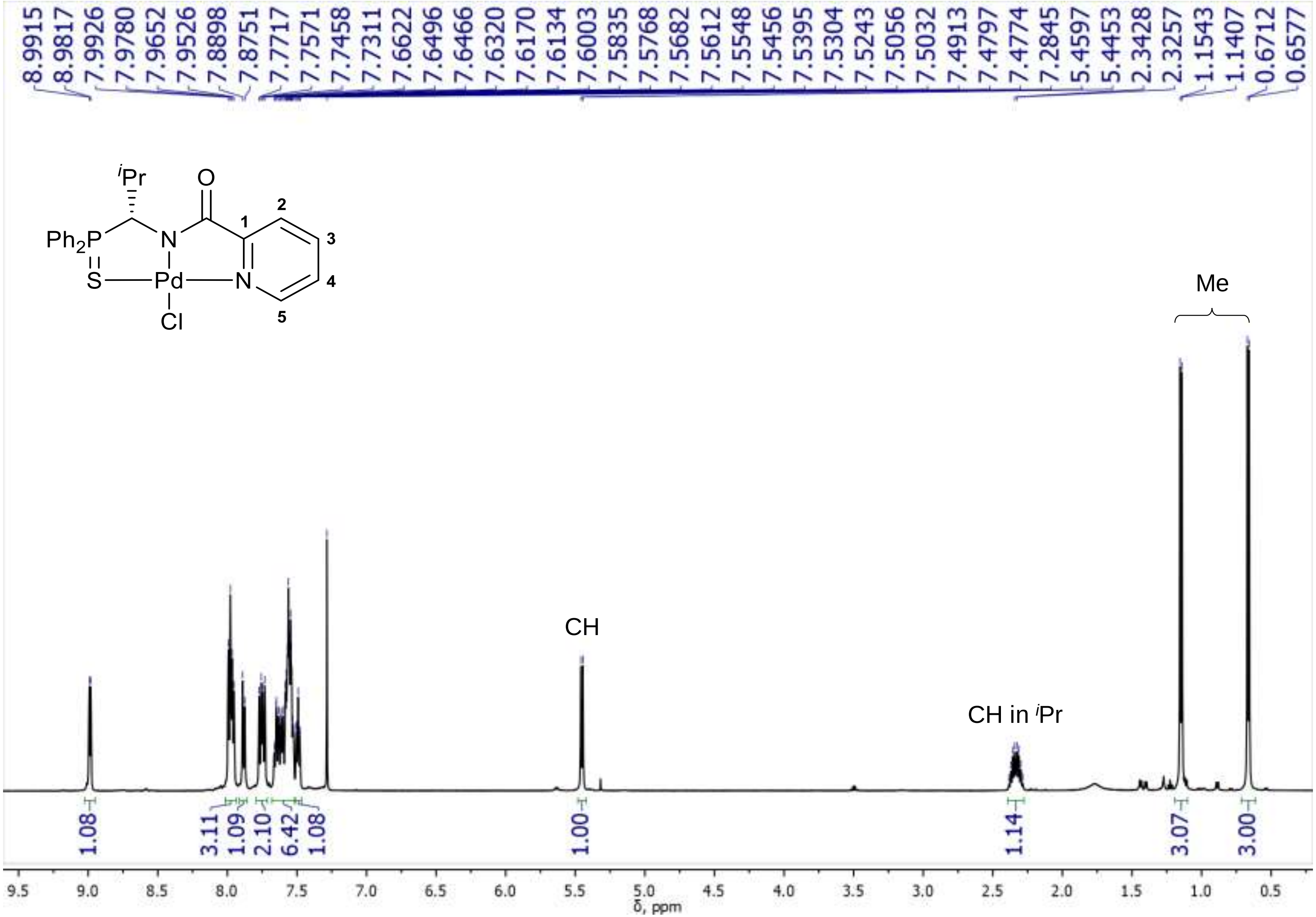

Figure S48. ^{1}H NMR spectrum of complex **18** (500.13 MHz, $CDCl_3$)

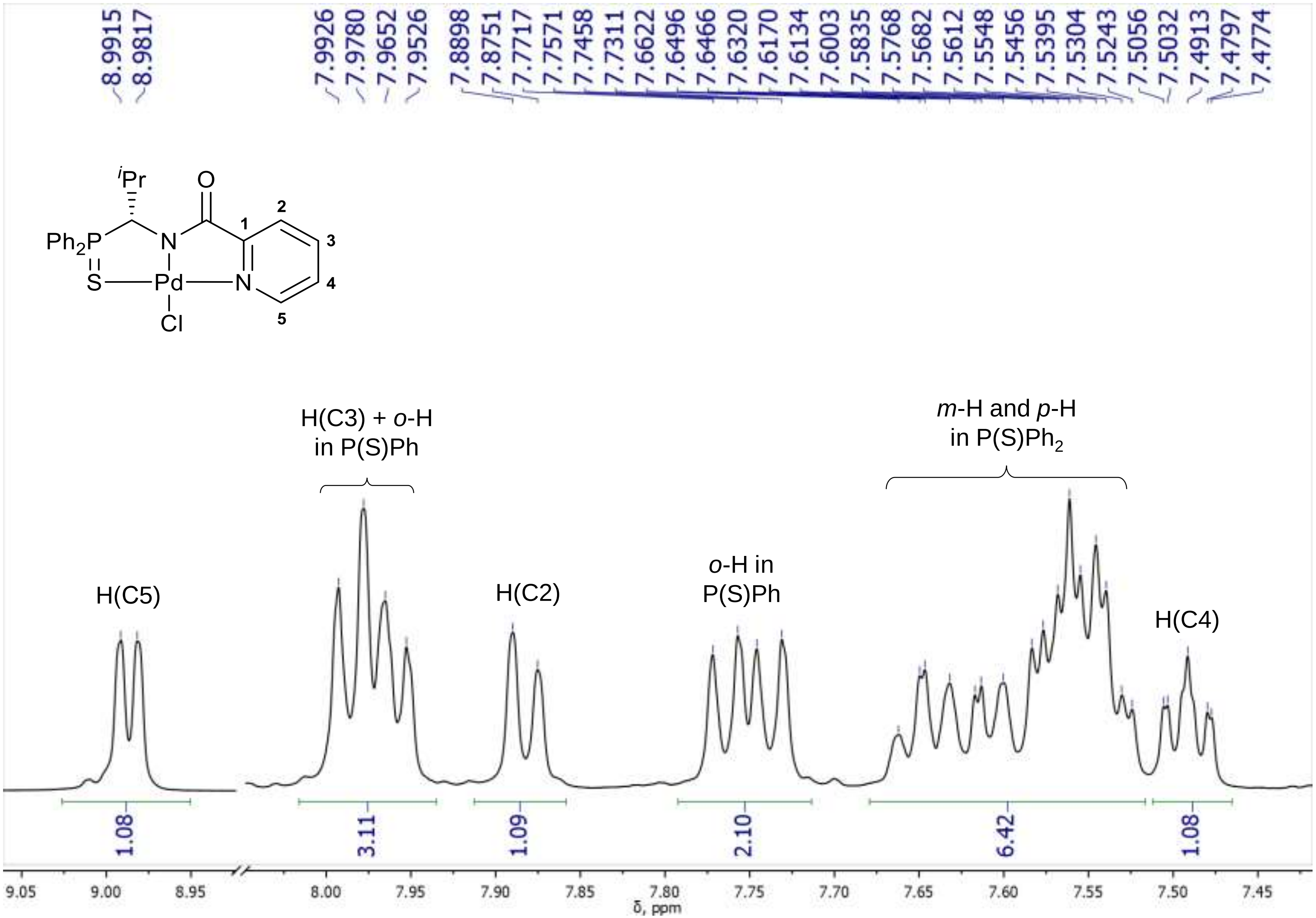

Figure S49. Extended fragments of the ^{1}H NMR spectrum of complex **18** (500.13 MHz, $CDCl_3$)

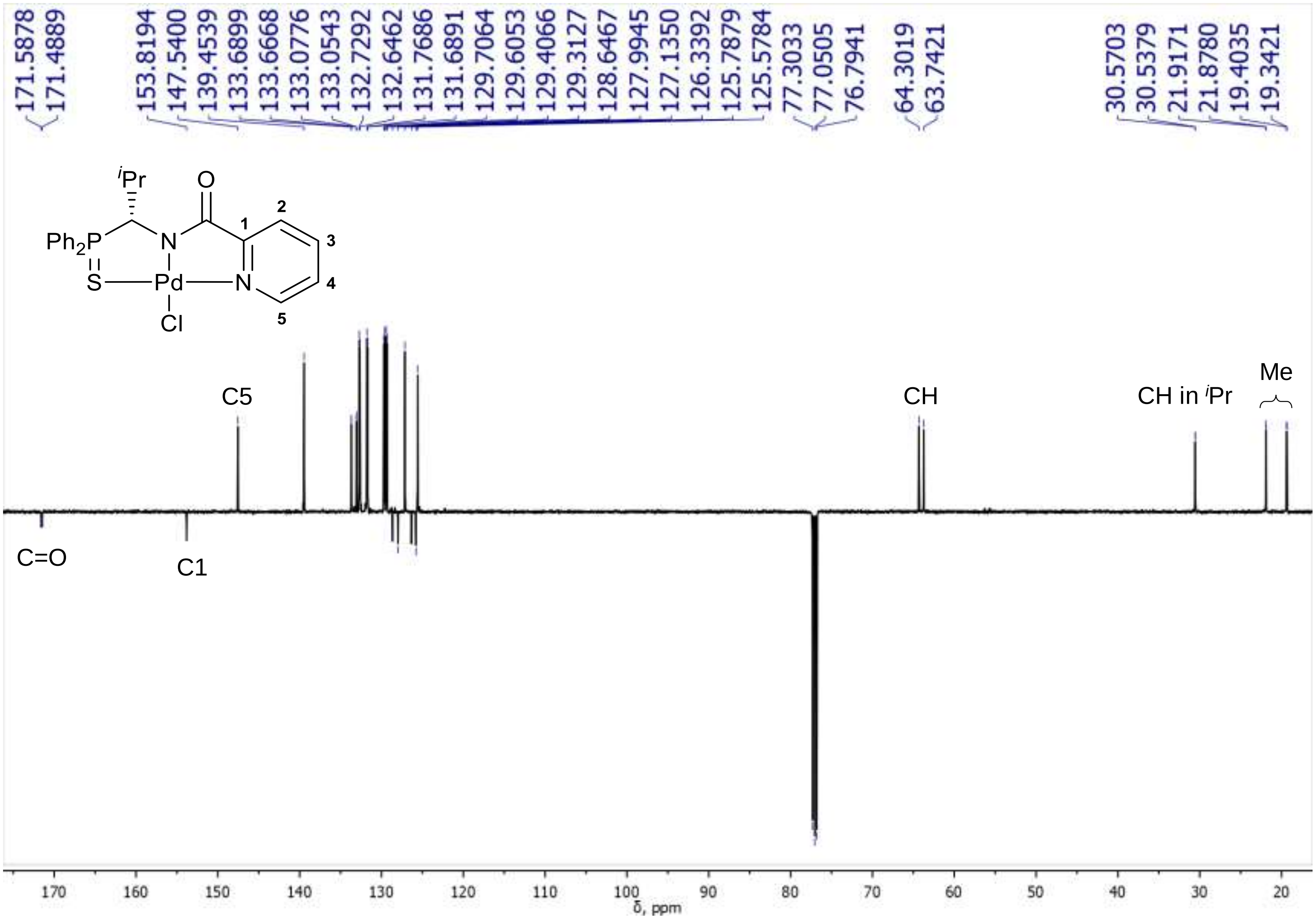

Figure S50. $^{13}C\{^1H\}$ spectrum of complex **18** (125.76 MHz, $CDCl_3$)

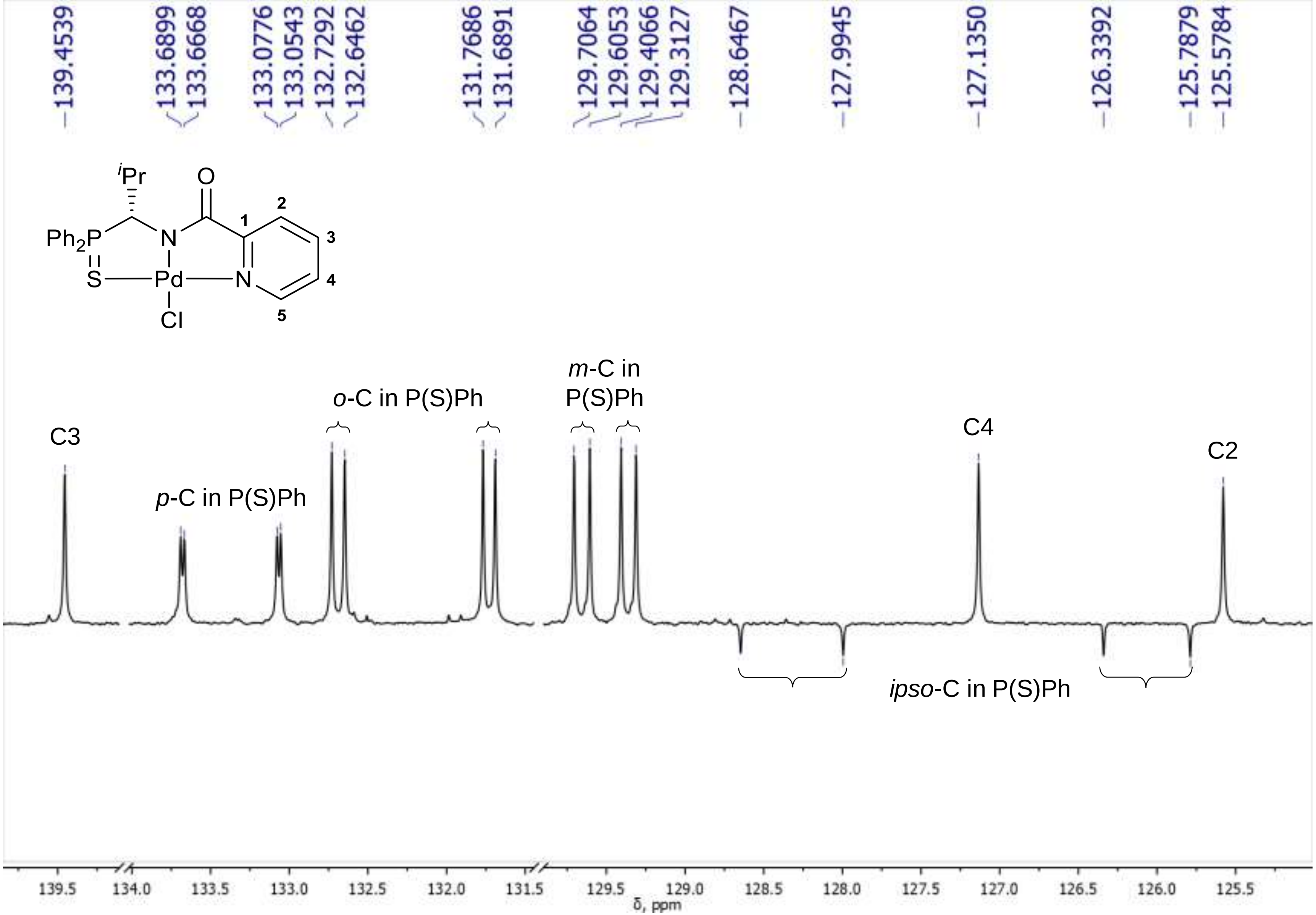

Figure S51. Extended fragments of the $^{13}C\{^1H\}$ spectrum of complex **18** (125.76 MHz, $CDCl_3$)

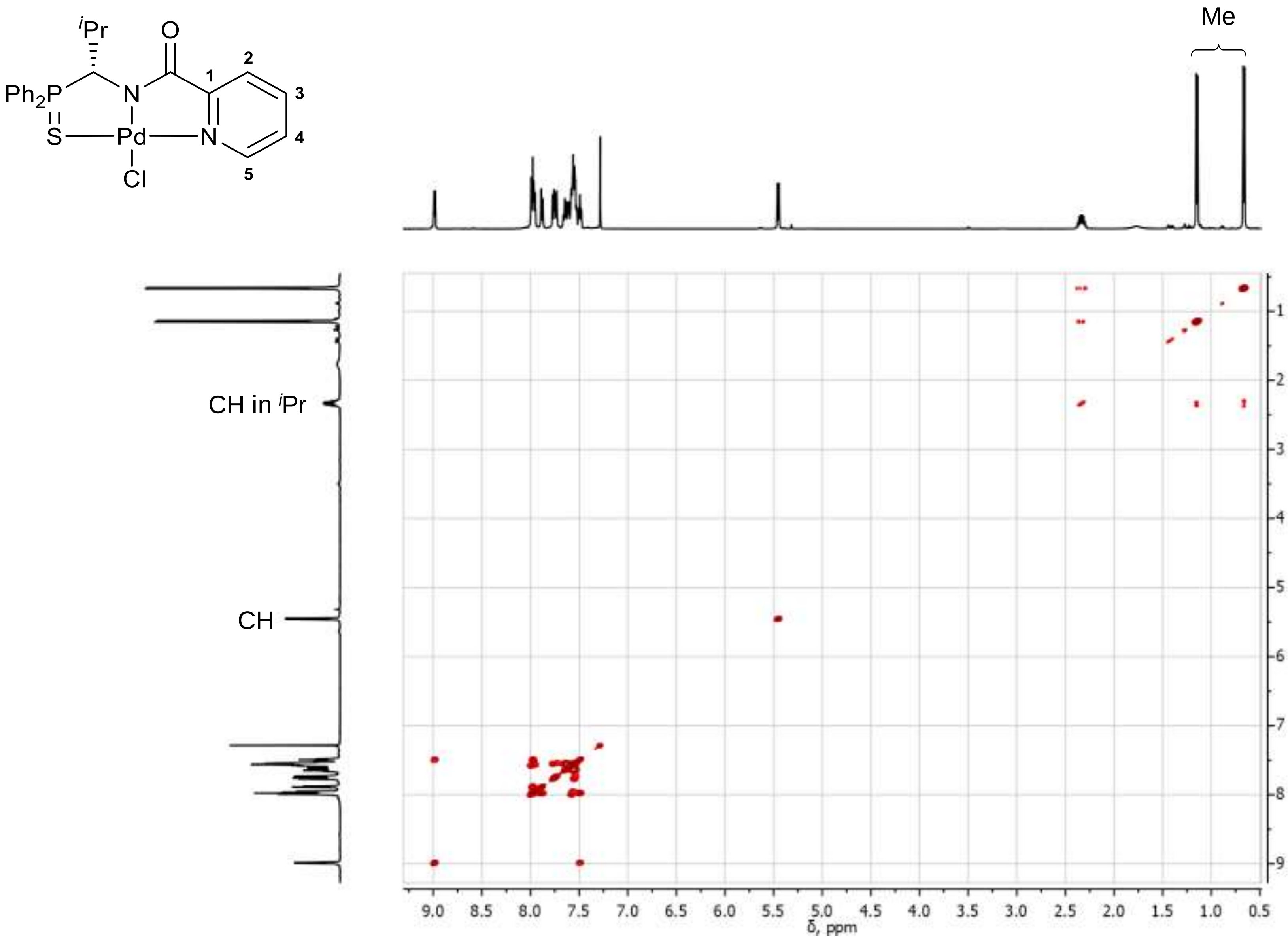

Figure S52. $^{1}H-^{1}H$ COSY spectrum of complex **18** (500.13 MHz, $CDCl_3$)

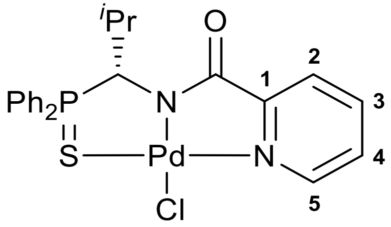

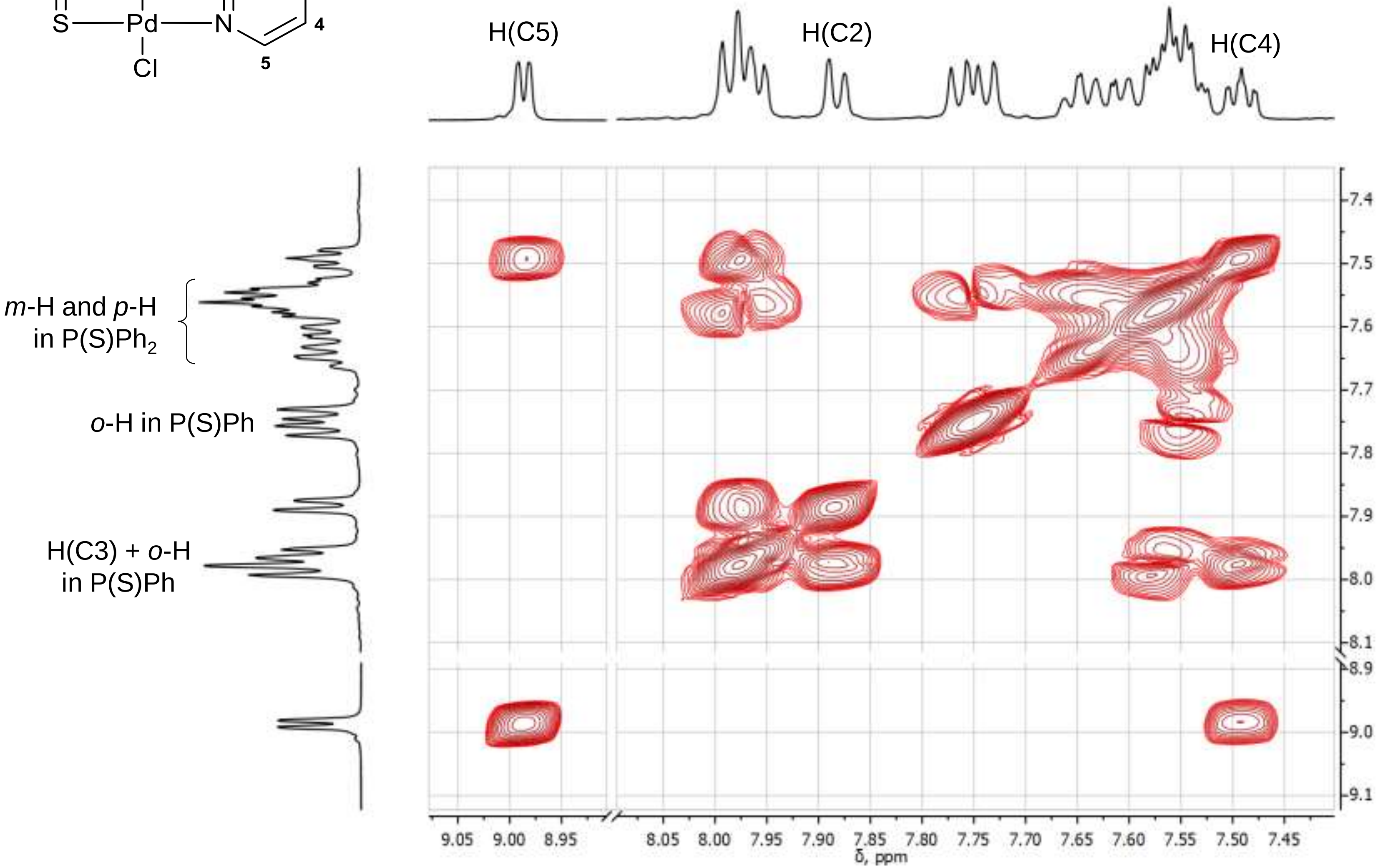

Figure S53. Extended fragments of the $^{1}H{-}^{1}H$ COSY spectrum of complex **18** (500.13 MHz, $CDCl_3$)

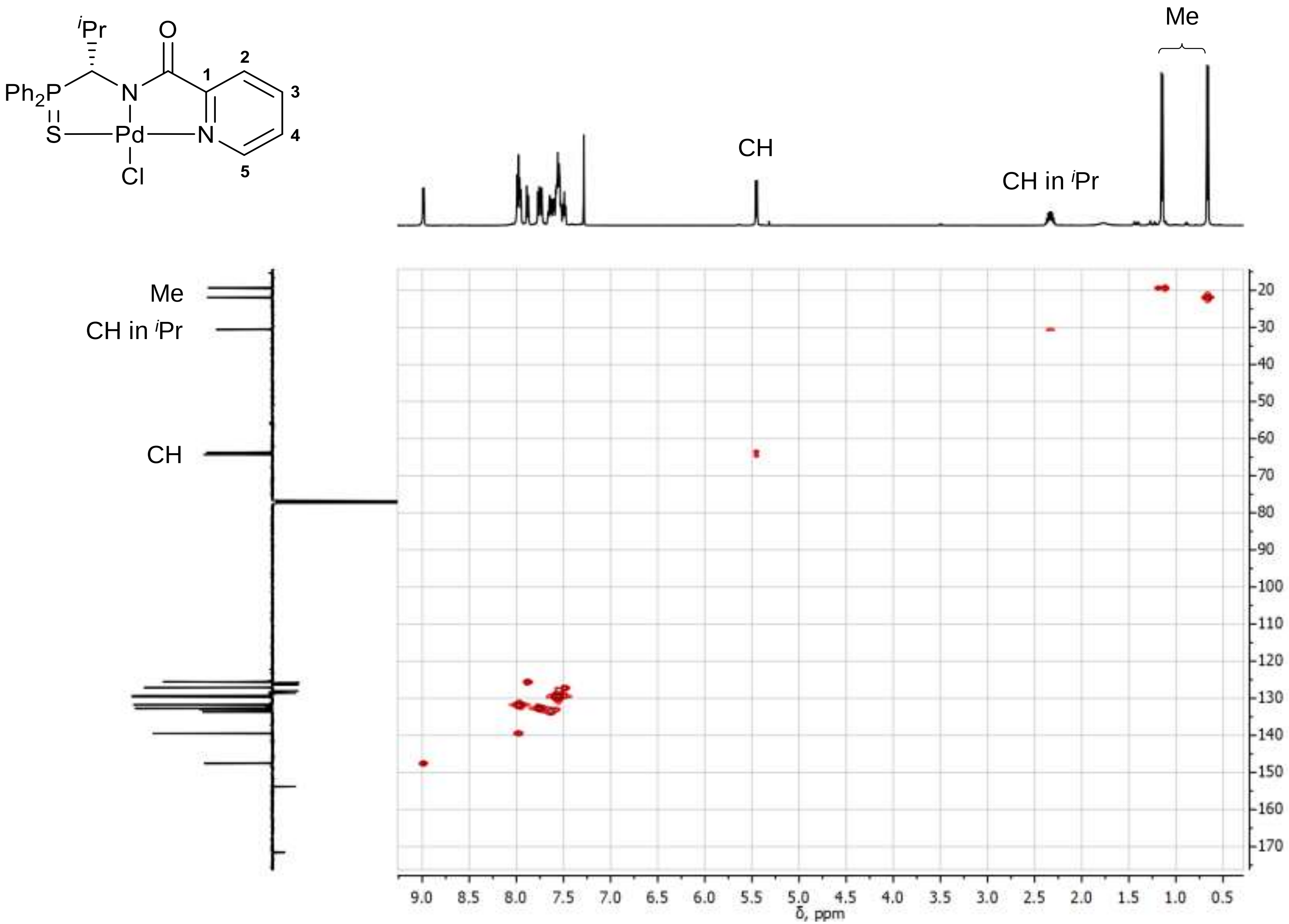

Figure S54. HMQC spectrum of complex **18** ($CDCl_3$)

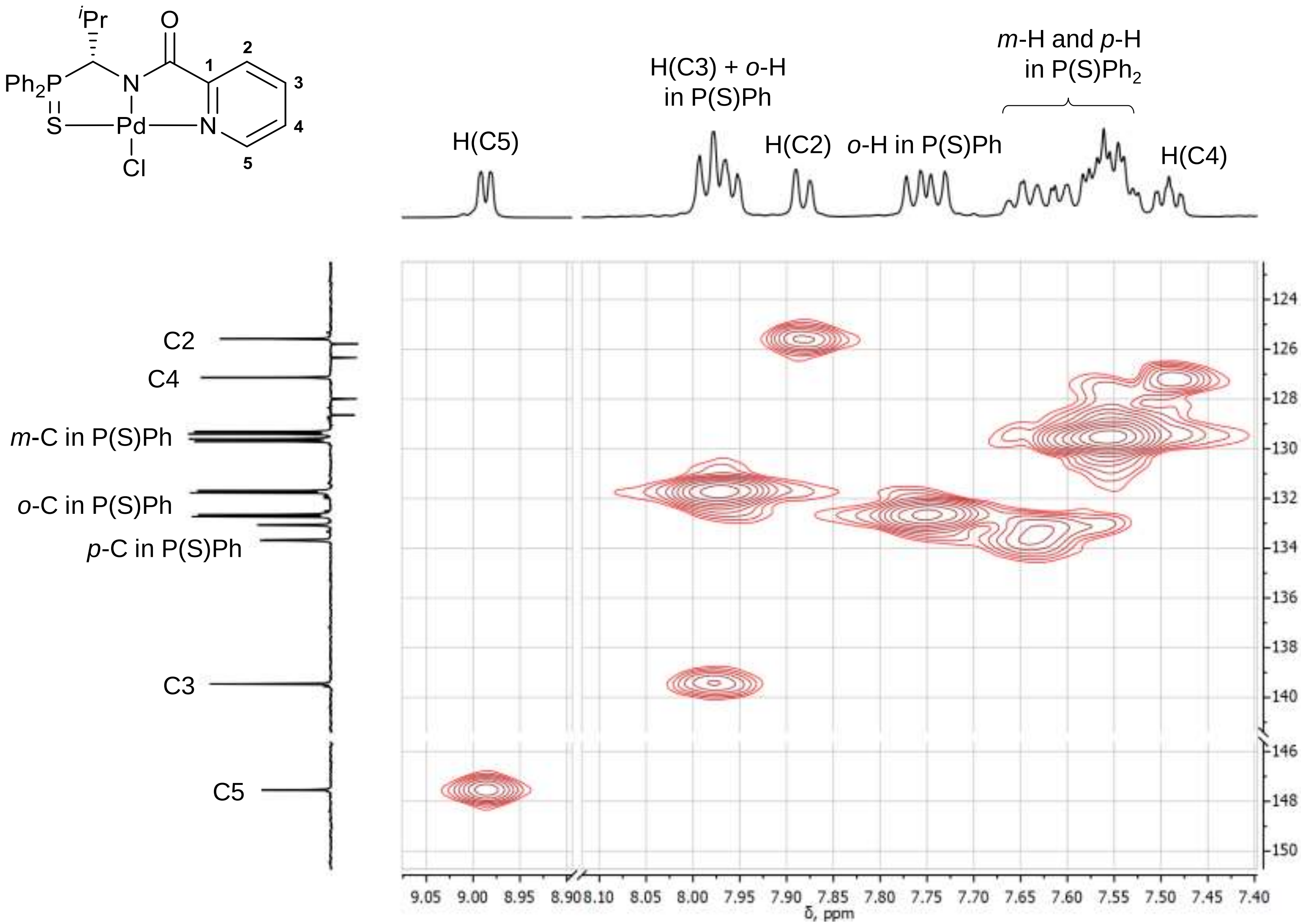

Figure S55. Extended fragments of the HMQC spectrum of complex **18** ($CDCl_3$)

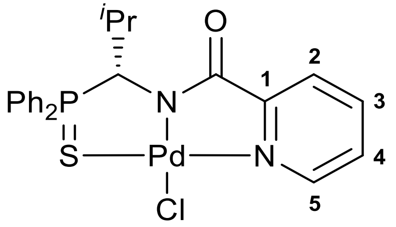

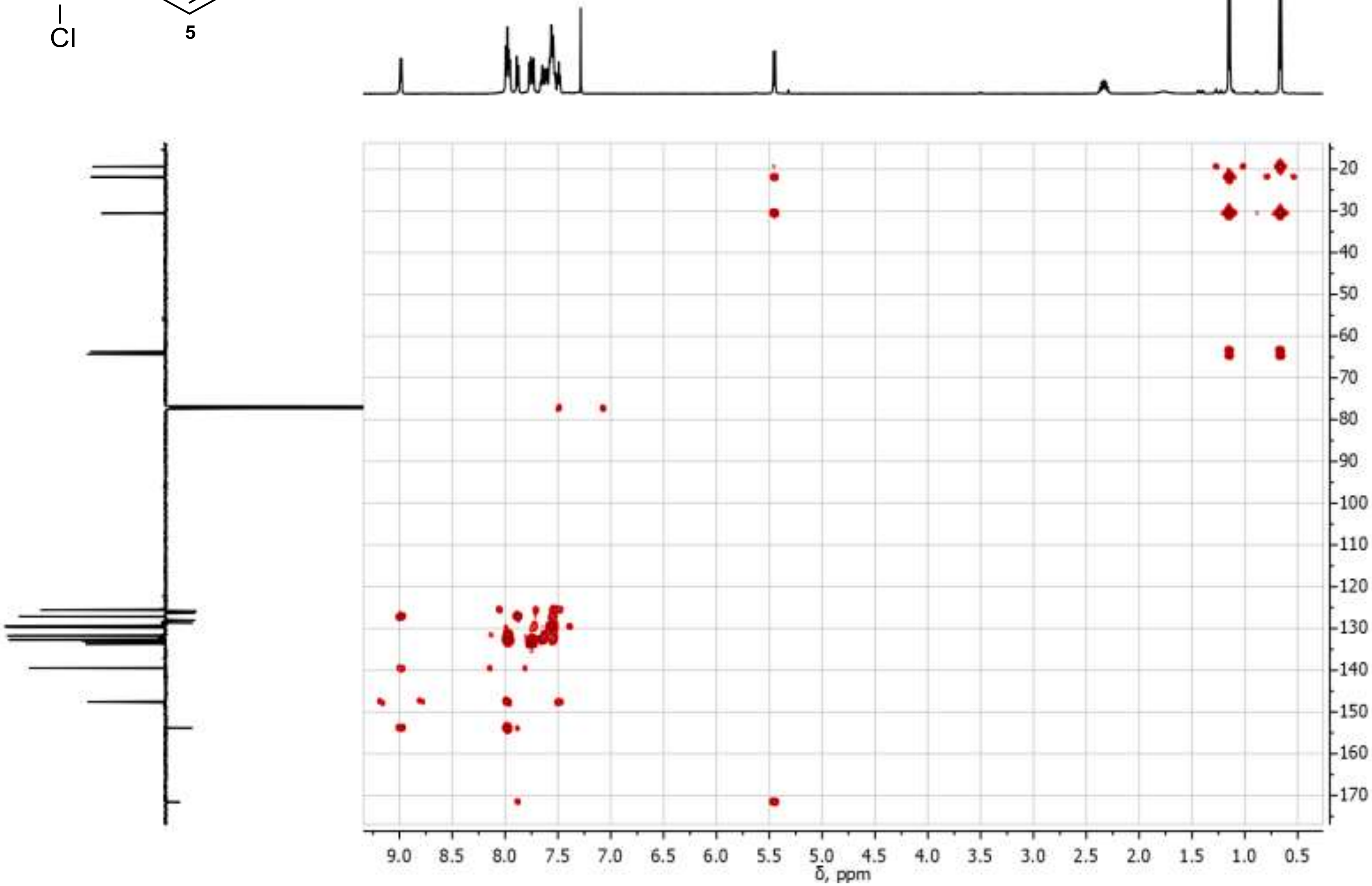

Figure S56. $^{1}H-^{13}C$ HMBC spectrum of complex **18** ($CDCl_3$)

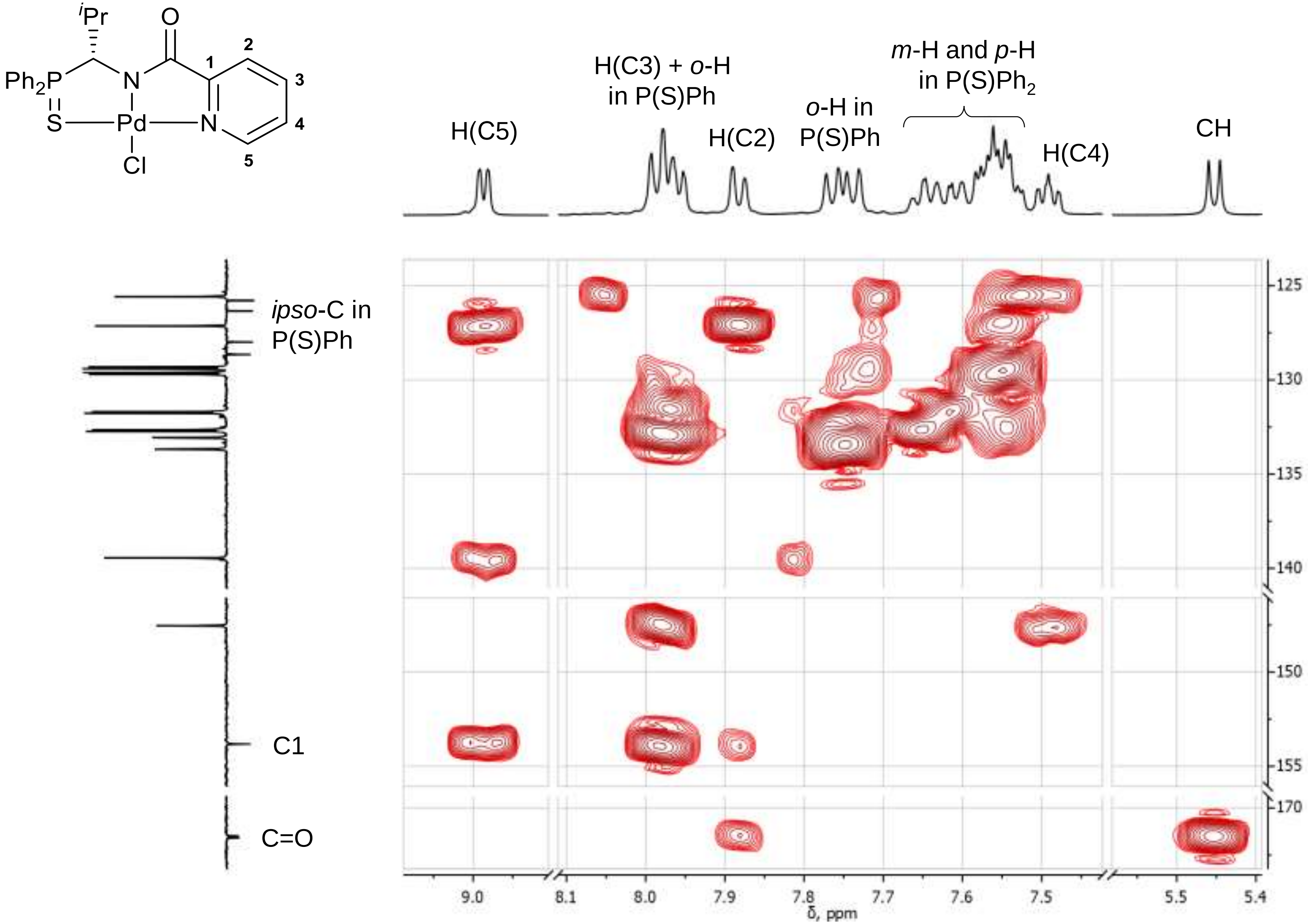

Figure S57. Extended fragments of the $^{1}H-^{13}C$ HMBC spectrum of complex **18** ($CDCl_3$)

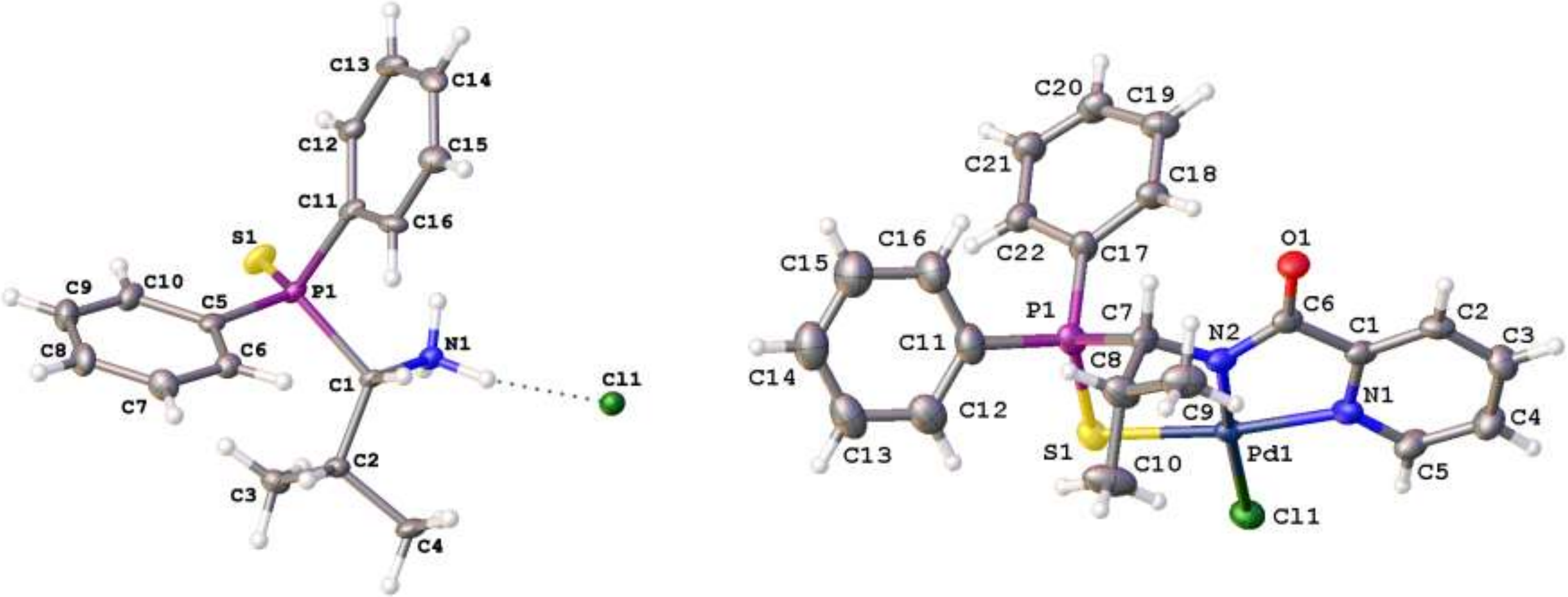

Figure S58. General views of racemic amine hydrochloride **16** (left) and palladocycle **18** (right)
The minor components of the disorder and the second symmetry-independent molecule of the complex are not shown.

Table S2. Crystal data and structure refinement parameters for compounds **8**, **9**, **10a**, **10b**, **11**, **12**, **13a**, **13b**, **16**, **18**, **(*R*)-17**, and **(*R*)-18**

	**8**	**9**	**10a**	**10b**	**11**	**12**
Empirical formula	$C_{27}H_{24}NOPS_2$	$C_{25}H_{19}Cl_2N_2OPPdS$	$C_{29}H_{22}ClN_2OPPdS$	$C_{24}H_{20}Cl_3N_2OPPdS$	$C_{23}H_{18}ClN_2OPPdS$	$C_{27}H_{20}ClN_2OPPdS_2$
Formula weight	473.56	603.75	619.36	628.20	543.27	625.39
T, K	120	120	120	120	120	100
Crystal system	Orthorhombic	Triclinic	Triclinic	Triclinic	Triclinic	Monoclinic
Space group	Pbca	$P\bar{1}$	$P\bar{1}$	$P\bar{1}$	$P\bar{1}$	$P2_1/c$
Z	8	2	2	2	2	4
a, Å	12.6191(3)	9.4875(2)	10.0728(11)	10.1166(6)	9.5387(4)	11.5015(2)
b, Å	18.4530(5)	11.9332(2)	11.0416(12)	10.8560(7)	10.5630(5)	13.5264(3)
c, Å	21.0409(5)	12.0789(2)	13.2235(15)	12.4535(8)	12.1222(5)	16.8598(4)
α, °	90	106.0390(10)	102.056(2)	85.1660(10)	108.4920(10)	90
β, °	90	106.9170(10)	104.424(2)	70.9540(10)	108.8250(10)	95.0160(10)
γ, °	90	105.1290(10)	110.595(2)	67.8790(10)	100.2900(10)	90
V, Å^3	4899.6(2)	1167.54(4)	1260.0(2)	1196.51(13)	1041.28(8)	2612.90(10)
D_{calc} (g cm^{-1})	1.284	1.717	1.632	1.744	1.733	1.590
Linear absorption, μ (cm^{-1})	3.02	12.04	10.16	12.86	12.15	10.57
F(000)	1984	604	624	628	544	1256
2θmax, °	58	58	58	58	58	58
Reflections measured	76058	29751	26306	14793	12684	27260
Independent reflections	6515	6207	6698	6351	5529	6935
Observed reflections [I > 2σ(I)]	5088	5874	5149	5536	5154	6001
Parameters	290	298	325	305	271	316
R1	0.0353	0.0219	0.0406	0.0289	0.0224	0.0268
wR2	0.0995	0.0564	0.0858	0.0625	0.0538	0.0616
GOF	1.051	1.060	1.015	1.038	1.045	1.048
$\Delta\rho_{max}$/ $\Delta\rho_{min}$ (e Å^{-3})	0.394/–0.324	1.179/–0.412	1.891/–0.759	0.512/–0.724	0.478/–0.705	0.611/–0.645
CCDC	2435219	2435227	2435218	2435221	2435228	2435226

	**13a**	**13b**	**16**	**18**	**(*R*)-17**	**(*R*)-18**
Empirical formula	$C_{46}H_{48}Cl_2N_2O_3.P_2Pd_2S_4$	$C_{16}H_{17}ClNOPPdS_2$	$C_{16}H_{21}ClNPS$	$C_{22}H_{22}ClN_2OPPdS$	$C_{22}H_{23}N_2OPS$	$C_{22}H_{22}ClN_2OPPdS$
Formula weight	1150.74	476.24	325.82	535.29	394.45	535.29
T, K	120	120	100	100	100	100
Crystal system	Triclinic	Triclinic	Orthorhombic	Triclinic	Tetragonal	Monoclinic
Space group	$P\bar{1}$	$P\bar{1}$	Pbcn	$P\bar{1}$	$P4_12_12$	$P2_1$
Z	1	2	8	4	8	4
a, Å	9.8576(14)	8.9669(16)	24.2221(9)	9.2415(2)	9.6972(2)	14.7819(3)
b, Å	10.9011(15)	9.7980(18)	7.3629(3)	14.6006(3)	9.6972(2)	9.6639(2)
c, Å	12.3369(17)	11.281(2)	18.8427(7)	16.9538(4)	43.3305(15)	16.6182(4)
α, °	114.643(2)	81.526(4)	90	78.5360(10)	90	90
β, °	94.267(2)	88.513(3)	90	89.7050(10)	90	109.5830(10)
γ, °	100.210(2)	65.950(4)	90	76.4570(10)	90	90
V, $Å^3$	1169.5(3)	894.6(3)	3360.5(2)	2177.57(8)	4074.6(2)	2236.61(9)
D_{calc} ($g\ cm^{-1}$)	1.634	1.768	1.288	1.633	1.286	1.590
Linear absorption, μ (cm^{-1})	11.73	15.11	4.37	11.61	2.52	11.3
F(000)	582	476	1376	1080	1664	1080
2θmax, °	58	58	54	58	58	56
Reflections measured	22401	7413	35958	27634	27555	45073
Independent reflections	6232	4698	3674	11505	5418	10787
Observed reflections [$I > 2\sigma(I)$]	5270	3780	2551	8482	4987	10420
Parameters	282	209	183	626	247	539
R1	0.0259	0.0377	0.0688	0.069	0.0335	0.0248
wR2	0.0632	0.0823	0.1495	0.1276	0.0764	0.0583
GOF	1.024	0.992	1.038	1.099	1.057	1.049
$\Delta\rho_{max}/\Delta\rho_{min}$ (e $Å^{-3}$)	0.646/–0.577	0.750/–0.762	0.505/–0.469	1.085/–1.399	0.312/–0.229	0.903/–0.482
CCDC	2435225	2435217	2435224	2435222	2435220	2435223

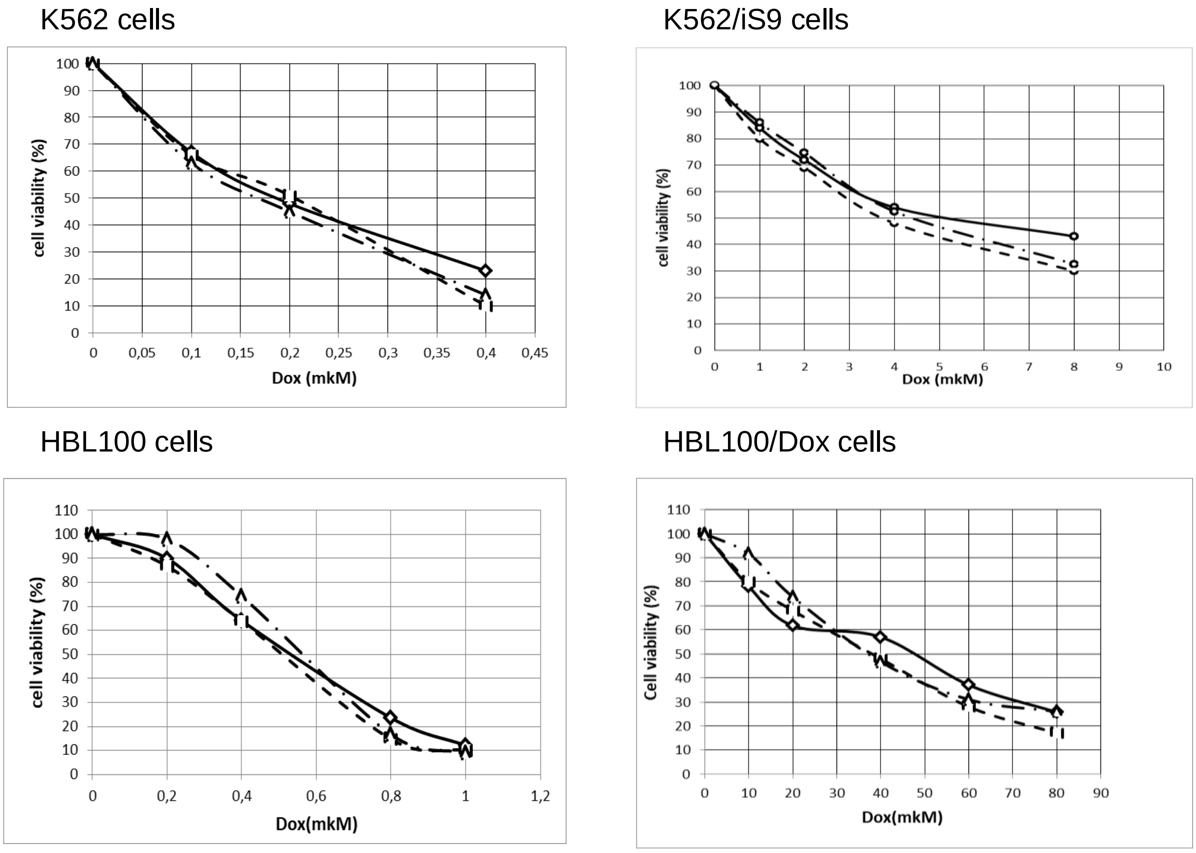

Figure S59. Cytotoxic activity of doxorubicin against parental and doxorubicin-resistant cells upon combined incubation with subtoxic concentrations of complexes **(*R*)-18** and **18** (2 μM for K562 and K562/iS9 cells, 5 μM for HBL100 and HBL100/Dox cells) according to the results of the MTT assay (Dox – solid line, Dox + **18** (dashed line), Dox + **(*R*)-18** (dash-dotted line)
